# Supplementary material for: Increased perinatal survival and improved ventilation skills over a five-year period: An observational study
Source: PLoS One. 2020 Oct 12;15(10):e0240520. doi: 10.1371/journal.pone.0240520 (PMC7549771; doi:10.1371/journal.pone.0240520)
Supplement: Attachment — Submitted filename: PONE-D-19-30351_reviewer.pdf [file pone.0240520.s001.pdf]

PLOS ONE

Increased perinatal survival and improved ventilation skills over a five-year period

--Manuscript Draft--

**Manuscript Number:**	PONE-D-19-30351
**Article Type:**	Research Article
**Full Title:**	Increased perinatal survival and improved ventilation skills over a five-year period
**Short Title:**	Perinatal survival and ventilation skills
**Corresponding Author:**	Ketil Størdal<br>Norwegian Institute of Public Health<br>Oslo, NORWAY
**Keywords:**	Neonate; newborn; Resuscitation; bag-mask ventilation; survival; helping babies breathe; HBB; perinatal mortality; stillbirth; Tanzania
**Abstract:**	Background and aim:<br>The Helping Babies Breathe program gave major reductions in perinatal mortality in Tanzania. We aimed to study whether this effect was sustained, and whether resuscitation skills changed with continued frequent training.<br>Methods:<br>We analysed prospective data covering all births (n=19,571) at Haydom Lutheran Hospital in Tanzania from July 2013 – June 2018. All deliveries were monitored by an observer recording the timing of events and resuscitation interventions. Heart rate was recorded by dry-electrode ECG and bag-mask-ventilation by sensors attached to the resuscitator device. We analysed changes over time in outcomes, use of resuscitation interventions and performance of resuscitation.<br>Results:<br>With introduction of user fees for deliveries since 2014, the number of deliveries decreased by 30% from start to the end of the five-year period. An increase in complicated pregnancies, preterm deliveries, foetal heart rate abnormalities, low heart rate at birth and need for bag-mask-ventilation indicate a gradual selection of more vulnerable newborns delivered in the hospital over time.<br>Despite this selection, newborn deaths <24 hours did not change significantly and was maintained at an average of 8.8/1000 live births. The annual reductions in relative risk for perinatal death adjusted for vulnerability factors was 0.84 (95%CI 0.76-0.94).<br>Ventilation monitoring showed longer ventilation sequences without interruption and increasing duration of ventilation the first minute by time. We also found significantly higher tidal volumes and lower mask leak. The time to initiation or total duration of ventilation did not change significantly.<br><br>Conclusion:<br>The reduction in 24-hour newborn mortality after introduction of Helping Babies Breathe was maintained, with a further decrease after adjustment for vulnerability of the newborns. Perinatal survival and performance of ventilation were significantly improved. Lower rates of births in a safe hospital environment observed during this period is of concern.
**Order of Authors:**	Ketil Størdal
	Kari Holte
	Joar Eilevstjønn
	Estomih Mduma
	Monica Thallinger
	Jørgen Linde
	Paschal Mdoe
	Hussein Kidanto
	Hege Langli Ersdal

Powered by Editorial Manager® and ProduXion Manager® from Aries Systems Corporation

Additional Information:	
**Question**	**Response**
**Financial Disclosure**<br><br>Enter a financial disclosure statement that describes the sources of funding for the work included in this submission. Review the submission guidelines for detailed requirements. View published research articles from *PLOS ONE* for specific examples.<br><br>This statement is required for submission and **will appear in the published article** if the submission is accepted. Please make sure it is accurate.<br><br>**Unfunded studies**<br>Enter: *The author(s) received no specific funding for this work.*<br><br>**Funded studies**<br>Enter a statement with the following details:<br>• Initials of the authors who received each award<br>• Grant numbers awarded to each author<br>• The full name of each funder<br>• URL of each funder website<br>• Did the sponsors or funders play any role in the study design, data collection and analysis, decision to publish, or preparation of the manuscript?<br>• **NO** - Include this sentence at the end of your statement: *The funders had no role in study design, data collection and analysis, decision to publish, or preparation of the manuscript.*<br>• **YES** - Specify the role(s) played.<br><br>* typeset	No
**Competing Interests**<br><br>Use the instructions below to enter a competing interest statement for this submission. On behalf of all authors, disclose any competing interests that could be perceived to bias this work—acknowledging all financial support and any other relevant financial or non-financial competing interests.	No

Powered by Editorial Manager® and ProduXion Manager® from Aries Systems Corporation

This statement **will appear in the published article** if the submission is accepted. Please make sure it is accurate. View published research articles from *PLOS ONE* for specific examples.<br><br>**NO authors have competing interests**<br><br>Enter: *The authors have declared that no competing interests exist.*<br><br>**Authors with competing interests**<br><br>Enter competing interest details beginning with this statement:<br><br>*I have read the journal's policy and the authors of this manuscript have the following competing interests: [insert competing interests here]*<br><br>* typeset	
**Ethics Statement**<br><br>Enter an ethics statement for this submission. This statement is required if the study involved:<br><br>• Human participants<br>• Human specimens or tissue<br>• Vertebrate animals or cephalopods<br>• Vertebrate embryos or tissues<br>• Field research<br><br>Write "N/A" if the submission does not require an ethics statement.<br><br>General guidance is provided below. Consult the submission guidelines for detailed instructions. **Make sure that all information entered here is included in the Methods section of the manuscript.**	Included in the manuscript and uploaded as a letter of permission from the National Institute of Medical Research, Tanzania.

**Format for specific study types**<br><br>**Human Subject Research (involving human participants and/or tissue)**<br>• Give the name of the institutional review board or ethics committee that approved the study<br>• Include the approval number and/or a statement indicating approval of this research<br>• Indicate the form of consent obtained (written/oral) or the reason that consent was not obtained (e.g. the data were analyzed anonymously)<br><br>**Animal Research (involving vertebrate animals, embryos or tissues)**<br>• Provide the name of the Institutional Animal Care and Use Committee (IACUC) or other relevant ethics board that reviewed the study protocol, and indicate whether they approved this research or granted a formal waiver of ethical approval<br>• Include an approval number if one was obtained<br>• If the study involved *non-human primates*, add *additional details* about animal welfare and steps taken to ameliorate suffering<br>• If anesthesia, euthanasia, or any kind of animal sacrifice is part of the study, include briefly which substances and/or methods were applied<br><br>**Field Research**<br><br>Include the following details if this study involves the collection of plant, animal, or other materials from a natural setting:<br>• Field permit number<br>• Name of the institution or relevant body that granted permission	
**Data Availability**<br><br>Authors are required to make all data underlying the findings described fully available, without restriction, and from the time of publication. PLOS allows rare exceptions to address legal and ethical concerns. See the PLOS Data Policy and FAQ for detailed information.	No - some restrictions will apply

Powered by Editorial Manager® and ProduXion Manager® from Aries Systems Corporation

A Data Availability Statement describing where the data can be found is required at submission. Your answers to this question constitute the Data Availability Statement and **will be published in the article**, if accepted.<br><br>**Important:** Stating 'data available on request from the author' is not sufficient. If your data are only available upon request, select 'No' for the first question and explain your exceptional situation in the text box.<br><br>Do the authors confirm that all data underlying the findings described in their manuscript are fully available without restriction?	
**Describe where the data may be found in full sentences. If you are copying our sample text, replace any instances of XXX with the appropriate details.**<br><br>• If the data are **held or will be held in a public repository**, include URLs, accession numbers or DOIs. If this information will only be available after acceptance, indicate this by ticking the box below. For example: *All XXX files are available from the XXX database (accession number(s) XXX, XXX.)*.<br>• If the data are all contained **within the manuscript and/or Supporting Information files**, enter the following: *All relevant data are within the manuscript and its Supporting Information files.*<br>• If neither of these applies but you are able to provide **details of access elsewhere**, with or without limitations, please do so. For example:<br><br>*Data cannot be shared publicly because of [XXX]. Data are available from the XXX Institutional Data Access / Ethics Committee (contact via XXX) for researchers who meet the criteria for access to confidential data.*<br><br>*The data underlying the results presented in the study are available from (include the name of the third party*	Data cannot be shared publicly because of personal integrity and regulations from the National Institute of Medical Research in Tanzania (NIMR). Data are available from the Safer Births Institutional Data Access / Ethics Committee (contact via hege.ersdal@safer.net) for researchers who meet the criteria for access to confidential data.

Powered by Editorial Manager® and ProduXion Manager® from Aries Systems Corporation

and contact information or URL).<br>• This text is appropriate if the data are owned by a third party and authors do not have permission to share the data.<br><br>* typeset	
Additional data availability information:	

Powered by Editorial Manager® and ProduXion Manager® from Aries Systems Corporation

Manuscript

Click here to access/download;Manuscript;Submitted manuscript.docx

Increased perinatal survival and improved ventilation skills over a five-year period

Ketil Størdal[1,2*], Joar Eilevstjønn[3], Estomih Mduma[4], Kari Holte[2], Monica Thallinger[5], Jørgen Linde[6], Paschal Mdoe[7], Hussein Kidanto[8], Hege Ersdal[6,9]

[1] Norwegian Institute of Public Health, Oslo, Norway.

[2] Department of Pediatrics, Østfold Hospital Trust, Grålum, Norway.

[3] Laerdal Medical AS, Strategic Research, Stavanger, Norway

[4] Haydom Lutheran Hospital, Research Centre, Manyara, Tanzania.

[5] Vestre Viken HF, Dpt of Anaesthesiology, Gjettum, Norway.

[6] Helse Stavanger HF, Dpt of Pediatrics, Stavanger, Norway.

[7] Haydom Lutheran Hospital, Maternity Ward, Manyara, Tanzania.

[8] Muhimbili National Hospital, Dar Es Salaam, Tanzania.

[9] SAFER, Stavanger, Norway.

*Corresponding author

E-mail: ketil.stordal@fhi.no (KS)

Abstract

Background and aim:

The Helping Babies Breathe program gave major reductions in perinatal mortality in Tanzania. We aimed to study whether this effect was sustained, and whether resuscitation skills changed with continued frequent training.

Methods:

We analysed prospective data covering all births (n=19,571) at Haydom Lutheran Hospital in Tanzania from July 2013 – June 2018. All deliveries were monitored by an observer recording the timing of events and resuscitation interventions. Heart rate was recorded by dry-electrode ECG and bag-mask-ventilation by sensors attached to the resuscitator device. We analysed changes over time in outcomes, use of resuscitation interventions and performance of resuscitation.

Results:

With introduction of user fees for deliveries since 2014, the number of deliveries decreased by 30% from start to the end of the five-year period. An increase in complicated pregnancies, preterm deliveries, foetal heart rate abnormalities, low heart rate at birth and need for bag-mask-ventilation indicate a gradual selection of more vulnerable newborns delivered in the hospital over time.

Despite this selection, newborn deaths <24 hours did not change significantly and was maintained at an average of 8.8/1000 live births. The annual reductions in relative risk for perinatal death adjusted for vulnerability factors was 0.84 (95%CI 0.76-0.94).

Ventilation monitoring showed longer ventilation sequences without interruption and increasing duration of ventilation the first minute by time. We also found significantly higher tidal volumes and lower mask leak. The time to initiation or total duration of ventilation did not change significantly.

Conclusion:

The reduction in 24-hour newborn mortality after introduction of Helping Babies Breathe was maintained, with a further decrease after adjustment for vulnerability of the newborns. Perinatal survival and performance of ventilation were significantly improved. Lower rates of births in a safe hospital environment observed during this period is of concern.

Introduction

In an era with declining mortality from 1 month to five years, the reduction in neonatal deaths has been slower.[1] The estimated global neonatal mortality (≤ 28 days) has fallen from 31/1000 in 2000 to 18/1000 in 2017.[2] Due to the rapid decline in mortality after the neonatal period, the relative contribution of neonatal deaths in the global under-5-mortality has increased from 39% to 45%.[1] The majority of these deaths are preventable. Safer births can be obtained by improvements in a continuum starting with pregnancy care, obstetric care and neonatal care, each accounting for 1/3 of preventable deaths.[3]

Intrapartum-related events, formerly referred to as birth asphyxia and defined as inadequate onset of spontaneous breathing after birth, is one of the three most prevalent causes of under-5-mortality.[1] The Helping Babies Breathe (HBB) program was developed to reduce death from intrapartum events in low- and middle-income settings, through training of health care workers in early identification and treatment.[4]

In Tanzania, HBB was rolled out from 2010, with a 47% reduction in fresh stillbirths and early neonatal mortality seen after 24 months in eight large birth facilities.[5] Haydom Lutheran Hospital was one of the eight sites participating in the early roll-out of HBB. Since the launch of HBB at Haydom in 2010, HBB training and research related to safer deliveries and newborn resuscitations have continued in the Safer Births program.[6]

Tanzania has seen a reduction in neonatal mortality from 33/1000 in 2000 to 21/1000 in 2017.[2] Vital birth statistics are, however, in general not available,[7,8] and estimates continue to be inaccurate as almost half of the deliveries in Tanzania take place outside birth facilities. In a rural area with a low proportion of deliveries in hospital, this selection may preclude interpretation of temporal trends in mortality rates. It is therefore pertinent to adjust for vulnerability background factors in hospital deliveries.

In the present study, we aimed to document changes and predictors of 24-hour neonatal mortality and fresh stillbirths during a five-year period, after introduction of frequent HBB

trainings in 2011. We further studied changes in resuscitation performance in this facility with an ongoing onsite low-dose high-frequency HBB training focus.

Methods

Haydom Lutheran Hospital is situated in the rural area of the Manyara region of Northern Tanzania. The hospital has a catchment area of over 2 million inhabitants, and is the only hospital in the region providing comprehensive obstetric care with 250-400 deliveries per month. Haydom is a non-governmental hospital managed by the Lutheran church of Tanzania. Due to financial constraints, delivering mothers have since the beginning of 2014 been charged with a fee of 12 USD for vaginal deliveries and 30 USD for caesarean section in addition to fees for ambulance transport. The number of births in the hospital has thereafter fallen substantially, likely due to the costs of delivery care.[6]

The local procedure for newborn resuscitation followed the HBB guidelines, emphasizing stimulation and early initiation of bag-mask ventilation (BMV).[4] Newborn resuscitation was mainly the responsibility of midwives. Prior to and during the study all midwives were encouraged to low-dose, high-frequency skills training as described by Mduma et al.[9] There were six labour rooms, each equipped with a resuscitation table. After caesarean section, newborn resuscitation was performed in a room adjacent to the operating theatre. After resuscitation, the midwives decided based on the clinical condition whether to keep the newborn with the mother or transfer to a neonatal ward providing basic care without ventilation support equipment except supplemental oxygen on nasal prongs.

The Safer Births program, starting in 2013, aimed to further facilitate HBB training and improve obstetric and newborn care by low-cost technology.[6,10] Trained data collectors observed and recorded the timing of events, and vital data and outcomes from all deliveries were recorded in a database.

Since 1st of July 2013 recordings of heart rate data by ECG dry electrodes and ventilation parameters by a flow sensor connected to the ventilation bag have been stored for research purposes. Details about the monitors and the frequent HBB training has been published elsewhere.[11] We analysed data for a five years period with collection of physiological data until 30th of June 2018 (Fig 1, flow chart).

For the analysis of ventilation parameters, we excluded those ventilated with a valve providing PEEP (Positive End Expiratory Pressure) in a trial enrolling participants from September 2016 to June 2018, because this intervention is likely to change mean airway pressures and tidal volumes.

Main outcome and exposure

The main outcome was early neonatal mortality (ENM) defined as death before 24 hours of life among newborns delivered alive at Haydom Lutheran Hospital. As shown previously, live born babies with severe asphyxia may be misclassified as fresh stillbirths (FSB).[12,13] Therefore, we also used the combined outcome of ENM and FSB to analyse perinatal deaths. We analysed by year (1st of July – 30th of June, starting 1st of July 2013) for the five-year period to assess effects of ongoing training in newborn resuscitation over time.

Vulnerability factors

The reduction in deliveries at the hospital during the period, was likely to introduce a selection bias. More complicated deliveries were likely conducted or transferred to the hospital with impact on mortality rates of those delivering in the hospital. To account for potential vulnerability factors, we adjusted mortality rates for pregnancy complications, birth weight, prematurity, foetal heart rate abnormalities, last recorded foetal heart rate and the need for BMV at birth.

Neonatal resuscitation performance

We aimed to study whether frequent training on neonatal resuscitation through the HBB program affected clinical performance. We analysed timing of events by studying time to start of BMV, duration of the first ventilation sequence before pause (>5 seconds interruption) and fraction of time used for BMV once initiated. To assess ventilation performance, we studied ventilation frequency, tidal volumes and mask leak. Heart rate change after 60 seconds adjusted for baseline was also studied.

Statistical methods

Descriptive analyses were performed by calculation of medians with interquartile ranges (IQR) or means with standard deviations according to their distribution.

We used binary linear regression to estimate relative risks for ENM by time. Vulnerability factors which showed changes over time and were associated with the outcome ($p<0.10$) were included in a multivariate regression model.

We also performed sensitivity analyses excluding newborns who died without a history of resuscitation at birth because other conditions than intra-partum hypoxia may cause ENM.

Ethical consideration

This study was approved by the National Institute for Medical Research, Tanzania (NIMR/HQ/R8a/Vol. IX/1434) and the Regional Committee for Medical and Health Research Ethics, Western Norway (2013/110/REK). All health care providers gave informed oral consent. Patients were informed, but consent was not obtained for this descriptive study as approved by the ethical committees.

Results

During the five-year period, 18,997 infants were born alive, with a reduction over time from 4,662 to 3,197 annual births (Table 1). ENM fluctuated around 8.8/1000 live births, and the combined outcome of FSB and ENM around 22.6/1000 with no significant differences over time (Table 2).

Vulnerability factors over time

Concomitant with a 31% reduction in deliveries in the hospital during the observation period, a higher proportion of deliveries was characterized by risk factors for adverse outcome. The proportion of pregnancies with antenatal problems including preeclampsia increased significantly from <1% to >2%. The mean birth weight increased slightly, but an increasing proportion were <1500 g and preterm births increased from 2.5% to 3.5% (Table 1). Abnormal foetal heart rate during delivery increased gradually from 3% to 9%. The first detected heart rate among resuscitated babies fell from a median of 142 to 108 beats per minute (bpm), and the proportion with heart rate <100 bpm at birth increased from 8% to 22% (Table 3).

Mortality adjusted for vulnerability

In unadjusted analyses, the ENM increased slightly during the five-year period (relative risk per year 1.11, 95%CI 1.00-1.24, Table 4). Vulnerability factors all increased during this period, as shown in Table 1 and 3. After adjustment for abnormal foetal heart rate during delivery, birth weight/gestational age, initial heart rate and need for BMV at birth, there was an annual non-significant 5% reduction in relative risk for ENM (relative risk per year 0.95, 95%CI 0.84-1.07, Table 4). Death the first 24 hours could be due to other reasons than intrapartum events, especially prematurity. Of those who died, 55/169 (33%) had not been ventilated, and thus would likely not be diagnosed as asphyxiated. We repeated the analyses without these 55: The unadjusted and adjusted analyses were essentially unchanged (data not shown).

The combined outcome of perinatal mortality (FSB and ENM) showed no appreciable change during the five-year period in unadjusted analyses. However, after adjustment for vulnerability factors we found an annual decrease of 16% in perinatal mortality (relative risk per year 0.84, 95%CI 0.76-0.94, Table 4).

In the fully adjusted model, year of birth, foetal heart rate abnormalities, initial heart rate, birth weight, prematurity and need for BMV predicted perinatal death (Table 5).

Changes in resuscitation performance

The proportion who received BMV varied from 6.1-7.7%, but increased to 8.8% the last year (Table 3). Time from birth to start of BMV was lowest the last year (median 99 seconds) but did not change significantly over time (p=0.76). The duration of the first BMV sequence increased over time from a median of 11 to 32 seconds, and active ventilation continued for an increasing part of the first 60 seconds. The duration of ventilation from the first to the last inflation remained stable (Table 3).

Median ventilation frequency decreased from >60/min the first year to 44-48/min the last three years of observation (p<0.001). The median tidal volume, mean and peak airway pressures increased, whereas mask leak decreased slightly but significantly over time (Table 3).

Discussion

During a five-year period following a successful implementation of frequent HBB training, we found no further significant decline in ENM. A worrying decrease in hospital deliveries was observed, likely due to introduction of user fees, and newborns with a higher vulnerability for adverse outcome were born in the hospital. In analyses adjusted for vulnerability factors, perinatal deaths decreased during the five years.

Strengths and limitations

Among several strengths in the study is the large size population-based study, performed in a rural setting of a low-income country with high burden of disease. The prospective design ensured high-quality data from the delivery and immediate post-natal period. Collection of physiological data during the first minutes of life provides information which is rarely accessible.

The selection of higher-risk deliveries during the period is evident, and accounted for as far as possible in the adjusted analysis. However, as in any observational study, residual confounding cannot be completely ruled out. A population-based birth registry with inclusion of all pregnancies including home deliveries would capture the complete picture and avoid selection bias. Specific causes of ENM were not possible to identify in our study, though in a sub-study from this cohort half of the deaths were related to intra-partum events.[14] We were, however, able to exclude deaths unlikely to be caused by perinatal hypoxia in a sensitivity analysis.

Comparison with other studies

During the nation-wide HBB roll-out during 2010/2011 in Tanzania, ENM in eight large hospitals including Haydom decreased from 13.4 to 7.1/1000. FSB decreased from 19 to 14.4/1000, and a remarkable fall of the combined outcome of FSB and ENM of 34% was reported.[5] ENM in Haydom before and after implementation of HBB in 2011 was 11.1 and 7.2/1000, respectively.[9] The baseline perinatal mortality was 27/1000 and fell to 21.7/1000.[15] In a follow-up study, this reduction was maintained until end of observation in January 2017.[6] In the present extended follow-up, our period of observation starts two years after regular HBB implementation and extends further 1.5 years. Our study shows that the initial reduction in perinatal mortality seen after HBB implementation has continued.

HBB has been introduced globally, and overall a reduction in perinatal mortality has been reported.[16] The variation between countries with highly successful implementation [5,17,18] to sites reporting no or modest effect on mortality [19,20] is of note. The relative contribution of intra-partum hypoxia to ENM across countries may explain some differences. Besides, implementation and uptake of HBB could influence the results, and repeated low-dose onsite training is likely to facilitate successful implementation.[9] Several studies have reported improved resuscitation knowledge and practical skills after HBB training in a simulation setting.[4,21] Some loss of performance over time is observed,[22-24] but skills retention was improved by frequent re-training and use of self-evaluation checklists.[23] Importantly, changes in clinical management do not necessarily follow simulated performance.[25]

In a study from Nepal, a reduction of deaths due to intra-partum related complications was observed, but overall newborn mortality was unchanged.[26] This was also reported in the recent meta-analysis, pointing to the importance of a package of interventions needed to address the main causes of neonatal death.[16] In our study, one third of deaths occurred in

newborns, mainly preterms, that did not receive BMV after birth. Intra-partum events is the leading cause of death at Haydom, but preterm birth and sepsis are among other preventable causes of death.[14]

Our study is the first to report long-term sustained effect on mortality after HBB implementation. Analyses of actual ventilation performance demonstrate that practical skills improved over time with significantly longer duration of the first ventilation sequence and reduction in ventilation frequency targeting the recommended range. The reduction in mask leak over time was also desirable though admittedly small. Higher tidal volumes are associated with more rapid HR increase in observational studies from Haydom,[27,28] but should be further studied in controlled trials before we can conclude whether this is recommendable.

In the initial HBB roll-out at Haydom, the use of suction/stimulation increased whereas use of BMV decreased significantly.[9] We found a further increase in suction/stimulation but also of BMV, which could be expected with increased vulnerability over time. In a meta-analysis of the effect of HBB training on resuscitation practices from four studies, the overall use of suctioning, stimulation or BMV was unchanged. However, interventions during the first Golden minute increased 2.5-fold.[29] In our study the reduced time from birth to BMV initiation the last year of observation is of note.

Implications

In the present study the quality of immediate newborn care at birth was improved over time, probably due to continuous focus on frequent HBB training. However, coverage and access are likely even more important for the total perinatal mortality than quality of care in the

hospital. These factors are interrelated, as increased quality in a delivery unit is likely to encourage more pregnant women to seek care. However, financial constraints may counteract this effect. In Tanzania in 2011/12, 44% had less than 1.25 USD per day for their daily living, and poverty was even more prevalent in rural districts.[30] In this context, 12 to 30 USD is a considerable burden to the family economy. Free delivery care should be a high priority, as provided in governmental hospitals in Tanzania.

In this study we have shown that neonatal resuscitation skills improved over time at a site with high focus on continued and frequent HBB training. The perinatal mortality adjusted for vulnerability factors decreased, whereas ENM showed a non-significant decline. Further reduction in neonatal mortality in Tanzania will likely require a comprehensive approach to increase facility births and address the major causes of death.

1. Liu L, Oza S, Hogan D, et al. Global, regional, and national causes of under-5 mortality in 2000-15: an updated systematic analysis with implications for the Sustainable Development Goals. *Lancet.* 2016;388(10063):3027-3035.
2. Hug L, Alexander M, You D, Alkema L, Estimation UNI-aGfCM. National, regional, and global levels and trends in neonatal mortality between 1990 and 2017, with scenario-based projections to 2030: a systematic analysis. *Lancet Glob Health.* 2019;7(6):e710-e720.
3. Bhutta ZA, Das JK, Bahl R, et al. Can available interventions end preventable deaths in mothers, newborn babies, and stillbirths, and at what cost? *Lancet.* 2014;384(9940):347-370.
4. Singhal N, Lockyer J, Fidler H, et al. Helping Babies Breathe: global neonatal resuscitation program development and formative educational evaluation. *Resuscitation.* 2012;83(1):90-96.
5. Msemo G, Massawe A, Mmbando D, et al. Newborn mortality and fresh stillbirth rates in Tanzania after helping babies breathe training. *Pediatrics.* 2013;131(2):e353-360.
6. Mduma E, Kvaloy JT, Soreide E, et al. Frequent refresher training on newborn resuscitation and potential impact on perinatal outcome over time in a rural Tanzanian hospital: an observational study. *BMJ Open.* 2019;9(9):e030572.
7. Rebnord T, Ostbye T, Mmbaga BT, McHome B, Lie RT, Daltveit AK. Time trends in management of HIV-positive pregnant women in Northern Tanzania: A registry-based study. *PLoS One.* 2017;12(9):e0184362.
8. Bergsjo P, Mlay J, Lie RT, Lie-Nielsen E, Shao JF. A medical birth registry at Kilimanjaro Christian Medical Centre. *East Afr J Public Health.* 2007;4(1):1-4.
9. Mduma E, Ersdal H, Svensen E, Kidanto H, Auestad B, Perlman J. Frequent brief on-site simulation training and reduction in 24-h neonatal mortality--an educational intervention study. *Resuscitation.* 2015;93:1-7.
10. http://www.saferbirths.com/. Accessed 04.10.2019.
11. Linde JE, Schulz J, Perlman JM, et al. Normal Newborn Heart Rate in the First Five Minutes of Life Assessed by Dry-Electrode Electrocardiography. *Neonatology.* 2016;110(3):231-237.
12. Thallinger M, Ersdal HL, Francis F, et al. Born not breathing: A randomised trial comparing two self-inflating bag-masks during newborn resuscitation in Tanzania. *Resuscitation.* 2017;116:66-72.
13. Ersdal HL, Eilevstjonn J, Linde JE, et al. Fresh stillborn and severely asphyxiated neonates share a common hypoxic-ischemic pathway. *Int J Gynaecol Obstet.* 2017.
14. Moshiro R, Perlman JM, Mdoe P, Kidanto H, Kvaloy JT, Ersdal HL. Potential causes of early death among admitted newborns in a rural Tanzanian hospital. *PLoS One.* 2019;14(10):e0222935.
15. Mduma ER, Ersdal H, Kvaloy JT, et al. Using statistical process control methods to trace small changes in perinatal mortality after a training program in a low-resource setting. *Int J Qual Health Care.* 2018;30(4):271-275.
16. Versantvoort JMD, Kleinhout MY, Ockhuijsen HDL, Bloemenkamp K, de Vries WB, van den Hoogen A. Helping Babies Breathe and its effects on intrapartum-related stillbirths and neonatal mortality in low-resource settings: a systematic review. *Arch Dis Child.* 2019.
17. Kc A, Wrammert J, Clark RB, et al. Reducing Perinatal Mortality in Nepal Using Helping Babies Breathe. *Pediatrics.* 2016;137(6).
18. Arabi AME, Ibrahim SA, Manar AR, et al. Perinatal outcomes following Helping Babies Breathe training and regular peer-peer skills practice among village midwives in Sudan. *Arch Dis Child.* 2018;103(1):24-27.
19. Bellad RM, Bang A, Carlo WA, et al. A pre-post study of a multi-country scale up of resuscitation training of facility birth attendants: does Helping Babies Breathe training save lives? *BMC Pregnancy Childbirth.* 2016;16(1):222.
20. Goudar SS, Somannavar MS, Clark R, et al. Stillbirth and newborn mortality in India after helping babies breathe training. *Pediatrics.* 2013;131(2):e344-352.

21. Musafili A, Essen B, Baribwira C, Rukundo A, Persson LA. Evaluating Helping Babies Breathe: training for healthcare workers at hospitals in Rwanda. *Acta Paediatr.* 2013;102(1):e34-38.
22. Bang A, Patel A, Bellad R, et al. Helping Babies Breathe (HBB) training: What happens to knowledge and skills over time? *BMC Pregnancy Childbirth.* 2016;16(1):364.
23. Kc A, Wrammert J, Nelin V, et al. Evaluation of Helping Babies Breathe Quality Improvement Cycle (HBB-QIC) on retention of neonatal resuscitation skills six months after training in Nepal. *BMC Pediatr.* 2017;17(1):103.
24. Arlington L, Kairuki AK, Isangula KG, et al. Implementation of "Helping Babies Breathe": A 3-Year Experience in Tanzania. *Pediatrics.* 2017;139(5).
25. Ersdal HL, Vossius C, Bayo E, et al. A one-day "Helping Babies Breathe" course improves simulated performance but not clinical management of neonates. *Resuscitation.* 2013;84(10):1422-1427.
26. Wrammert J, Kc A, Ewald U, Malqvist M. Improved postnatal care is needed to maintain gains in neonatal survival after the implementation of the Helping Babies Breathe initiative. *Acta Paediatr.* 2017;106(8):1280-1285.
27. Moshiro R, Perlman JM, Kidanto H, Kvaloy JT, Mdoe P, Ersdal HL. Predictors of death including quality of positive pressure ventilation during newborn resuscitation and the relationship to outcome at seven days in a rural Tanzanian hospital. *PLoS One.* 2018;13(8):e0202641.
28. Linde JE, Schulz J, Perlman JM, et al. The relation between given volume and heart rate during newborn resuscitation. *Resuscitation.* 2017;117:80-86.
29. Budhathoki SS, Gurung R, Ewald U, Thapa J, Kc A. Does the Helping Babies Breathe Programme impact on neonatal resuscitation care practices? Results from systematic review and meta-analysis. *Acta Paediatr.* 2019;108(5):806-813.
30. *Tanzania Mainland poverty assessment.* 2015.

Table 1: Baseline characteristics of live births at Haydom 1.7.2013-30.6.2018 (n=18,997)

	Period of birth					
	**1st year** n=4,662	**2nd year** n=3,909	**3rd year** n=3,565	**4th year** n=3,664	**5th year** n=3,197	p-value*
**Maternal characteristics**						
No antenatal care, % (n)						0.16
	0.9 (44)	1.2 (48)	1.0 (36)	0.7 (24)	1.0 (32)	
Antenatal problem, % (n)						<0.001
	0.9 (44)	0.7 (29)	1.6 (57)	1.8 (65)	2.1 (66)	
Preeclampsia/eclampsia, % (n)						0.07
	0.5 (21)	0.4 (17)	0.7 (25)	0.7 (26)	0.9 (28)	
**Delivery**						
Mode of delivery,% (n)						<0.001
Vaginal	78 (3,650)	76 (2,961)	76 (2,715)	76 (2,770)	75 (2,409)	
Caesarean	20 (924)	24 (943)	23 (824)	23 (858)	23 (749)	
Breech	2 (77)	0 (0)	0.5 (18)	0.5 (19)	0.8 (26)	
Vacuum/others	0.2 (11)	0.1 (4)	0.2 (8)	0.5 (17)	0.3 (11)	
Fetal heart rate at admission,% (n)						<0.001
Normal	88 (4,122)	90 (3,510)	92 (3,274)	94 (3,444)	94 (3,006)	
Abnormal	1 (42)	1 (33)	1 (31)	1 (38)	1 (43)	
Not detectable	0.04 (2)	0 (0)	0.1 (2)	0.1 (5)	0 (0)	
Not measured	11 (496)	9 (365)	7 (257)	5 (177)	5 (145)	
Fetal heart rate during labour,% (n)						<0.001
Normal	84 (3,917)	83 (3,249)	83 (2,973)	86 (3,157)	86 (2,751)	
Abnormal	3 (143)	4 (168)	5 (168)	6 (230)	9 (298)	
Not detectable	0 (0)	0 (0)	0.2 (7)	0.1 (4)	0.03 (1)	
Not measured	13 (602)	13 (494)	12 (416)	7 (272)	5 (145)	
Last heart rate before delivery (bpm), mean (SD)						0.14
	134 (10)	134 (10)	134 (12)	134 (14)	133 (14)	
**Child characteristics**						
Birth weight, Mean (SD)	3215 (528)	3253 (520)	3280 (520)	3377 (539)	3342 (546)	<0.001
<1500	0.5 (24)	0.5 (24)	0.4 (15)	0.6 (23)	0.9 (28)	
1500-2500	7 (325)	6 (218)	6 (199)	4 (158)	4 (136)	
2500-3499	62 (2,895)	61 (2,397)	58 (2,071)	50 (1,821)	53 (1,690)	
3500-4499	29 (1,370)	31 (1,228)	35 (1,242)	44 (1,594)	40 (1,284)	
≥4500	1 (48)	1 (42)	1 (38)	2 (68)	2 (57)	
Gestational age < 37 weeks, % (n)						0.042
	2.5 (117)	2.6 (103)	2.7 (96)	3.3 (121)	3.5 (111)	
Female sex ,% (n)						0.049
	46 (2,141)	48 (1,882)	47 (1,672)	46 (1,692)	48 (1,546)	

* p-values calculated with chi-square for categorical and binary regression for continuous variables

1st year: 1.7.2013-30.6.2014
2nd year: 1.7.2014-30.6.2015
3rd year: 1.7.2015-30.6.2016
4th year: 1.7.2016-30.6.2017
5th year: 1.7.2017-30.6.2018

Table 2: Outcome by year including stillbirths (n=19,587)

	Period of birth					
	**1st year**	**2nd year**	**3rd year**	**4th year**	**5th year**	p-value*
Macerated stillbirths, % (n/total births)						
	1.3 (63/4,801)	1.4 (55/4,022)	1.3 (48/3,652)	2.0 (74/3,794)	2.0 (65/3,302)	0.021
Fresh stillbirths % (n/total births excl MSB)						
	1.6 (76/4,738)	1.5 (58/3,967)	1.1 (39/3,604)	1.5 (56/3,720)	1.2 (40/3,237)	0.27
Early neonatal death, % (n/total of liveborn)						
	0.6 (30/4,662)	1.0 (41/3,909)	0.6 (23/3,565)	1.0 (36/3,664)	1.2 (37/3,197)	0.10
FSB + Early neonatal death, % (n/total of births excl MSB)						
	2.2 (106/4,738)	2.5 (99/3,967)	1.7 (62/3,604)	2.5 (92/3,720)	2.4 (77/3,235)	0.15

* p-values calculated with chi-square test

Table 3: Postnatal characteristics of live births at Haydom 1.7.2013-30.6.2018 (n=18,997)

	Period of birth					
	**1st year**	**2nd year**	**3rd year**	**4th year**	**5th year**	**p-value***
	n=4,662	n=3,909	n=3,565	n=3,664	n=3,197	
Apgar <5, % (n)						
1 min	0.8 (37)	1.3 (50)	1.2 (42)	1.2 (45)	2.1 (66)	<0.001
5 min	0.1 (5)	0.2 (7)	0.3 (11)	0.6 (20)	0.7 (23)	<0.001
Stimulation/suction*, % (n)						<0.001
No stim./suction	85 (3,950)	83 (3,234)	68 (2,412)	73 (2,684)	68 (2,180)	<0.001
Stimulation only	0.3 (15)	0.9 (36)	2.8 (100)	4.3 (159)	6 (186)	
Stim. and suction	15 (697)	16 (641)	30 (1053)	22 (821)	26 (843)	
Bag/mask ventilation	7.7 (361)	6.1 (240)	6.8 (242)	7.4 (272)	8.8 (264)	<0.001
Heart rate	n=389	n=298	n=274	n=262	n=232	
Initial HR/min median (IQR)	145 (97-179)	142 (94-171)	132 (84-174)	118 (70-162)	102 (67-148)	<0.001
Proportion with initial HR in % (n)*						
<60	8 (32)	8 (25)	8 (21)	14 (36)	22 (50)	<0.001
60-100	18 (69)	19 (58)	27 (75)	28 (74)	27 (63)	
100-160	36 (139)	33 (98)	31 (85)	31 (80)	32 (75)	
>160	38 (149)	39 (117)	34 (93)	27 (72)	19 (44)	
HR 60 seconds after first recording, median (IQR)	143 (104-165)	139 (101-165)	139 (105-165)	130 (91-157)	129 (102-152)	<0.001
Bag/mask-ventilation, time events	n=358	n=234	n=240	n=269	n=262	
Time from birth to BMV, seconds median,(IQR)	105 (74-149)	116 (81-164)	119 (88-158)	109 (74-162)	99 (74-153)	0.76
Duration of first BMV sequence, median,(IQR)	11 (4-20)	11 (5-22)	16 (7-34)	23 (9-52)	32 (13-71)	<0.001
Duration of BMV first 60 seconds, median (IQR)	53 (26-59)	55 (35-59)	57 (37-59)	59 (52-59)	59 (47-59)	<0.001
Duration of BMV in total, median (IQR)	132 (56-248)	127 (59-340)	127 (66-298)	142 (64-282)	130 (73-301)	0.94
Bag/mask ventilation characteristics†	n=293	n=247	n=238	n=173	n=114	
Frequency of ventilations (inflations per minute)	66 (50-84)	52 (39-70)	44 (35-53)	48 (41-54)	47 (41-53)	<0.001
Tidal volume in ml/kg, median (IQR)	5.3 (3.3-8.4)	6.8 (4.3-10.0)	7.5(5.0-11.5)	6.6 (4.5-10.7)	6.2 (3.2-9.8)	<0.001
Mask leak in %, median (IQR)	45 (33-62)	46 (33-61)	42 (30-57)	41 (29-56)	44 (30-57)	0.001
Mean airway pressure mbar, median (IQR)	14 (12-17)	13 (11-16)	15 (12-18)	17 (14-21)	18 (16-21)	<0.001
Maximum airway pressure mbar, median (IQR)	34 (29-38)	33 (28-36)	36 (32-39)	38 (34-43)	40(37-45)	<0.001

HR: Heart rate

BMV: Bag-mask ventilation

IQR: Inter-quartile range

* Chi2-test used for categorical analyses, linear regression for continuous analyses. P-values for trend.

† excluding participants in the PEEP-study, n=80 and n=123 in 4th and 5th year, respectively.
Two-step analysis: Mean of ventilation parameters for each newborn during first 5 minutes of active ventilation calculated, and in the second step this mean was used to calculate median/IQR.

Table 4: Relative risk for early neonatal death and perinatal death at Haydom 1.7.2013-30.6.2018

	Period of birth					Overall	p-value†
**Early neonatal deaths, n/total live births**	**1st year** 30/4,662	**2nd year** 41/3,909	**3rd year** 23/3,565	**4th year** 56/3,720	**5th year** 37/3,197	**linear**	
**Unadjusted**							
	1 (ref.)	1.63 (1.02-2.61)	1.00 (0.58-1.72)	1.53 (0.94-2.47)	1.63 (1.11-2.90)	1.11 (1.00-1.24)	0.04
**Adjusted for antenatal problems**							
	1 (ref.)	1.64 (1.02-2.62)	0.99 (0.58-1.70)	1.50 (0.93-2.44)	1.76 (1.09-2.84)	1.11 (1.00-1.23)	0.06
**Adjusted for abnormal fetal heart rate**							
	1 (ref.)	1.55 (0.97-2.48)	0.93 (0.54-1.59)	1.34 (0.83-2.18)	1.44 (0.88-2.33)	1.06 (0.95-1.18)	0.31
**Adjusted for birth weight and prematurity**							
	1 (ref.)	1.58 (1.00-2.48)	1.00 (0.59-1.70)	1.53 (0.95-2.44)	1.72 (1.08-2.72)	1.11 (1.00-1.23)	0.05
**Adjusted for initial heart rate**							
	1 (ref.)	1.72 (1.09-2.70)	0.96 (0.56-1.63)	1.34 (0.84-2.14)	1.41 (0.88-2.26)	1.04 (0.94-1.16)	0.41
**Adjusted for need for BMV**						1.09	
	1 (ref.)	1.88 (1.19-2.98)	1.10 (0.65-1.88)	1.57 (0.98-2.52)	1.72 (1.07-2.74)	1.09 (0.99-1.21)	0.39
**Final adjusted model***							
	1 (ref.)	1.88 (1.11-3.19)	0.92 (0.50-1.69)	1.25 (0.72-2.18)	0.93 (0.53-1.64)	0.95 (0.84-1.07)	0.39
**Perinatal deaths FSB+ENM/total births**	106/4,738	99/3,967	62/3,604	92/3,720	77/3,235		
**Unadjusted**							
	1 (ref.)	1.12 (0.85-1.46)	0.77 (0.56-1.05)	1.11 (0.84-1.46)	1.06 (0.80-1.42)	1.01 (0.95-1.08)	0.77
**Final adjusted model***							
	1 (ref.)	1.58 (1.01-2.47)	0.88 (0.54-1.43)	0.87 (0.55-1.39)	0.53 (0.32-0.86)	0.84 (0.76-0.94)	0.002

* adjusted for antenatal problem, abnormal fetal heart rate during labour, birth weight, prematurity, initial heart rate and need for BMV after birth.

† p-value for trend

Table 5: Predictors for perinatal death at Haydom Lutheran Hospital during five years (n=19,264).

	**Unadjusted**	**Adjusted***
**Period, per year increase 2013-18**		
	1.01 (0.95-1.08)	0.84 (0.76-0.94)
**Fetal heart rate:**		
**normal**	1 (ref.)	1 (ref.)
**abnormal**	5.7 (4.4-7.3)	3.0 (2.0-4.5)
**not detected**	74.0 (64.5-84.9)	62.9 (20.5-193)
**Birth weight**		
**<1.5 kg**	28.7 (21.0-39.2)	23.6 (10.5-53.2)
**1.5-2.5 kg**	7.7 (5.8-10.1)	2.3 (1.3-4.1)
**2.5-3.5 kg**	1.4 (1.1-1.8)	1.0 (0.7-1.6)
**3.5-4.5 kg**	1 (ref.)	1 (ref.)
**>4.5 kg**	3.1 (1.6-5.9)	1.5 (0.5-5.1)
**Prematurity**		
	12.9 (10.7-15.6)	5.7 (3.3-9.9)
**Low initial heart rate after birth**		
<60 bpm	165 (131-208)	19.6 (11.1-34.9)
60-100 bpm	33.0 (23.3-46.6)	1.8 (1.0-3.2)
100-160 bpm	6.3 (3.5-11.3)	0.3 (0.2-0.8)
>160 bpm	2.4 (0.99-6.0)	0.2 (0.06-0.48)
No need for recording	1 (ref.)	1 (ref.)
**Need for BMV after birth**		
	74.0 (56.1-97.8)	28.7 (16.0-51.2)

*adjustments for fetal heart rate, birth weight category, prematurity, low initial heart rate and need for BMV after birth. In FSB the initial heart rate and presumed need for BMV were set to the lowest category.

Figure text:

Fig 1: Flow chart of participants in a population-based study of births in Haydom hospital during 1.7.2013 to 30.6.2018.

Figure

Click here to access/download;Figure;Lysbilde1.TIF

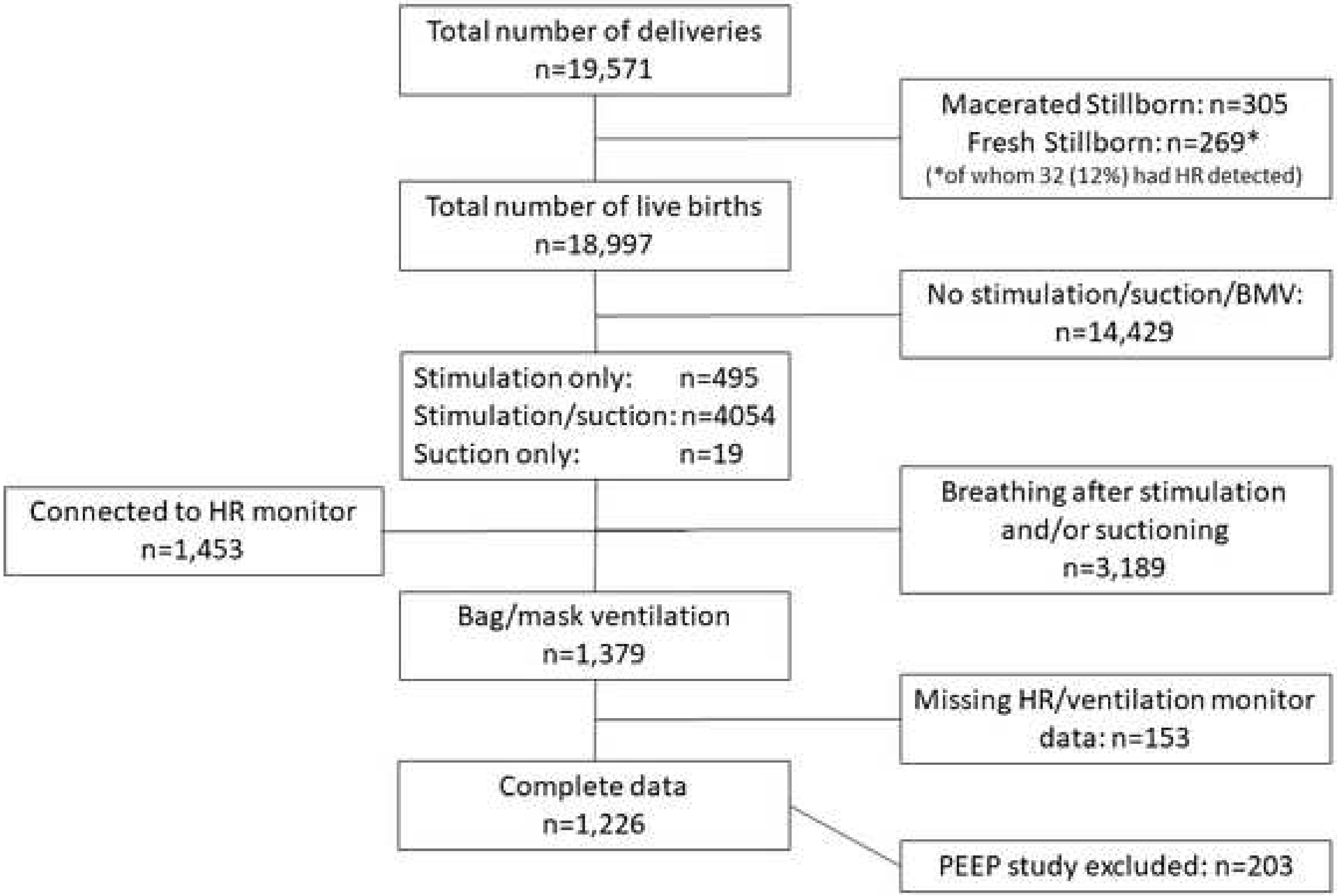

Permission to publish NIMR

Click here to access/download
Supporting Information
Permission to Publish - Hussein Kidanto 29.10.19.pdf

Click here to access/download
Supporting Information
NIMR_Safer Births Approval Certificate_2012.pdf
