# Supplementary material for: Increased perinatal survival and improved ventilation skills over a five-year period: An observational study
Source: PLoS One. 2020 Oct 12;15(10):e0240520. doi: 10.1371/journal.pone.0240520 (PMC7549771; doi:10.1371/journal.pone.0240520)
Supplement: Attachment — Submitted filename: PONE-D-19-30351_R1_reviewer.pdf [file pone.0240520.s003.pdf]

PLOS ONE

Increased perinatal survival and improved ventilation skills over a five-year period: An observational study

--Manuscript Draft--

**Manuscript Number:**	PONE-D-19-30351R1
**Article Type:**	Research Article
**Full Title:**	Increased perinatal survival and improved ventilation skills over a five-year period: An observational study
**Short Title:**	Perinatal survival and ventilation skills
**Corresponding Author:**	Ketil Størdal<br>Norwegian Institute of Public Health<br>Oslo, NORWAY
**Keywords:**	Neonate; newborn; resuscitation; bag-mask ventilation; survival; helping babies breathe; HBB; Perinatal Mortality; Stillbirth; Tanzania
**Abstract:**	Background and aim:<br>The Helping Babies Breathe program gave major reductions in perinatal mortality in Tanzania from 2009 to 2012. We aimed to study whether this effect was sustained, and whether resuscitation skills changed with continued frequent training.<br>Methods:<br>We analysed prospective data covering all births (n=19,571) at Haydom Lutheran Hospital in Tanzania from July 2013 – June 2018. Resuscitation training was continued during this period. All deliveries were monitored by an observer recording the timing of events and resuscitation interventions. Heart rate was recorded by dry-electrode ECG and bag-mask-ventilation by sensors attached to the resuscitator device. We analyzed changes over time in outcomes, use of resuscitation interventions and performance of resuscitation using binary regression models with the log-link function to obtain adjusted relative risks.<br>Results:<br>With introduction of user fees for deliveries since 2014, the number of deliveries decreased by 30% from start to the end of the five-year period. An increase in low heart rate at birth and need for bag-mask-ventilation indicate a gradual selection of more vulnerable newborns delivered in the hospital over time.<br>Despite this selection, newborn deaths <24 hours did not change significantly and was maintained at an average of 8.8/1000 live births. The annual reductions in relative risk for perinatal death adjusted for vulnerability factors was 0.84 (95%CI 0.76-0.94). During the five-year period, longer duration of bag-mask ventilation sequences without interruption was observed. Delivered tidal volumes were increased and mask leak was decreased during ventilation. The time to initiation or total duration of ventilation did not change significantly.<br><br>Conclusion:<br>The reduction in 24-hour newborn mortality after introduction of Helping Babies Breathe was maintained, and a further decrease over the five-year period was evident when analyses were adjusted for vulnerability of the newborns. Perinatal survival and performance of ventilation were significantly improved.
**Order of Authors:**	Ketil Størdal
	Kari Holte
	Joar Eilevstjønn
	Estomih Mduma
	Monica Thallinger
	Jørgen Linde
	Paschal Mdoe
	Hussein Kidanto

Powered by Editorial Manager® and ProduXion Manager® from Aries Systems Corporation

	Hege Langli Ersdal
**Response to Reviewers:**	We would like to thank the editors and reviewers for their work and valuable comments. The editors' remarks have been accommodated. In the resubmitted version, we have incorporated changes according to the comments below, and in the following respond point-by-point to the reviewer's comments.<br><br>Reviewer #1: Thank you for the opportunity to review this paper entitled, "Increased perinatal survival and improved ventilation skills over a five-year period".<br>This prospective analysis evaluates whether the effect of the HBB program was sustained over time.<br>They showed that the decrease 24h newborn mortality was sustained even improved after adjusting for covariables. They also noted an improvement on ventilation performance. The is a wealth of data collected in this project and showing feasible ways of improving neonatal survival is an important goal.<br><br>My major concern is that the is significant overlap in the mortality data with a previous published paper with only 1.5 years of additional data. I think that focusing more on the ventilation portion versus mortality would add more to the literature given that the mortality data on the majority of the mortality data of this data-set has already been published depicting the fact that the effect of the program can be sustained with frequent training.<br><br>The current manuscript adds to the previous paper by ventilation data over time, which have not been published before. In the revised manuscript. We have aimed to further highlight the changes in resuscitation performance stating clearly that this is one of the aims (introduction).<br><br>Abstract: Predictors of survival can be added to the aims as this was a big focus. In the methods, some information regarding training schedule and data analysis can be added to the methods. I think the first 2 sentences in the results regarding the change in population can be condensed. I would remove the last sentence of the conclusion.<br><br>We have included more information in the aim and the methods, and condensed the results to stay within word limits. As the study has several aims, we intend to narrow down and not include predictors of survival in the aim (end of introduction). We have removed the last sentence of the conclusion.<br><br>Introduction: I think adding the already known data regarding frequent training and the effect of HBB from reference 6 would be helpful in the intro.<br><br>We have added one sentence and an additional reference (third paragraph, page 2): "Frequent low-dose training is one of the key elements to improve resuscitation skills.6,7»<br><br>Methods:<br>1. Though the frequency of the skills training is referenced to another paper, I think this paper would benefit from a brief description of the "low dose high frequency skills training" that is a major intervention thought to be responsible for the sustained outcomes. I think the same can be said for the monitoring set up – it is frustrating for me as the reader to have to reference several articles to determine the major determinants of the methods – though these can be referenced some details are required or the paper does not stand alone.<br>Thank you for this suggestion. In the revised manuscript we have included more information regarding the training and monitor set-up as suggested. A figure of the monitor has been added (Fig 2).<br><br>(page 4): Briefly, weekly simulation training was performed on manikins, assisted by local-trainers who had undergone facilitator training for the HBB program. Visiting instructors from other places in Tanzania and Norway also participated regularly.<br><br>(page 5): Physiological data collected from 1st July 2013 until 30 Jun, 2018 included heart rate data recorded from ECG, and ventilation parameters obtained from sensors connected to the ventilation bag using a Newborn Resuscitation Monitor (Laerdal Global Health, Stavanger, Norway) (Fig 1). The arch-shaped ECG sensor was placed

Powered by Editorial Manager® and ProduXion Manager® from Aries Systems Corporation

over the thorax or abdomen of the newborn. Two stainless-steel discs arranged on each side of the flexible arch acted as dry electrodes. The monitor used a hot-wire flow sensor connected to the ventilation bag similar to that in the Florian Respiratory Function Monitor (Acutronic Medical Systems, Hirzel, Switzerland). Volume was calculated by flow signal integration. Pressure was measured using an MPX5010 sensor (Freescale Semiconductor, Austin, TX, USA). The HR was displayed on the monitor installed in front of the resuscitation table visible to the provider, ventilation parameters were not displayed.

2. I would like to know from reading this paper alone the basics of the training and the set-up of the ventilation data collection. In addition I feel that the description of neonatal resuscitation performance was sparse and needed more detail.

Please refer to above corrections for training and monitor setup. Resuscitation performance has been detailed more (page 6, second paragraph).
"The secondary outcomes were clinical performance defined by timing of events during resuscitation: Time from birth to start of BMV, the continuation of BMV by measuring duration of the first ventilation sequence before pause (>5 seconds interruption) and the fraction of time used for BMV once it had been initiated. To assess actual bag-mask ventilation performance, we studied ventilation frequency, tidal volumes and mask leak the first five minutes of bag-mask ventilation."

3. I think that the adjustment for vulnerability factors should go under the statistical analysis section.

We have moved the description of adjustment variables including "vulnerability factors" to the statistics section as suggested.

4. I recommend that the statistical section be reviewed by the statistician involved in the project as this section feels incomplete. For example, the categorical variable comparisons in the table was noted to be chi squared but this is not mentioned in the methods. In addition, your mortality risk ratios I would assume are based off of multi variable logistic regression given that mortality is a binary variable. Please also include the statistical software used.

The method for analysis of categorical date has been added (Page 6, last paragraph). The regression method is not a logistic regression model with logit-link (which provides odds ratios) but as the study is a cohort design we have chosen to use a binary regression model with log-link (which provides relative risks). This information has been added to the text. Description of selection of adjustment variables has been moved and expanded in the revised manuscript, please refer to the manuscript for details.
We have also included the statistical software:
"Data were extracted using Matlab 2019a (MathWorks, Natick, MA) and analyzed using Stata SE 15 (StataCorp. 2017. Stata Statistical Software: Release 15. College Station, TX: StataCorp LP.)»

Results:
1. The first sentence of the second paragraph read awkwardly.

We have simplified the sentence to clarify: "During the observation period, the proportion of deliveries characterized by risk factors for adverse outcome increased."

2. In the second sentence please give exact numbers.

This has been corrected.

3. Did you do/can you add analysis to determine if there was a significant differences between specific time points versus just an overall difference as presented? This may be helpful.

We believe that adding more details would risk to overload the presentation. However, we kindly refer to the paper by Mduma et al (ref. 6) for more details in this respect.

Powered by Editorial Manager® and ProduXion Manager® from Aries Systems Corporation

4. I think graphic depiction of the mortality data versus the many tables would add to the paper.

Thank you for this suggestion. In the revised manuscript the mortality data from Table 2 are replaced by a line diagram (Figure 3).

5. It would be beneficial to break up table 3 as there is so much information. For the linear regression, was the outcome modeled per year? If so the r-square value would be useful in determining the strength of the association. As noted above, the methods in general need more clarification.

We agree that Table 3 (now Table 2 and 3) was large and that breaking up to display bag/mask-ventilation in a separate table provides better readability. For the new Table 2, only two of the analyses are linear, so we chose not to include the R square.

6. Was there a qualitative portion of the respiratory data – ie was BMV indicated when administered and appropriately stopped, were TV given appropriate for the size of the infant, etc

This information would certainly be of interest. However, other studies from the research group have analyzed these data (video analysis of appropriate BMV: Haug et al, Clinical Simulation in Nursing, for Tidal volumes: J.E. Linde et al. Resuscitation 117 (2017) 80–86).

Discussion:
1. Generally the for me it makes sense for strengths and limitations to come right before the conclusion.

This could be done but preferences differ among author, reviewers and editors. We feel the logical flow in the discussion is maintained without this change.

2. I don't think the subheadings are needed

We have removed the subheadings as suggested.

3. I do not think the 3rd paragraph under comparison with other studies adds much to the discussion, and could be removed.

We have removed this sentence as suggested.

4. Can you comment on the fact that continued exposure to higher acuity resuscitations may alone have some impact on these skills.

Thank you for this comment: In a qualitative study from Haydom hospital, the midwives have indicated that feedback from the monitor improves their skills. We have included a new sentence:
"Heart rate feedback during resuscitation may also have improved skills over time, as suggested in a qualitative study from Haydom.7»

5. Can you say that this is the first study that shows the long-term sustained effects if your reference 6 by the same groups shows this as well (acknowledged that yours is an additional 18mo follow-up).

We have modified this sentence, as the current study adds more data and time but the previous study also has long-term data. Instead, we include that the study is the first to monitor changes in ventilation performance with physiological data over time.
"…and provides information on perinatal mortality, resuscitation performance and physiological data during a five-year period"

6. I think it would be helpful in the discussion to add more on the simulation/training aspect with the ventilation outcome with respect to other studies.

We have added new text to several of the paragraphs to highlight the improved resuscitation performance over time, likely due to a high focus on this at the site.

Powered by Editorial Manager® and ProduXion Manager® from Aries Systems Corporation

Reviewer #2: Nicely done paper. I've made comments on the attached file. In brief I had a few questions:

- Can you describe who provided the "low-dose, high-frequency skills training"? Was this done by local personnel, or did it involve visiting instructors from Europe, the USA, etc.?

We have included more information regarding the low-dose, high-frequency skills training in the revised version: Local personnel were trained as HBB-trainers, and some were also facilitated by visiting instructors.
“Briefly, simulation training was performed on manikins, assisted by local-trainers who had undergone facilitator training for the HBB program. Visiting instructors from other places in Tanzania and Norway also participated regularly. The trainings were intended to take place weekly, but with some variations over time.»

- Can you provide data on emergency vs scheduled/repeat C-Section rates over time? This would be helpful in understanding the changing risk profile of your OB population.

We have some information regarding planned vs emergency causes of C-Section over time. However, almost all C-Sections are emergency C-Sections, and the data regarding causes of emergency C-Sections do not provide sufficient quality to answer this question.

- Can you comment on why year 2 was so much worse than other years (Table 4)?

We have also seen this fluctuations over time, with year 2 as an outlier. The resuscitation performance (Table 2 and 3) was relatively similar in year 2. The HBB training varied during the 5-year period, and with shifts in the staff the quality of birth care and post-natal care is likely to have varied though not directly reflected in the data on resuscitation performance. We have added comments on this in the discussion, underlying factors not recorded in our data are likely needed to explain this change.
“The intensity and frequency of HBB training varied during the 5-year period, and due to shifts in the staff the quality of birth care and post-natal care is likely to be vulnerable. The substantial fluctuation over time in perinatal death rates, was however not reflected in the data on resuscitation performance.”

- Do you know if the increased use of BVM was all due to appropriate and indicated use? For example, it appears that incidence of BVM was 4-fold the incidence of 1-min Apgar < 5, & 10-fold the incidence of 5-min Apgar < 5 (Table 3).

In this setting, research from our group has documented that the use of Apgar is highly inaccurate (Ersdal et al, Pediatrics 2012; 129: e1238-1243): 50% of those who died secondary to asphyxia had received a 5-minute Apgar score of ≥7. Thus, the Apgar scores are highly inaccurate and likely to explain this discrepancy. We have included a comment and reference to this finding.
“An increased use of BMV ventilation over the 5-year period reflects the higher morbidity, and Apgar scores in this setting have previously been shown to have low prognostic value.”

- Can you comment on any potential downsides of HBB, for instance, the incidence of Pneumothoraces?

We unfortunately do not have access to portable chest x-rays at the hospital, so pneumothoraces are not possible to confirm accurately. Potentially, the focus on stimulation and suctioning from the start of the HBB program may have resulted in unnecessary suctioning that is now discouraged in resuscitation algorithms. We have added the following:
“However, we cannot rule out that a sustained focus on HBB training may increase unnecessary resuscitation procedures.”

Reviewer #3: Stordal and colleagues present an observational study reporting on perinatal survival and ventilation skills over a five year period of ongoing training

following an initial intervention associated with the Helping Babies Breathe program. Strengths of this study include the prospective nature, the large and relatively complete data obtained, and the inclusion of physiological data to assist in explaining the mortality outcomes. Key limitations of the study include confounding associated with a change in health funding that altered the complexity of neonatal admissions. This confounding required results to undergo statistical adjustment for increasing vulnerability of the infant case mix, to demonstrate an annual reduction in relative risk for perinatal death.
An annotated manuscript is uploaded to assist authors with their revisions. This manuscript makes some suggestions for improved text – but I would strongly recommend the authors to seek professional English editing: overall I think the text length could be reduced by at least 10 % with more efficient word use, and clearer sentence structure.

Thank you for this comment, we have revised the text according to the annotated manuscript and aimed to make the revision more to-the-point. We have also revised the language according to the comments.

MAJOR COMMENTS:
The authors should consult the STROBE guidelines for reporting an observational study.
It would be preferable to make deidentified data available for transparency. De-identified data should not compromise any data privacy restrictions.

Data are available upon specific request. However, we are not allowed to make these openly available due to regulations from the National Institute of Medical Research in Tanzania.
De-identified individual participant data will be made available to researchers whose methodologically sound proposal has been approved by the Scientific Steering Comitee for Safer Births Study Group. Proposals may be submitted up to 36 months following article publication to hege.ersdal@safer.net.

Specific statements regarding Financial Disclosure, Competing Interests and Ethics statements should be entered in line with suggested response types in the online submission form rather than just stating “no” or “as stated in the manuscript”.

This has been corrected.

TITLE: the study design should be indicated in the title and abstract
In the title we have now included: An observational study

ABSTRACT
Background/aim: - the years of the initial program should be studied.
The years of the initial program have been included.

Methods should summarise statistical methodology including method used for adjustment of outcomes. rather than refering loosely to analysis of changes over time… (e.g., prospective observational cross-sectional cohort study).

In the revision we have added “…We analyzed changes over time in outcomes, use of resuscitation interventions and performance of resuscitation using regression models to obtain adjusted relative risks.

Results – please see suggestions for adjusted wording of the last few sentences.

In the revision we have changed the wording of the last few sentences to: “During the five-year period, longer duration of bag-mask ventilation sequences without interruption was observed. Delivered tidal volumes were increased and mask leak was decreased during ventilation.”

Conclusions – please see suggestions for adjusted wording. The final conclusion is not justified without data relating to population level neonatal mortality/morbidity over the same interval (i.e., was not being born in a hospital associated with increased neonatal

Powered by Editorial Manager® and ProduXion Manager® from Aries Systems Corporation

mortality.

We have modified the wording of the conclusion, and removed the last sentence as we do not have population-based data of perinatal mortality for the region.
“The reduction in 24-hour newborn mortality after introduction of Helping Babies Breathe was maintained, and a further decrease over the five-year period was evident when analyses were adjusted for vulnerability of the newborns.”

INTRODUCTION
Overall – the rationale for the study is not well presented. If the HBB decreased mortality, the authors need to justify why a follow-up study was necessary and what it aimed to achieve.
P9, Sentence 1 and 2: It would be helpful to present the fall in global mortality in both 1-5 years and neonatal population to support the statement in the first sentence that reduction in neonatal deaths has been slower over the last 2 decades

The phrasing has been changed according to suggestions. We have added accurate numbers for comparison between neonatal and post-neonatal mortality rates, please refer to the revised manuscript.

P9 final paragraph. This paragraph is unclear. The abstract suggests 1 follow-up study 2013-2018 and previous paragraphs in the introduction suggest the HBB program commenced in 2010 with the initial study extending for 24 months This paragraph suggests a study 2011-2016, and another study with ongoing onsite-low-dose HF HBB. The crossover in dates is confusing to the reader and it is not clear if this is a hybrid of the initial study or a separate follow-up study. I suggest the authors revise the wording of this paragraph to make it explicity clear when the five year study was completed and the timing of the initial study. This paragraph should provide a hypothesis for the study.

We have changed the wording to clarify the periods of time included in our study:
“After introduction of HBB in 2009-2012, efforts to sustain the initial improvements in perinatal mortality continued with onsite low-dose high-frequency HBB training. In the present study, we aimed to document changes in 24-hour neonatal mortality, fresh stillbirths and resuscitation performance during a five-year period from 2013 to 2018.”

METHODS
Overall, the methodology section appeared to include elements of the previous study (2010-2011) and the current 2013-2016 study which was confusing to read. As this paper is about the 2013-2018 study, the methodology should exclusively relate to what was done in this study, with a citation used to reference back to the previous study.
Key elements of the study design and ethical approvals should be provided early in the methodology section.
The setting should simply state the hospital/location and relevant dates (including day not just month and year) for recruitment and data collection. Detail about fees for delivery and the effect on hospital delivery numbers should be part of a discussion.

We have moved the section of ethical approvals to the end of the first paragraph.

Further, we have divided with inclusion of separate headlines the text describing the initial HBB study and the Safer Births study with exact time period and interventions. The text has been revised according to suggestions from the reviewer, please refer to the “track changes” document. The reduction in births and user fees is moved to the discussion.

Participants should be described including eligibility and exclusion criteria

All births in the hospital including stillbirths are recorded in the dataset. This is stated in the revised manuscript: “All births in the hospital from 1st of July 2013 until 30th of June 2018 were recorded in the study.”

Outcomes should be described separately to statistical methodology/treatment, and any explanation/justification of the methodology should go into the discussion.
Statistical elements that are missing include management of missing data, methods to

Powered by Editorial Manager® and ProduXion Manager® from Aries Systems Corporation

	examine subgroups/interactions, how confounding variables were managed, the approach to regression modelling and treatment of collinear variables (e.g., birthweight and gestation).<br><br>The outcomes are described under a separate headline, now also including the secondary outcomes of resuscitation performance.<br>Exposure follows this section, as shown in the revised manuscript ("track changes").<br>We have added adjustment for type of bag used during the study period as explained.<br>In the statistics section we have added information regarding handling of missing data, adjustment for confounding and regression models. Subgroup or stratified analyses could be done, but would add further complexity to the presentation. Therefore we prefer to adjust for subgroup characteristics (for example term/preterm birth). The term "gestational age" appears once in the results section, but the correct term should be "prematurity": Gestational week was not used (this has not been recorded with sufficient precision), only as a dichotomous variable for preterm/term birth. With a Spearman rho of 0.2 between prematurity and birth weight, collinearity does not preclude the analyses.<br><br>RESULTS<br>The exclusion of premature infants from the analysis is not well justified – death in the first 24 hours from prematurity is most often related to respiratory distress and may stem from insufficient resuscitation.<br>We include all – also preterms - in the main analyses. The analysis excluding those that had not been ventilated at all is performed as a sensitivity analysis only, and this is clarified in the revised manuscript. Whether any of these should have been ventilated is possible, however other causes of death including sepsis or severe prematurity are certainly likely in this category. We have modified the wording to clarify, and added the results from this sensitivity analysis to Table 3.<br><br>Some results are presented without accompanying statistics<br>The numbers from statistical analyses have been added to the text where appropriate, in addition to the original version displaying this in Tables.<br><br>It is not clear why infants involved in the PEEP trial were excluded from the ventilation analysis but not from the mortality outcomes, given PEEP may alter effectiveness of resuscitation and hence the resulting mortality.<br><br>Thank you for this important comment. The type of bag used did not predict mortality (p=0.25) and therefore did not satisfy the criteria for inclusion in the adjusted model. This is now explained in the Methods/statistics section.<br><br>Ventilation frequency should be presented as inflations/min or breaths/min not as 60/min<br>Tidal volume was corrected for weight and this should be clear in the results.<br><br>We have changed this in the revised manuscript.<br><br>DISCUSSION<br>Paragraph 1: suggest that a full stop is provided after "user fees" and that the rest of the sentence becomes a new sentence as "Consequently, hospitalised newborns included a higher proportion of infants with increased vulnerability for adverse outcomes".<br><br>We have revised this paragraph according to suggestions.<br><br>Reference to the previous study would be better phrased as "before and after initial implementation of the HBB program was 11.1/1000 and 7.2/1000 respectively. The following sentence could be revised to indicate that reduction in early neonatal mortality accounted for the majority of the overall decrease in baseline perinatal mortality from 27/1000 to 21.7/1000 over the same period.<br>Thank you for this suggestion, we have rephrased and added the number showing that decrease in ENM accounted for most of the reduction in perinatal mortality:<br>"The baseline perinatal mortality was 27/1000 and fell to 21.7/1000, and the decrease in ENM thus accounted for 74% of the reduced perinatal mortality"

Powered by Editorial Manager® and ProduXion Manager® from Aries Systems Corporation

	It is not clear why the authors say that the period of observation started 2 years after regular HBB implementation and extended for a further 1.5 years, when the actual study went for 5 years. The numbers don't add up?<br><br>We have clarified in the revised manuscript, stating time periods clearly in the Methods section and highlighting what is new in the present study.<br>"In the present follow-up, our period of observation starts two years after regular HBB implementation and provides information on perinatal mortality and resuscitation performance during a five-year period"<br><br>When other studies are quoted, the discussion would have more impact if the information was synthesised and presented as a comparison to the current data to indicate what the literature is telling us overall about the issue at hand. Reference to the study from Nepal does not indicate what the intervention was that led to a reduction of deaths from intra-partum complications but didn't change overall neonatal mortality, and hence the reader is left confused about the relevance of this information.<br><br>We have added to the discussion points that we believe are relevant to explain why HBB implementation have different impact across countries (page 12 and 13).<br><br>It was not clear that the increase in (weight corrected) tidal volumes was due to the HBB training or due to the change in patient mix. It is odd that the increase in tidal volume during resuscitation was not matched by an increase in heart rate. How do the authors explain this?<br><br>The data shown for HR in Table 2 are absolute numbers of the first recorded heart rate and during the first 60 seconds thereafter in order to characterize vulnerability of the newborns over time. Thus, the HR shown is not after one minute of BMV, which is now stated more clearly in the revised table 2.<br>"Mean heart rate during 0-60 seconds after initial recording, median (IQR)»<br><br>MINOR COMMENTS<br>Numbers < 10 should be written in full<br>This has been corrected.<br><br>Overall, there are too many unnecessary abbreviations in the manuscript that make it difficult to read and digest.<br><br>We have replaced FSB and MSB with the phrases "fresh stillbirth" and "macerated stillbirths" throughout.<br><br>Table 1 – data are usually presented as n (%) rather than % (n). As it is not clear what the numbers are for the child characteristics weight ranges it would be best to indicate in the table legend at the bottom of the table that data are presented as % (n) unless indicated otherwise. Any abbreviations (e.g., bpm) need to be explained in the table legend (which goes for other tables also – e.g., MSB in Table 2).<br><br>We have changed the tables according to this comment to indicate % (n). The legend includes any abbreviation, and MSB is not used now.<br><br>Table 5 needs to indicate what the numbers are – presume relative risk and 95 % CI?<br><br>Correct, Table 5 (now Table 4) is relative risk and 95% confidence interval as now included in the headline.
**Additional Information:**	
**Question**	**Response**
**Financial Disclosure**<br><br>Enter a financial disclosure statement that describes the sources of funding for the	No

work included in this submission. Review the submission guidelines for detailed requirements. View published research articles from *PLOS ONE* for specific examples.<br><br>This statement is required for submission and **will appear in the published article** if the submission is accepted. Please make sure it is accurate.<br><br>**Unfunded studies**<br>Enter: *The author(s) received no specific funding for this work.*<br><br>**Funded studies**<br>Enter a statement with the following details:<br>• Initials of the authors who received each award<br>• Grant numbers awarded to each author<br>• The full name of each funder<br>• URL of each funder website<br>• Did the sponsors or funders play any role in the study design, data collection and analysis, decision to publish, or preparation of the manuscript?<br>• **NO** - Include this sentence at the end of your statement: *The funders had no role in study design, data collection and analysis, decision to publish, or preparation of the manuscript.*<br>• **YES** - Specify the role(s) played.<br><br>* typeset	
**Competing Interests**<br><br>Use the instructions below to enter a competing interest statement for this submission. On behalf of all authors, disclose any competing interests that could be perceived to bias this work—acknowledging all financial support and any other relevant financial or non-financial competing interests.<br><br>This statement **will appear in the published article** if the submission is accepted. Please make sure it is accurate. View published research articles from *PLOS ONE* for specific examples.	No

**NO authors have competing interests**<br><br>Enter: *The authors have declared that no competing interests exist.*<br><br>**Authors with competing interests**<br><br>Enter competing interest details beginning with this statement:<br><br>*I have read the journal's policy and the authors of this manuscript have the following competing interests: [insert competing interests here]*<br><br>* typeset	
**Ethics Statement**<br><br>Enter an ethics statement for this submission. This statement is required if the study involved:<br><br>• Human participants<br>• Human specimens or tissue<br>• Vertebrate animals or cephalopods<br>• Vertebrate embryos or tissues<br>• Field research<br><br>Write "N/A" if the submission does not require an ethics statement.<br><br>General guidance is provided below. Consult the submission guidelines for detailed instructions. **Make sure that all information entered here is included in the Methods section of the manuscript.**	This study was approved by the National Institute for Medical Research, Tanzania (NIMR/HQ/R8a/Vol. IX/1434) and the Regional Committee for Medical and Health Research Ethics, Western Norway (2013/110/REK). All health care providers gave informed oral consent. The mothers were informed but formal consent was not obtained for this observational study due to the urgency of mothers arriving in late stages of labor. This consent procedure was approved by the ethical committees. Uploaded is a letter of permission from the National Institute of Medical Research, Tanzania.

Powered by Editorial Manager® and ProduXion Manager® from Aries Systems Corporation

**Format for specific study types**<br><br>**Human Subject Research (involving human participants and/or tissue)**<br>• Give the name of the institutional review board or ethics committee that approved the study<br>• Include the approval number and/or a statement indicating approval of this research<br>• Indicate the form of consent obtained (written/oral) or the reason that consent was not obtained (e.g. the data were analyzed anonymously)<br><br>**Animal Research (involving vertebrate animals, embryos or tissues)**<br>• Provide the name of the Institutional Animal Care and Use Committee (IACUC) or other relevant ethics board that reviewed the study protocol, and indicate whether they approved this research or granted a formal waiver of ethical approval<br>• Include an approval number if one was obtained<br>• If the study involved *non-human primates*, add *additional details* about animal welfare and steps taken to ameliorate suffering<br>• If anesthesia, euthanasia, or any kind of animal sacrifice is part of the study, include briefly which substances and/or methods were applied<br><br>**Field Research**<br><br>Include the following details if this study involves the collection of plant, animal, or other materials from a natural setting:<br>• Field permit number<br>• Name of the institution or relevant body that granted permission	
**Data Availability**<br><br>Authors are required to make all data underlying the findings described fully available, without restriction, and from the time of publication. PLOS allows rare exceptions to address legal and ethical concerns. See the PLOS Data Policy and FAQ for detailed information.	No - some restrictions will apply

A Data Availability Statement describing where the data can be found is required at submission. Your answers to this question constitute the Data Availability Statement and **will be published in the article**, if accepted.<br><br>**Important:** Stating 'data available on request from the author' is not sufficient. If your data are only available upon request, select 'No' for the first question and explain your exceptional situation in the text box.<br><br>Do the authors confirm that all data underlying the findings described in their manuscript are fully available without restriction?	
**Describe where the data may be found in full sentences. If you are copying our sample text, replace any instances of XXX with the appropriate details.**<br><br>• If the data are **held or will be held in a public repository**, include URLs, accession numbers or DOIs. If this information will only be available after acceptance, indicate this by ticking the box below. For example: *All XXX files are available from the XXX database (accession number(s) XXX, XXX.).*<br>• If the data are all contained **within the manuscript and/or Supporting Information files**, enter the following: *All relevant data are within the manuscript and its Supporting Information files.*<br>• If neither of these applies but you are able to provide **details of access elsewhere**, with or without limitations, please do so. For example:<br><br>*Data cannot be shared publicly because of [XXX]. Data are available from the XXX Institutional Data Access / Ethics Committee (contact via XXX) for researchers who meet the criteria for access to confidential data.*<br><br>*The data underlying the results presented in the study are available from (include the name of the third party*	Data are available upon specific request. However, we are not allowed to make these openly available due to regulations from the National Institute of Medical Research in Tanzania.<br>De-identified individual participant data will be made available to researchers whose methodologically sound proposal has been approved by the Scientific Steering Comitee for Safer Births Study Group. Proposals may be submitted to post@safer.net.

and contact information or URL).<br>• This text is appropriate if the data are owned by a third party and authors do not have permission to share the data.<br><br>* typeset	
Additional data availability information:	

Manuscript Click here to access/download;Manuscript;Manuscript.docx

Increased perinatal survival and improved ventilation skills over a five-year period: An observational study

Ketil Størdal[1,2], Joar Eilevstjønn[3], Estomih Mduma[4], Kari Holte[1], Monica Thallinger[5], Jørgen Linde[6], Paschal Mdoe[4], Hussein Kidanto[7], Hege Ersdal[6,8].

[1] Ostfold Hospital Trust, Grålum, Norway.

[2] Norwegian Institute of Public Health, Oslo, Norway.

[3] Laerdal Medical AS, Stavanger, Norway.

[4] Haydom Lutheran Hospital, Manyara, Tanzania.

[5] Vestre Viken HF, Gjettum, Norway.

[6] Helse Stavanger HF, Stavanger, Norway.

[7] Muhimbili National Hospital, Dar Es Salam, Tanzania.

[8] SAFER, Stavanger, Norway.

Contact author: Ketil Størdal, Division for Mental and Physical Health, Norwegian Institute of Public Health, PO Box 222 Skøyen, 0213 Oslo, Norway. Phone 0047 48272337, ketil.stordal@fhi.no

Abstract

Background and aim:

The Helping Babies Breathe program gave major reductions in perinatal mortality in Tanzania from 2009 to 2012. We aimed to study whether this effect was sustained, and whether resuscitation skills changed with continued frequent training.

Methods:

We analysed prospective data covering all births (n=19,571) at Haydom Lutheran Hospital in Tanzania from July 2013 – June 2018. Resuscitation training was continued during this period. All deliveries were monitored by an observer recording the timing of events and resuscitation interventions. Heart rate was recorded by dry-electrode ECG and bag-mask-ventilation by sensors attached to the resuscitator device. We analyzed changes over time in outcomes, use of resuscitation interventions and performance of resuscitation using binary regression models with the log-link function to obtain adjusted relative risks.

Results:

With introduction of user fees for deliveries since 2014, the number of deliveries decreased by 30% from start to the end of the five-year period. An increase in low heart rate at birth and need for bag-mask-ventilation indicate a gradual selection of more vulnerable newborns delivered in the hospital over time.

Despite this selection, newborn deaths <24 hours did not change significantly and was maintained at an average of 8.8/1000 live births. The annual reductions in relative risk for perinatal death adjusted for vulnerability factors was 0.84 (95%CI 0.76-0.94).

During the five-year period, longer duration of bag-mask ventilation sequences without interruption was observed. Delivered tidal volumes were increased and mask leak was decreased during ventilation. The time to initiation or total duration of ventilation did not change significantly.

Conclusion:

The reduction in 24-hour newborn mortality after introduction of Helping Babies Breathe was maintained, and a further decrease over the five-year period was evident when analyses were adjusted for vulnerability of the newborns. Perinatal survival and performance of ventilation were significantly improved.

Introduction

The reduction of neonatal deaths over the last decade has been modest, despite declining mortality of infants between 1 month and five years of age.[1] The estimated global neonatal mortality (≤ 28 days) fell from 31/1000 in 2000 to 18/1000 in 2017.[2] The annual rate reduction in neonatal mortality was 3.1% compared to 4.7% after the neonatal period. Consequently, the relative contribution of neonatal deaths in the global under-5-mortality has increased from 39% to 45%.[1] The majority of these neonatal deaths are preventable. Safer births are achievable by improvements in a continuum from pregnancy, obstetric to neonatal care, each accounting for 1/3 of preventable deaths.[3]

Intrapartum-related events, formerly referred to as birth asphyxia and defined as inadequate onset of spontaneous breathing after birth, are one of the three most prevalent causes of under-5-mortality.[1] The Helping Babies Breathe (HBB) program aimed to reduce death from intrapartum events in low- and middle-income settings, through training of health care workers in early identification and treatment.[4]

In Tanzania, the roll-out of HBB from 2010 was associated with a 47% reduction in fresh stillbirths and early neonatal mortality over 24 months in eight large birth facilities.[5] Haydom Lutheran Hospital was one of the eight sites participating in the early roll-out of HBB. HBB training and research related to safer deliveries and newborn resuscitations continued in the Safer Births program.[6] Frequent low-dose training is one of the key elements to improve resuscitation skills.[6,7]

In Tanzania neonatal mortality decreased from 33/1000 in 2000 to 21/1000 in 2017.[2] However, vital birth statistics are not available,[8,9] and estimates are inaccurate as almost half of the deliveries in Tanzania take place outside birth facilities. In a rural area with a low proportion of deliveries in hospital, this selection may preclude interpretation of temporal trends in mortality rates. It is therefore pertinent to adjust for vulnerability background factors in hospital deliveries.

After introduction of HBB in 2009-2012, efforts to sustain the initial improvements in perinatal mortality continued with onsite low-dose high-frequency HBB training. In the present study, we aimed to document changes in 24-hour neonatal mortality, fresh stillbirths and resuscitation performance during a five-year period from 2013 to 2018.

Methods

Haydom Lutheran Hospital is situated in the rural area of the Manyara region of Northern Tanzania. The hospital has a catchment area of over 2 million inhabitants, and is the only hospital in the region providing comprehensive obstetric care with 250-400 deliveries per month. Haydom is a non-governmental hospital managed by the Lutheran church of Tanzania. All births in the hospital from 1st of July 2013 until 30th of June 2018 were recorded in the study.

This study was approved by the National Institute for Medical Research, Tanzania (NIMR/HQ/R8a/Vol. IX/1434) and the Regional Committee for Medical and Health Research Ethics, Western Norway (2013/110/REK). All health care providers gave informed oral consent. The mothers were informed but formal consent was not obtained for this observational study due to the urgency of mothers arriving in late stages of labor. This consent procedure was approved by the ethical committees.

The initial HBB implementation

The local procedure for newborn resuscitation followed the HBB guidelines as implemented locally from 2009-2012[5], emphasizing stimulation and early initiation of bag-mask ventilation (BMV).[4] Newborn resuscitation was mainly the responsibility of midwives. Six labour rooms were each equipped with a resuscitation table. After caesarean section, newborn resuscitation was performed in a room adjacent to the operating theatre. Midwives determined if the newborn remained with the mother or was transferred to a neonatal ward after resuscitation. The neonatal ward provided basic care: respiratory support was limited to provision of supplemental oxygen with nasal prongs.

The Safer Births program

The Safer Births program, starting in 2013, aimed to further facilitate HBB training and improve obstetric and newborn care by low-cost technology.[6,10] During the study all midwives were encouraged to low-dose, high-frequency skills training as described by Mduma et al.[11] Briefly, simulation training was performed on manikins, assisted by local-trainers who had undergone facilitator training for the HBB program. Visiting instructors from other places in Tanzania and Norway also participated regularly. The trainings were intended to take place weekly, but with some variations over time.

Trained data collectors observed and recorded the timing of events immediately after birth, and vital data and outcomes from all deliveries were recorded in a database.

Physiological data collected from 1st July 2013 until 30 Jun, 2018 included heart rate data recorded from ECG, and ventilation parameters obtained from sensors connected to the ventilation bag using a Newborn Resuscitation Monitor (Laerdal Global Health, Stavanger, Norway) (Fig 1). The arch-shaped ECG sensor was placed over the thorax or abdomen of the newborn. Two stainless-steel discs arranged on each side of the flexible arch acted as dry electrodes. The monitor used a hot-wire flow sensor connected to the ventilation bag similar to that in the Florian Respiratory Function Monitor (Acutronic Medical Systems, Hirzel, Switzerland). Volume was calculated by flow signal integration. Pressure was measured using an MPX5010 sensor (Freescale Semiconductor, Austin, TX, USA). The HR was displayed on the monitor installed in front of the resuscitation table visible to the provider, ventilation parameters were not displayed. [12] We analysed data for a five years period with collection of physiological data until 30th of June 2018 (Fig 2, flow chart).

Main and secondary outcomes

The main outcome was early neonatal mortality (ENM) defined as death before 24 hours of life among newborns delivered alive at Haydom Lutheran Hospital. As shown previously, live born babies with severe asphyxia may be misclassified as fresh stillbirths.[13,14] Therefore, we also used the combined outcome of ENM and fresh stillbirths to analyze perinatal mortality.

The secondary outcomes were clinical performance defined by timing of events during resuscitation: Time from birth to start of BMV, the continuation of BMV by measuring duration of the first ventilation sequence before pause (>5 seconds interruption) and the fraction of time used for BMV once it had been initiated. To assess actual bag-mask ventilation performance, we studied ventilation frequency, tidal volumes and mask leak the first five minutes of bag-mask ventilation.

Main exposure and covariates

We analyzed by year (1st of July – 30th of June, starting 1st of July 2013) for the five-year period to assess effects of ongoing training in newborn resuscitation over time.

To assess for potential confounding, we obtained information from the prospective database of pregnancy complications, birth weight, prematurity, fetal heart rate abnormalities, the last recorded fetal heart rate and the need for BMV at birth and type of resuscitator used.

Statistical methods

Descriptive analyses were performed by calculation of medians with interquartile ranges (IQR) or means with standard deviations according to their distribution. Categorical variables were analyzed using percentages and Chi-square test.

We used binary regression with log-link function to estimate relative risks for ENM by time. [13,15] Risk factors which changed over time and were associated with the outcome ($p<0.10$) were included in a multivariate regression model if they changed the relative risk estimates by >10%. In the final model we therefore adjusted for birth weight, prematurity, fetal heart rate abnormalities, last recorded fetal heart rate and the need for BMV at birth. Cases with missing variables for any of the included adjustment variables (n=15) were excluded from the adjusted model.

We also performed sensitivity analyses excluding newborns who died without a history of resuscitation at birth because conditions other than intra-partum hypoxia may cause ENM.

Data were extracted using Matlab 2019a (MathWorks, Natick, MA) and analyzed using Stata SE 15 (StataCorp. 2017. Stata Statistical Software: Release 15. College Station, TX: StataCorp LP.)

Results

During the five-year period, 18,997 infants were born alive, with a reduction over time from 4,662 to 3,197 annual births (Table 1). ENM fluctuated around 8.8/1000 live births, and the combined outcome of perinatal mortality (fresh stillbirths and ENM) around 22.6/1000 with no significant differences over time (Fig 3).

Vulnerability factors over time

During the observation period, the proportion of deliveries characterized by risk factors for adverse outcome increased. The proportion of pregnancies with antenatal problems including preeclampsia increased significantly from 0.91% to 2.1% (p<0.001). The mean birth weight increased slightly, but an increasing proportion were <1500 g and preterm births increased from 2.5% to 3.5% (Table 1).

Abnormal fetal heart rate during delivery increased gradually from 3% to 9%. The first detected heart rate among resuscitated babies fell from a median of 142 to 108 beats per minute (bpm), and the proportion with heart rate <100 bpm at birth increased from 8% to 22% (p<0.001, Table 2).

Mortality adjusted for vulnerability

In unadjusted analyses, the ENM increased slightly during the five-year period (relative risk per year 1.11, 95%CI 1.00-1.24, Table 3). Vulnerability factors all increased during this period, as shown in Table 1 and 2. After adjustment for abnormal fetal heart rate during delivery, birth weight, prematurity, initial heart rate and need for BMV at birth, there was no significant reduction in relative risk for ENM (relative risk per year 0.95, 95%CI 0.84-1.07, Table 3). Death the first 24 hours could be due to other reasons than intrapartum events, especially prematurity. Of those who died, 55/169 (33%) had not been ventilated, and thus would likely not be diagnosed as asphyxiated. We performed a sensitivity analysis without these 55: The unadjusted and adjusted analyses were essentially unchanged (Table 3).

The perinatal mortality showed no appreciable change during the five-year period in unadjusted analyses. However, after adjustment for vulnerability factors we found a significant annual decrease of 16% in perinatal mortality (relative risk per year 0.84, 95%CI 0.76-0.94, Table 3).

In the fully adjusted model, year of birth, fetal heart rate abnormalities, initial heart rate, birth weight, prematurity and need for BMV predicted perinatal death (Table 4).

Changes in resuscitation performance

The proportion who received BMV varied from 6.1-7.7%, but increased to 8.8% the last year (Table 2). Time from birth to start of BMV was lowest the last year (median 99 seconds) but did not change significantly over time (p=0.76, Table 5). The duration of the first BMV sequence increased over time from a median of 11 to 32 seconds (p<0.001, Table 5). Once initiated, BMV was continued for an increasing part of the first 60 seconds, from a median of 24 seconds the first compared to 48 seconds the last year (p<0.001, Table 5). The duration of ventilation from the first to the last inflation remained stable (Table 5).

Median ventilation frequency decreased from >60 breaths/min the first year to 44-48 breaths/min the last three years of observation (p<0.001). The median weight-adjusted tidal volume, mean and peak airway pressures increased (p<0.001), whereas mask leak decreased slightly but significantly over time (p=0.001, Table 5).

Discussion

During a five-year period following a successful implementation of frequent HBB training, we found no further significant decline in ENM. A worrying decrease in hospital deliveries was observed, likely due to introduction of user fees since 2014 of 12 and 30 USD for vaginal and caesarean section deliveries, respectively. Consequently, hospitalized newborns included a higher proportion of infants with increased vulnerability for adverse outcomes. In analyses adjusted for vulnerability factors, perinatal deaths decreased significantly during the five years.

Among several strengths in the study is the large size population-based study, performed in a rural setting of a low-income country with high burden of disease. The prospective design ensured high-quality data from the delivery and immediate post-natal period. Collection of physiological data during the first minutes of life provides information which is rarely accessible.

The selection of higher-risk deliveries during the period is evident, and accounted for as far as possible in the adjusted analysis. However, as in any observational study, residual confounding cannot be completely ruled out. A population-based birth registry with inclusion of all pregnancies including home deliveries would capture the complete picture and avoid selection bias. Specific causes of ENM were not possible to identify in our study, though in a sub-study from this cohort half of the deaths were related to intra-partum events.[16] We were, however, able to exclude deaths unlikely to be caused by perinatal hypoxia in a sensitivity analysis. An increased use of BMV ventilation over the 5-year period likely reflects the higher morbidity, and Apgar scores in this setting have previously been shown to have low prognostic value.[17] However, we cannot rule out that a sustained focus on HBB training may increase unnecessary resuscitation procedures.

During the nation-wide HBB roll-out during 2010/2011 in Tanzania, ENM in eight large hospitals including Haydom decreased from 13.4 to 7.1/1000. Fresh stillbirths decreased from 19 to 14.4/1000, and a remarkable fall of perinatal mortality of 34% was reported.[5] Before and after initial implementation of the HBB program ENM at Haydom was 11.1/1000 and 7.2/1000 respectively. The baseline perinatal mortality was 27/1000 and fell to 21.7/1000, and the decrease in ENM thus accounted for 74% of the reduced perinatal mortality.[18] In the present follow-up, our period of observation starts two years after regular HBB implementation and provides information on perinatal mortality, resuscitation performance and physiological data during a five-year period [6]. Our study shows that the initial reduction in perinatal mortality seen after HBB implementation has continued.

HBB has been introduced globally, and overall a reduction in perinatal mortality has been reported.[19] The variation between countries with highly successful implementation [5,20,21] to sites reporting no or modest effect on mortality [22,23] is of note. The relative contribution of intra-partum hypoxia to ENM across countries may explain some differences, because a lower effect would be expected when other causes of ENM dominate. Besides, implementation and uptake of HBB could influence the results, and repeated low-dose onsite training is likely to facilitate successful implementation.[11] Several studies have reported improved resuscitation knowledge and practical skills after HBB training in a simulation setting.[4,24] Some loss of performance over time is observed,[25-27] but skills retention was improved by frequent re-training and use of self-evaluation checklists.[26] Importantly, changes in clinical management do not necessarily follow simulated performance.[28] The present study documents improved resuscitation skills that are sustained over time, and is likely to improve perinatal mortality. Heart rate feedback during resuscitation may also have improved skills over time, as suggested in a qualitative study from Haydom.[7]

In a study from Nepal, a reduction in the percentage of deaths attributable to intra-partum related complications from 51 to 33% was observed, but overall newborn mortality was unchanged.[29] This was also reported in the recent meta-analysis, pointing to the importance of a package of interventions needed to address the main causes of neonatal death.[19] In our study, one third of deaths occurred in newborns that did not receive BMV after birth. Intra-partum events accounted for about half the deaths before 7 days age at Haydom during 2014-17, but preterm birth and sepsis were among other preventable causes of death.[16]

Our study is the first to report long-term changes in performance of resuscitation and BMV after HBB implementation. Analyses of actual ventilation performance demonstrate that practical skills improved over time with significantly longer duration of the first ventilation sequence and reduction in ventilation frequency targeting the recommended range. The increase in more complex patients may also change the resuscitation practice over time. The reduction in mask leak over time was desirable though admittedly small. Higher tidal volumes are associated with more rapid HR increase in observational studies from Haydom,[30,31] but should be further studied in controlled trials before we can conclude whether this is recommendable.

In the initial HBB roll-out at Haydom, the use of suction/stimulation increased whereas use of BMV decreased significantly.[11] We found a further increase in suction/stimulation but also of BMV, which could be expected with increased vulnerability over time. In a meta-analysis of the effect of HBB training on resuscitation practices from four studies, the overall use of suctioning, stimulation or BMV was unchanged. However, interventions during the first Golden minute increased 2.5-fold.[32] In our study the increase during the five years of time used to ventilate the first minute after BMV was started is of note. The intensity and frequency of HBB training varied during the 5-year period, and due to shifts in the staff the

quality of birth care and post-natal care is likely to be vulnerable. The substantial fluctuation over time in perinatal death rates, was however not reflected in the data on resuscitation performance.

In the present study the quality of immediate newborn care at birth was improved over time, probably due to continuous focus on frequent HBB training. However, coverage and access are likely even more important for the total perinatal mortality than quality of care in the hospital. These factors are interrelated, as increased quality in a delivery unit is likely to encourage more pregnant women to seek care. However, financial constraints may counteract this effect. In Tanzania in 2011/12, 44% had less than 1.25 USD per day for their daily living, and poverty was even more prevalent in rural districts.[33] In this context, 12 to 30 USD in hospital fees for delivery is a considerable burden to the family economy. Free delivery care should be a high priority, as provided in governmental hospitals in Tanzania.

In this study we have shown that neonatal resuscitation skills improved over time at a site with high focus on continued and frequent HBB training. The perinatal mortality adjusted for vulnerability factors decreased, whereas ENM showed a non-significant decline. Further reduction in neonatal mortality in Tanzania will likely require a comprehensive approach to increase facility births and address the major causes of death.

1. Liu L, Oza S, Hogan D, et al. Global, regional, and national causes of under-5 mortality in 2000-15: an updated systematic analysis with implications for the Sustainable Development Goals. *Lancet.* 2016;388(10063):3027-3035.
2. Hug L, Alexander M, You D, Alkema L, Estimation UNI-aGfCM. National, regional, and global levels and trends in neonatal mortality between 1990 and 2017, with scenario-based projections to 2030: a systematic analysis. *Lancet Glob Health.* 2019;7(6):e710-e720.
3. Bhutta ZA, Das JK, Bahl R, et al. Can available interventions end preventable deaths in mothers, newborn babies, and stillbirths, and at what cost? *Lancet.* 2014;384(9940):347-370.
4. Singhal N, Lockyer J, Fidler H, et al. Helping Babies Breathe: global neonatal resuscitation program development and formative educational evaluation. *Resuscitation.* 2012;83(1):90-96.
5. Msemo G, Massawe A, Mmbando D, et al. Newborn mortality and fresh stillbirth rates in Tanzania after helping babies breathe training. *Pediatrics.* 2013;131(2):e353-360.
6. Mduma E, Kvaloy JT, Soreide E, et al. Frequent refresher training on newborn resuscitation and potential impact on perinatal outcome over time in a rural Tanzanian hospital: an observational study. *BMJ Open.* 2019;9(9):e030572.
7. Moshiro R, Ersdal HL, Mdoe P, Kidanto HL, Mbekenga C. Factors affecting effective ventilation during newborn resuscitation: a qualitative study among midwives in rural Tanzania. *Glob Health Action.* 2018;11(1):1423862.
8. Rebnord T, Ostbye T, Mmbaga BT, McHome B, Lie RT, Daltveit AK. Time trends in management of HIV-positive pregnant women in Northern Tanzania: A registry-based study. *PLoS One.* 2017;12(9):e0184362.
9. Bergsjo P, Mlay J, Lie RT, Lie-Nielsen E, Shao JF. A medical birth registry at Kilimanjaro Christian Medical Centre. *East Afr J Public Health.* 2007;4(1):1-4.
10. http://www.saferbirths.com/. Accessed 19.06.2020.
11. Mduma E, Ersdal H, Svensen E, Kidanto H, Auestad B, Perlman J. Frequent brief on-site simulation training and reduction in 24-h neonatal mortality--an educational intervention study. *Resuscitation.* 2015;93:1-7.
12. Linde JE, Schulz J, Perlman JM, et al. Normal Newborn Heart Rate in the First Five Minutes of Life Assessed by Dry-Electrode Electrocardiography. *Neonatology.* 2016;110(3):231-237.
13. Thallinger M, Ersdal HL, Francis F, et al. Born not breathing: A randomised trial comparing two self-inflating bag-masks during newborn resuscitation in Tanzania. *Resuscitation.* 2017;116:66-72.
14. Ersdal HL, Eilevstjonn J, Linde JE, et al. Fresh stillborn and severely asphyxiated neonates share a common hypoxic-ischemic pathway. *Int J Gynaecol Obstet.* 2017.
15. Thallinger M, Ersdal HL, Morley C, et al. Neonatal ventilation with a manikin model and two novel PEEP valves without an external gas source. *Arch Dis Child Fetal Neonatal Ed.* 2017;102(3):F208-F213.
16. Moshiro R, Perlman JM, Mdoe P, Kidanto H, Kvaloy JT, Ersdal HL. Potential causes of early death among admitted newborns in a rural Tanzanian hospital. *PLoS One.* 2019;14(10):e0222935.
17. Ersdal HL, Mduma E, Svensen E, Perlman J. Birth asphyxia: a major cause of early neonatal mortality in a Tanzanian rural hospital. *Pediatrics.* 2012;129(5):e1238-1243.
18. Mduma ER, Ersdal H, Kvaloy JT, et al. Using statistical process control methods to trace small changes in perinatal mortality after a training program in a low-resource setting. *Int J Qual Health Care.* 2018;30(4):271-275.
19. Versantvoort JMD, Kleinhout MY, Ockhuijsen HDL, Bloemenkamp K, de Vries WB, van den Hoogen A. Helping Babies Breathe and its effects on intrapartum-related stillbirths and neonatal mortality in low-resource settings: a systematic review. *Arch Dis Child.* 2019.
20. Kc A, Wrammert J, Clark RB, et al. Reducing Perinatal Mortality in Nepal Using Helping Babies Breathe. *Pediatrics.* 2016;137(6).

21. Arabi AME, Ibrahim SA, Manar AR, et al. Perinatal outcomes following Helping Babies Breathe training and regular peer-peer skills practice among village midwives in Sudan. *Arch Dis Child.* 2018;103(1):24-27.
22. Bellad RM, Bang A, Carlo WA, et al. A pre-post study of a multi-country scale up of resuscitation training of facility birth attendants: does Helping Babies Breathe training save lives? *BMC Pregnancy Childbirth.* 2016;16(1):222.
23. Goudar SS, Somannavar MS, Clark R, et al. Stillbirth and newborn mortality in India after helping babies breathe training. *Pediatrics.* 2013;131(2):e344-352.
24. Musafili A, Essen B, Baribwira C, Rukundo A, Persson LA. Evaluating Helping Babies Breathe: training for healthcare workers at hospitals in Rwanda. *Acta Paediatr.* 2013;102(1):e34-38.
25. Bang A, Patel A, Bellad R, et al. Helping Babies Breathe (HBB) training: What happens to knowledge and skills over time? *BMC Pregnancy Childbirth.* 2016;16(1):364.
26. Kc A, Wrammert J, Nelin V, et al. Evaluation of Helping Babies Breathe Quality Improvement Cycle (HBB-QIC) on retention of neonatal resuscitation skills six months after training in Nepal. *BMC Pediatr.* 2017;17(1):103.
27. Arlington L, Kairuki AK, Isangula KG, et al. Implementation of "Helping Babies Breathe": A 3-Year Experience in Tanzania. *Pediatrics.* 2017;139(5).
28. Ersdal HL, Vossius C, Bayo E, et al. A one-day "Helping Babies Breathe" course improves simulated performance but not clinical management of neonates. *Resuscitation.* 2013;84(10):1422-1427.
29. Wrammert J, Kc A, Ewald U, Malqvist M. Improved postnatal care is needed to maintain gains in neonatal survival after the implementation of the Helping Babies Breathe initiative. *Acta Paediatr.* 2017;106(8):1280-1285.
30. Moshiro R, Perlman JM, Kidanto H, Kvaloy JT, Mdoe P, Ersdal HL. Predictors of death including quality of positive pressure ventilation during newborn resuscitation and the relationship to outcome at seven days in a rural Tanzanian hospital. *PLoS One.* 2018;13(8):e0202641.
31. Linde JE, Schulz J, Perlman JM, et al. The relation between given volume and heart rate during newborn resuscitation. *Resuscitation.* 2017;117:80-86.
32. Budhathoki SS, Gurung R, Ewald U, Thapa J, Kc A. Does the Helping Babies Breathe Programme impact on neonatal resuscitation care practices? Results from systematic review and meta-analysis. *Acta Paediatr.* 2019;108(5):806-813.
33. *Tanzania Mainland poverty assessment.* 2015.

Table 1: Baseline characteristics of live births at Haydom 1.7.2013-30.6.2018 (n=18,997)

	**Period of birth**					
	**1st year** n=4,662	**2nd year** n=3,909	**3rd year** n=3,565	**4th year** n=3,664	**5th year** n=3,197	p-value*
**Maternal characteristics**						
**No antenatal care, n (%)**						0.16
	44 (0.9)	48 (1.2)	36 (1.0)	24 (0.7)	32 (1.0)	
**Antenatal problem, n (%)**						<0.001
	44 (0.9)	29 (0.7)	57 (1.6)	65 (1.8)	66 (2.1)	
**Preeclampsia/eclampsia, n (%)**						0.07
	21 (0.5)	17 (0.4)	25 (0.7)	26 (0.7)	28 (0.9)	
**Delivery**						
**Mode of delivery, n (%)**						<0.001
**Vaginal**	3,650 (78)	2,961 (76)	2,715 (76)	2,770 (76)	2,409 (75)	
**Caesarean**	924 (20)	943 (24)	824 (23)	858 (23)	749 (23)	
**Breech**	77 (2)	0 (0)	18 (0.5)	19 (0.5)	26 (0.8)	
**Vacuum/others**	11(0.2)	4 (0.1)	8 (0.2)	17 (0.5)	11 (0.3)	
**Fetal heart rate at admission, n (%)**						<0.001
**Normal**	4,122 (88)	3,510 (90)	3,274 (92)	3,444 (94)	3,006 (94)	
**Abnormal**	42 (1)	33 (1)	31 (1)	38 (1)	43 (1)	
**Not detectable**	2 (0.04)	0 (0)	2 (0.1)	5 (0.1)	0 (0)	
**Not measured**	496 (11)	365 (9)	257 (7)	177 (5)	145 (5)	
**Fetal heart rate during labour, n (%)**						<0.001
**Normal**	3,917 (84)	3,249 (83)	2,973 (83)	3,157 (86)	2,751 (86)	
**Abnormal**	143 (3)	168 (4)	168 (5)	230 (6)	298 (9)	
**Not detectable**	0 (0)	0 (0)	7 (0.2)	4 (0.1)	1 (0.03)	
**Not measured**	602 (13)	494 (13)	416 (12)	272 (7)	145 (5)	
**Last fetal heart rate before delivery (bpm), mean (SD)**						0.14
	134 (10)	134 (10)	134 (12)	134 (14)	133 (14)	
**Child characteristics**						
**Birth weight, Mean (SD)**	3215 (528)	3253 (520)	3280 (520)	3377 (539)	3342 (546)	<0.001
**<1500, n (%)**	24 (0.5)	24 (0.5)	15 (0.4)	23 (0.6)	28 (0.9)	
**1500-2500, n (%)**	325 (7)	218 (6)	199 (6)	158 (4)	136 (4)	
**2500-3499, n (%)**	2,895 (62)	2,397 (61)	2,071 (58)	1,821 (50)	1,690 (53)	
**3500-4499, n (%)**	1,370 (29)	1,228 (31)	1,242 (35)	1,594 (44)	1,284 (40)	
**≥4500, n (%)**	48 (1)	42 (1)	38 (1)	68 (2)	57 (2)	
**Gestational age < 37 weeks, n (%)**						0.042
	117 (2.5)	103 (2.6)	96 (2.7)	121 (3.3)	111 (3.5)	
**Female sex, n (%)**						0.049
	2,141 (46)	1,882 (48)	1,672 (47)	1,692 (46)	1,546 (48)	

bpm: beats per minute

* p-values calculated with chi-square for categorical and binary regression for continuous variables

1st year: 1.7.2013-30.6.2014
2nd year: 1.7.2014-30.6.2015
3rd year: 1.7.2015-30.6.2016
4th year: 1.7.2016-30.6.2017
5th year: 1.7.2017-30.6.2018

Table 2: Postnatal events and heart rate recordings of live births at Haydom 1.7.2013-30.6.2018 (n=18,997)

	Period of birth					
	**1st year**	**2nd year**	**3rd year**	**4th year**	**5th year**	p-value*
	n=4,662	n=3,909	n=3,565	n=3,664	n=3,197	
Apgar <5, n (%)						
1 min	37 (0.8)	50 (1.3)	42 (1.2)	45 (1.2)	66 (2.1)	<0.001
5 min	5 (0.1)	7 (0.2)	11 (0.3)	20 (0.6)	23 (0.7)	<0.001
Stimulation/suction*, n (%)						<0.001
No stim./suction	3,950 (85)	3,234 (83)	2,412 (68)	2,684 (73)	2,180 (68)	<0.001
Stimulation only	15 (0.3)	36 (0.9)	100 (2.8)	159 (4.3)	186 (6)	
Stim. and suction	697 (15)	641 (16)	1053 (30)	821 (22)	843 (26)	
Bag/mask ventilation	361 (7.7)	240 (6.1)	242 (6.8)	272 (7.4)	264 (8.8)	<0.001
Heart rate	n=389	n=298	n=274	n=262	n=232	
Initial heart rate beats/min median (IQR)	145 (97-179)	142 (94-171)	132 (84-174)	118 (70-162)	102 (67-148)	<0.001
Initial heart rate in categories, n (%)						
<60	32 (8)	25 (8)	21 (8)	36 (14)	50 (22)	<0.001
60-100	69 (18)	58 (19)	75 (27)	74 (28)	63 (27)	
100-160	139 (36)	98 (33)	85 (31)	80 (31)	75 (32)	
>160	149 (38)	117 (39)	93 (34)	72 (27)	44 (19)	
Mean heart rate during 0-60 seconds after initial recording, median (IQR)	143 (104-165)	139 (101-165)	139 (105-165)	130 (91-157)	129 (102-152)	<0.001

IQR: Inter-quartile range
* Chi2-test used for categorical analyses, linear regression for continuous analyses. P-values for trend.

Table 3: Relative risk for early neonatal death and perinatal death at Haydom 1.7.2013-30.6.2018

	Period of birth					Overall linear	p-value†
**Early neonatal deaths, n/total live births**	**1st year** 30/4,662	**2nd year** 41/3,909	**3rd year** 23/3,565	**4th year** 56/3,720	**5th year** 37/3,197		
**Unadjusted**							
	1 (ref.)	1.63 (1.02-2.61)	1.00 (0.58-1.72)	1.53 (0.94-2.47)	1.63 (1.11-2.90)	1.11 (1.00-1.24)	0.04
**Adjusted for abnormal fetal heart rate**							
	1 (ref.)	1.55 (0.97-2.48)	0.93 (0.54-1.59)	1.34 (0.83-2.18)	1.44 (0.88-2.33)	1.06 (0.95-1.18)	0.31
**Adjusted for birth weight and prematurity**							
	1 (ref.)	1.58 (1.00-2.48)	1.00 (0.59-1.70)	1.53 (0.95-2.44)	1.72 (1.08-2.72)	1.11 (1.00-1.23)	0.05
**Adjusted for initial heart rate**							
	1 (ref.)	1.72 (1.09-2.70)	0.96 (0.56-1.63)	1.34 (0.84-2.14)	1.41 (0.88-2.26)	1.04 (0.94-1.16)	0.41
**Adjusted for need for BMV**						1.09	
	1 (ref.)	1.88 (1.19-2.98)	1.10 (0.65-1.88)	1.57 (0.98-2.52)	1.72 (1.07-2.74)	1.09 (0.99-1.21)	0.39
**Final adjusted model***							
	1 (ref.)	1.88 (1.11-3.19)	0.92 (0.50-1.69)	1.25 (0.72-2.18)	0.93 (0.53-1.64)	0.95 (0.84-1.07)	0.39
**Sensitivity analysis excluding those never resuscitated (n=55), adjusted**							
	1 (ref.)	1.95 (0.99-3.81)	1.35 (0.66-2.73)	1.39 (0.71-2.72)	0.97 (0.49-1.91)	0.96 (0.83-1.11)	0.60
**Perinatal deaths n/total births**	106/4,738	99/3,967	62/3,604	92/3,720	77/3,235		
**Unadjusted**							
	1 (ref.)	1.12 (0.85-1.46)	0.77 (0.56-1.05)	1.11 (0.84-1.46)	1.06 (0.80-1.42)	1.01 (0.95-1.08)	0.77
**Final adjusted model***							
	1 (ref.)	1.58 (1.01-2.47)	0.88 (0.54-1.43)	0.87 (0.55-1.39)	0.53 (0.32-0.86)	0.84 (0.76-0.94)	0.002

* adjusted for antenatal problem, abnormal fetal heart rate during labour, birth weight, prematurity, initial heart rate and need for BMV after birth.

† p-value for trend

Excluded from adjusted analyses due to missing covariates: n=15

Table 4: Predictors (relative risk with 95% confidence intervals) for perinatal death at Haydom Lutheran Hospital during five years (n=19,264).

	**Unadjusted**	**Adjusted***
**Period, per year increase 2013-18**		
	1.01 (0.95-1.08)	0.84 (0.76-0.94)
**Fetal heart rate:**		
**normal**	1 (ref.)	1 (ref.)
**abnormal**	5.7 (4.4-7.3)	3.0 (2.0-4.5)
**not detected**	74.0 (64.5-84.9)	62.9 (20.5-193)
**Birth weight**		
**<1.5 kg**	28.7 (21.0-39.2)	23.6 (10.5-53.2)
**1.5-2.5 kg**	7.7 (5.8-10.1)	2.3 (1.3-4.1)
**2.5-3.5 kg**	1.4 (1.1-1.8)	1.0 (0.7-1.6)
**3.5-4.5 kg**	1 (ref.)	1 (ref.)
**>4.5 kg**	3.1 (1.6-5.9)	1.5 (0.5-5.1)
**Prematurity**		
	12.9 (10.7-15.6)	5.7 (3.3-9.9)
**Low initial heart rate after birth**		
**<60 bpm**	165 (131-208)	19.6 (11.1-34.9)
**60-100 bpm**	33.0 (23.3-46.6)	1.8 (1.0-3.2)
**100-160 bpm**	6.3 (3.5-11.3)	0.3 (0.2-0.8)
**>160 bpm**	2.4 (0.99-6.0)	0.2 (0.06-0.48)
**No need for recording**	1 (ref.)	1 (ref.)
**Need for BMV after birth**		
	74.0 (56.1-97.8)	28.7 (16.0-51.2)

*adjustments for fetal heart rate, birth weight category, prematurity, low initial heart rate and need for BMV after birth. In perinatal mortality results the initial heart rate and presumed need for BMV were set to the lowest category.

Table 5: Bag-mask ventilation characteristics of live births at Haydom 1.7.2013-30.6.2018 (n=18,997)

	**Period of birth**					
	**1st year**	**2nd year**	**3rd year**	**4th year**	**5th year**	**p-value***
	n=4,662	n=3,909	n=3,565	n=3,664	n=3,197	
Bag/mask-ventilation, time events	n=358	n=234	n=240	n=269	n=262	
Time from birth to BMV, seconds median,(IQR)	105 (74-149)	116 (81-164)	119 (88-158)	109 (74-162)	99 (74-153)	0.76
Duration of first BMV sequence in seconds, median (IQR)	11 (4-20)	11 (5-22)	16 (7-34)	23 (9-52)	32 (13-71)	<0.001
Duration of BMV first 60 seconds in seconds, median (IQR)	24 (15-36)	28 (16-41)	38 (22-51)	46 (33-54)	48 (35-59)	<0.001
Time from the first to the last inflation in seconds, median (IQR)	132 (56-248)	127 (59-340)	127 (66-298)	142 (64-282)	130 (73-301)	0.94
Bag/mask ventilation characteristics†	n=293	n=247	n=238	n=173	n=114	
Frequency of ventilations (inflations per minute)	66 (50-84)	52 (39-70)	44 (35-53)	48 (41-54)	47 (41-53)	<0.001
Tidal volume in ml/kg, median (IQR)	5.3 (3.3-8.4)	6.8 (4.3-10.0)	7.5(5.0-11.5)	6.6 (4.5-10.7)	6.2 (3.2-9.8)	<0.001
Mask leak in %, median (IQR)	45 (33-62)	46 (33-61)	42 (30-57)	41 (29-56)	44 (30-57)	0.001
Mean airway pressure mbar, median (IQR)	14 (12-17)	13 (11-16)	15 (12-18)	17 (14-21)	18 (16-21)	<0.001
Maximum airway pressure mbar, median (IQR)	34 (29-38)	33 (28-36)	36 (32-39)	38 (34-43)	40(37-45)	<0.001

IQR: Inter-quartile range.
BMV: Bag-mask ventilation.
* Chi2-test used for categorical analyses, linear regression for continuous analyses. P-values for trend.
† excluding participants in the PEEP-study, n=80 and n=123 in 4th and 5th year, respectively.
Two-step analysis: Mean of ventilation parameters for each newborn during first 5 minutes of active ventilation calculated, and in the second step this mean was used to calculate median/IQR.

Figure text:

Fig 1: Neonatal resuscitation monitor and its components used for data collection at Haydom Hospital during 1.7.13 to 30.6.18.

Fig 2: Flow chart of participants in a population-based study of births in Haydom hospital during 1.7.2013 to 30.6.2018.

Fig 3: Early neonatal mortality and stillbirths at Haydom Hospital by year during 1.7.13 to 30.6.18.

Figure Click here to access/download;Figure;Lysbilde1.TIF

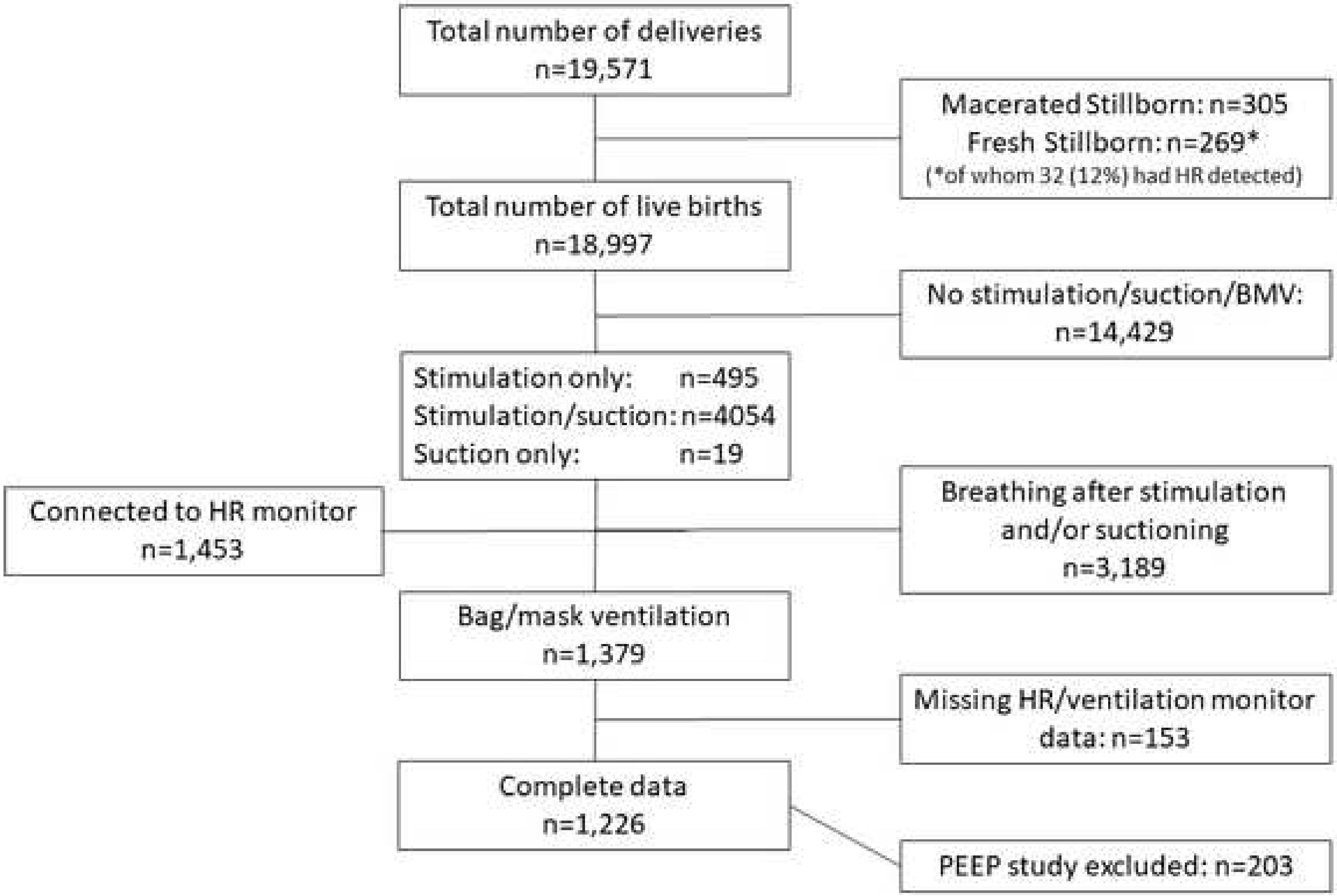

Figure 1

Click here to access/download;Figure;Fig 1.tif

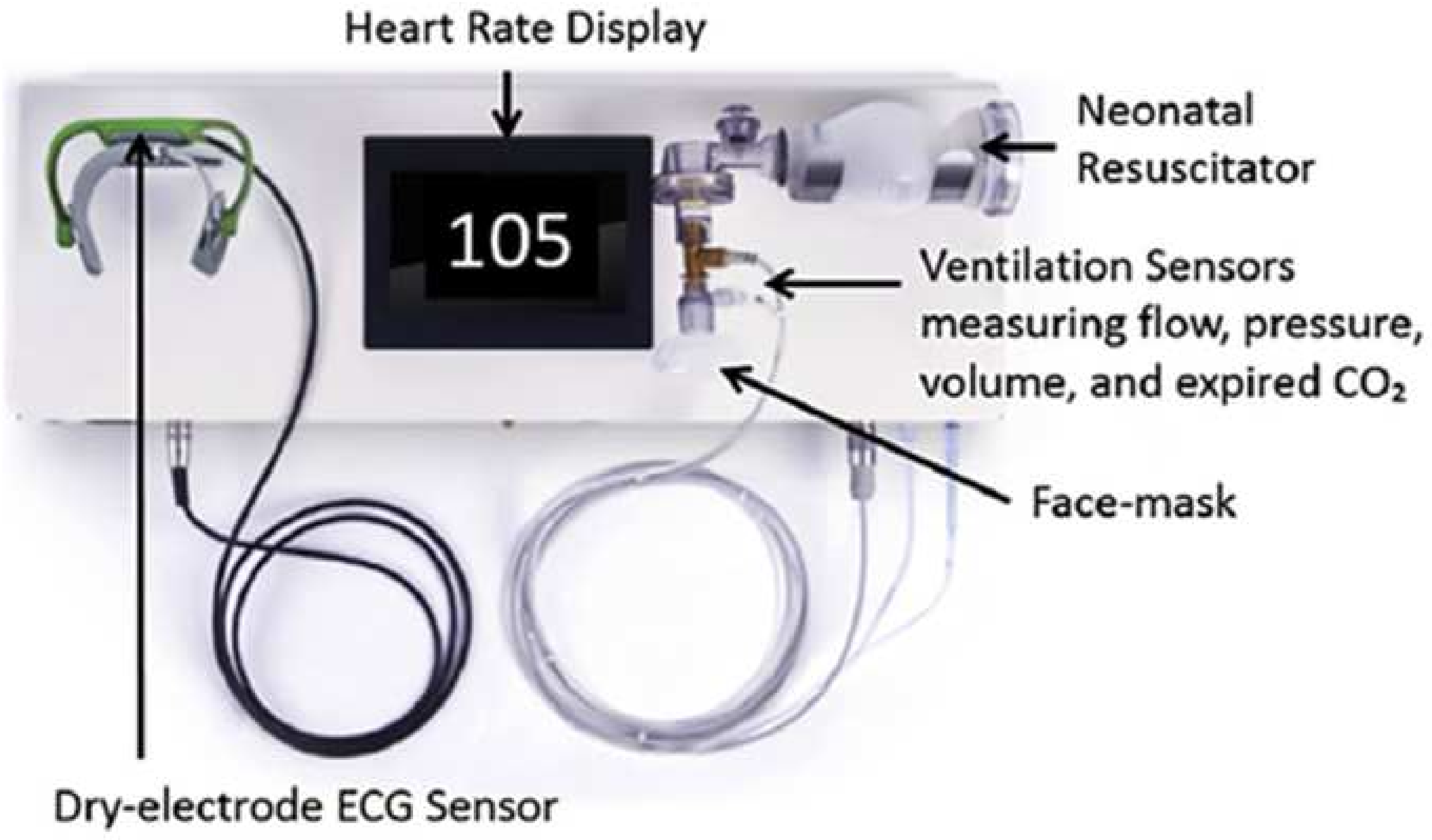

Figure 3

Click here to access/download;Figure;Fig 3.tif

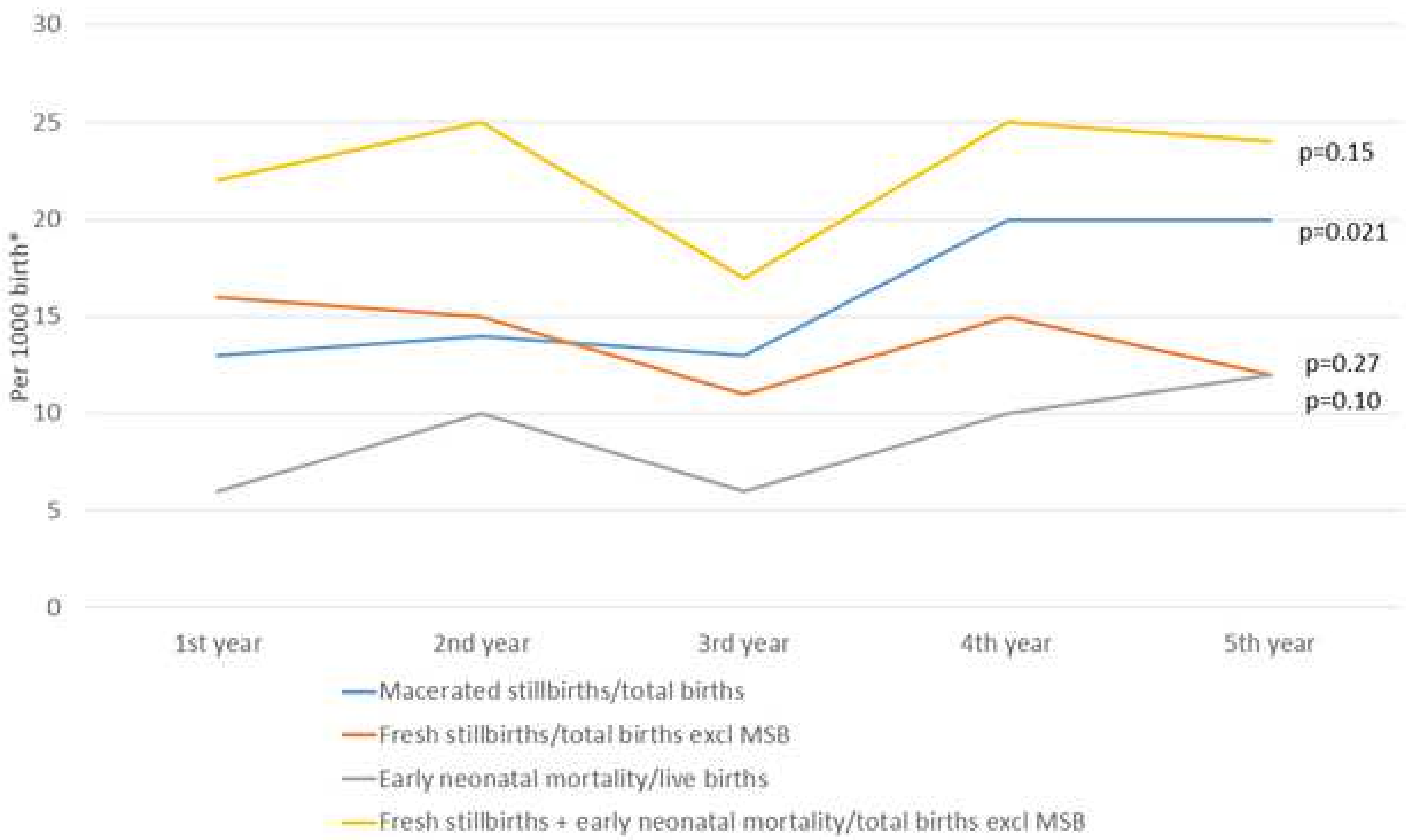

Permission to publish NIMR

Click here to access/download
Supporting Information
Permission to Publish - Hussein Kidanto 29.10.19.pdf

Click here to access/download
Supporting Information
NIMR_Safer Births Approval Certificate_2012.pdf

Increased perinatal survival and improved ventilation skills over a five-year period: An observational study

Ketil Størdal[1,2], Joar Eilevstjønn[3], Estomih Mduma[4], Kari Holte[1], Monica Thallinger[5], Jørgen Linde[6], Paschal Mdoe[4], Hussein Kidanto[7], Hege Ersdal[6,8].

[1] Ostfold Hospital Trust, Grålum, Norway.

[2] Norwegian Institute of Public Health, Oslo, Norway.

[3] Laerdal Medical AS, Stavanger, Norway.

[4] Haydom Lutheran Hospital, Manyara, Tanzania.

[5] Vestre Viken HF, Gjettum, Norway.

[6] Helse Stavanger HF, Stavanger, Norway.

[7] Muhimbili National Hospital, Dar Es Salam, Tanzania.

[8] SAFER, Stavanger, Norway.

Contact author: Ketil Størdal, Division for Mental and Physical Health, Norwegian Institute of Public Health, PO Box 222 Skøyen, 0213 Oslo, Norway. Phone 0047 48272337, ketil.stordal@fhi.no

Formatted: Superscript
Formatted: Norwegian (Bokmål)
Formatted: Superscript
Formatted: Norwegian (Bokmål)
Formatted: Superscript
Formatted: Norwegian (Bokmål)
Formatted: Superscript
Formatted: English (United States)
Formatted: English (United States), Not Superscript/ Subscript
Formatted: Not Superscript/ Subscript
Formatted: English (United States)
Formatted: Space After: 0 pt, Line spacing: Double
Formatted: Not Superscript/ Subscript
Formatted: Not Superscript/ Subscript
Formatted: English (United Kingdom), Not Superscript/ Subscript
Formatted: English (United States)
Formatted: English (United States), Not Superscript/ Subscript
Formatted: Not Superscript/ Subscript
Formatted: Not Superscript/ Subscript
Formatted: Norwegian (Bokmål), Superscript
Formatted: Norwegian (Bokmål)
Formatted: Superscript
Formatted: English (United States), Superscript
Formatted: English (United States)
Formatted: Superscript
Formatted: English (United States)
Formatted: English (United States)

Abstract

Background and aim:

The Helping Babies Breathe program gave major reductions in perinatal mortality in Tanzania from 2009 to 2012. We aimed to study whether this effect was sustained, and whether resuscitation skills changed with continued frequent training.

Methods:

We analysed prospective data covering all births (n=19,571) at Haydom Lutheran Hospital in Tanzania from July 2013 – June 2018. Resuscitation training was continued during this period. All deliveries were monitored by an observer recording the timing of events and resuscitation interventions. Heart rate was recorded by dry-electrode ECG and bag-mask-ventilation by sensors attached to the resuscitator device. We analy~~s~~zed changes over time in outcomes, use of resuscitation interventions and performance of resuscitation using binary regression models with the log-link function to obtain adjusted relative risks.

Results:

With introduction of user fees for deliveries since 2014, the number of deliveries decreased by 30% from start to the end of the five-year period. An increase in ~~complicated pregnancies, preterm deliveries, foetal heart rate abnormalities,~~ low heart rate at birth and need for bag-mask-ventilation indicate a gradual selection of more vulnerable newborns delivered in the hospital over time.
Despite this selection, newborn deaths <24 hours did not change significantly and was maintained at an average of 8.8/1000 live births. The annual reductions in relative risk for perinatal death adjusted for vulnerability factors was 0.84 (95%CI 0.76-0.94).
~~Ventilation monitoring showed longer ventilation sequences without interruption and increasing duration of ventilation the first minute by time.~~ During the five-year period, longer duration of bag-mask ventilation sequences without interruption was observed. Delivered tidal volumes were increased and mask leak was decreased during ventilation. ~~We also found significantly higher tidal volumes and lower mask leak.~~ The time to initiation or total duration of ventilation did not change significantly.

Conclusion:

The reduction in 24-hour newborn mortality after introduction of Helping Babies Breathe was maintained, and~~with~~ a further decrease over the five-year period was evident when analyses were ~~after~~ adjusted~~ment~~ for vulnerability of the newborns. Perinatal survival and performance of ventilation were significantly improved. ~~Lower rates of births in a safe hospital environment observed during this period is of concern.~~

Introduction

The reduction of neonatal deaths over the last decade has been modest, despite declining mortality of infants between 1 month and five years of age

~~In an era with declining mortality from 1 month to five years, the reduction in neonatal deaths has been slower.~~[1] The estimated global neonatal mortality (≤ 28 days) ~~has fallen~~fell from 31/1000 in 2000 to 18/1000 in 2017.[2] The annual rate reduction in neonatal mortality was 3.1% compared to 4.7% ~~Due to the rapid decline in mortality~~ after the neonatal period. Consequently, the relative contribution of neonatal deaths in the global under-5-mortality has increased from 39% to 45%.[1] The majority of these neonatal deaths are preventable. Safer births ~~can be obtained~~are achievable by improvements in a continuum from~~starting with~~ pregnancy ~~care~~, obstetric ~~care~~ to~~and~~ neonatal care, each accounting for 1/3 of preventable deaths.[3]

Intrapartum-related events, formerly referred to as birth asphyxia and defined as inadequate onset of spontaneous breathing after birth, are~~is~~ one of the three most prevalent causes of under-5-mortality.[1] The Helping Babies Breathe (HBB) program ~~was developed~~aimed to reduce death from intrapartum events in low- and middle-income settings, through training of health care workers in early identification and treatment.[4]

In Tanzania, the roll-out of HBB ~~was rolled out~~ from 2010 was associated~~,~~ with a 47% reduction in fresh stillbirths and early neonatal mortality over~~seen after~~ 24 months in eight large birth facilities.[5] Haydom Lutheran Hospital was one of the eight sites participating in the early roll-out of HBB. ~~Since the launch of HBB at Haydom in 2010,~~ HBB training and research related to safer deliveries and newborn resuscitations ~~have~~ continued in the Safer Births program.[6] Frequent low-dose training is one of the key elements to improve resuscitation skills.[6,7]

In Tanzania ~~has seen a reduction in~~ neonatal mortality decreased from 33/1000 in 2000 to 21/1000 in 2017.[2] However, ~~V~~vital birth statistics are~~, however, in general~~ not available,[8,9] and estimates ~~continue to be~~are inaccurate as almost half of the deliveries in Tanzania take

Formatted: Font: (Default) +Body (Arial), 11 pt, English (United States)

Formatted: Font: (Default) +Body (Arial), 11 pt, English (United States)

Formatted: Line spacing: Double

Formatted: Font: (Default) +Body (Arial), 11 pt

Formatted: English (United States)

Formatted: Space After: 0 pt, Don't adjust space between Latin and Asian text, Don't adjust space between Asian text and numbers

Formatted: English (United States)

Formatted: English (United States)

Formatted: English (United States)

Formatted: English (United States)

Formatted: English (United States)

Formatted: English (United States)

Formatted: English (United States)

Formatted: English (United States)

Formatted: English (United States)

Formatted: English (United States)

Formatted: English (United States)

Formatted: English (United States)

Formatted: English (United States)

Formatted: English (United States)

Formatted: English (United States)

Formatted: English (United States)

Formatted: English (United States)

Formatted: English (United States)

Formatted: English (United States)

Formatted: English (United States)

Formatted: English (United States)

Formatted: English (United States)

place outside birth facilities. In a rural area with a low proportion of deliveries in hospital, this selection may preclude interpretation of temporal trends in mortality rates. It is therefore pertinent to adjust for vulnerability background factors in hospital deliveries.

~~In the present study, we aimed to document changes and predictors of 24-hour neonatal mortality and fresh stillbirths during a five-year period, after~~ After introduction of ~~frequent~~ HBB ~~trainings in 2011~~ in 2009-2012~~.~~, efforts to sustain the initial improvements in perinatal mortality ~~We further studied changes in resuscitation performance in this facility with an ongoing~~ continued with onsite low-dose high-frequency HBB training ~~focus~~. ~~-~~In the present study, we aimed to document changes in 24-hour neonatal mortality, fresh stillbirths and resuscitation performance during a five-year period from 2013 to 2018.

Methods

Haydom Lutheran Hospital is situated in the rural area of the Manyara region of Northern Tanzania. The hospital has a catchment area of over 2 million inhabitants, and is the only hospital in the region providing comprehensive obstetric care with 250-400 deliveries per month. Haydom is a non-governmental hospital managed by the Lutheran church of Tanzania. ~~Due to financial constraints, delivering mothers have since the beginning of 2014 been charged with a fee of 12 USD for vaginal deliveries and 30 USD for caesarean section in addition to fees for ambulance transport. The number of births in the hospital has thereafter fallen substantially, likely due to the costs of delivery care.~~[6] All births in the hospital from 1st of July 2013 until 30th of June 2018 were recorded in the study. ~~*Ethical consideration*~~ This study was approved by the National Institute for Medical Research, Tanzania (NIMR/HQ/R8a/Vol. IX/1434) and the Regional Committee for Medical and Health Research Ethics, Western Norway (2013/110/REK). All health care providers gave informed oral consent. The mothers were informed but formal consent was not obtained for this observational study due to the urgency of mothers arriving in late stages of labor. This consent procedure was approved by the ethical committees.

~~Patients were informed, but consent was not obtained for this descriptive study as approved by the ethical committees.~~

The initial HBB implementation

The local procedure for newborn resuscitation followed the HBB guidelines as implemented locally from 2009-2012[5], emphasizing stimulation and early initiation of bag-mask ventilation (BMV).[4] Newborn resuscitation was mainly the responsibility of midwives. ~~There were s~~Six labour rooms were~~,~~ each equipped with a resuscitation table. After caesarean section,

Formatted: English (United States)

Formatted: English (United States)

Formatted: English (United States), Superscript

Formatted: English (United States)

Formatted: English (United States), Superscript

Formatted: English (United States)

Formatted: Line spacing: Double

Formatted: English (United States)

Formatted: Font: Italic, English (United States)

Formatted: English (United States)

Formatted: Line spacing: Double

Formatted: English (United States)

Formatted: English (United States)

Formatted: English (United States)

Formatted: English (United States)

newborn resuscitation was performed in a room adjacent to the operating theatre. After resuscitation, the midwives decided based on the clinical condition whether to keep the newborn with the mother or transfer to a neonatal ward providing basic care without ventilation support equipment except supplemental oxygen on nasal prongs. Midwives determined if the newborn remained with the mother or was transferred to a neonatal ward after resuscitation. The neonatal ward provided basic care: respiratory support was limited to provision of supplemental oxygen with nasal prongs.

The Safer Births program

The Safer Births program, starting in 2013, aimed to further facilitate HBB training and improve obstetric and newborn care by low-cost technology.[6,10] Prior to and dDuring the study all midwives were encouraged to low-dose, high-frequency skills training as described by Mduma et al.[11] Briefly, simulation training was performed on manikins, assisted by local-trainers who had undergone facilitator training for the HBB program. Visiting instructors from other places in Tanzania and Norway also participated regularly. The trainings were intended to take place weekly, but with some variations over time. There were six labour rooms, each equipped with a resuscitation table. After caesarean section, newborn resuscitation was performed in a room adjacent to the operating theatre. After resuscitation, the midwives decided based on the clinical condition whether to keep the newborn with the mother or transfer to a neonatal ward providing basic care without ventilation support equipment except supplemental oxygen on nasal prongs.

The Safer Births program, starting in 2013, aimed to further facilitate HBB training and improve obstetric and newborn care by low-cost technology.[6,10]

Trained data collectors observed and recorded the timing of events immediately after birth, and vital data and outcomes from all deliveries were recorded in a database.

Formatted: Font: (Default) +Body (Arial), 11 pt, English (United States)

Formatted: English (United States)

Formatted: Font: Italic, English (United States)

Formatted: English (United States)

Formatted: English (United States)

Formatted: English (United States)

Formatted: English (United States)

Formatted: English (United States)

Formatted: English (United States)

Formatted: English (United States)

Formatted: English (United States)

Physiological data collected from 1st July 2013 until 30 Jun, 2018 included heart rate data recorded from ECG, and ventilation parameters obtained from sensors connected to the ventilation bag using a Newborn Resuscitation Monitor (Laerdal Global Health, Stavanger, Norway) (Fig 1). ~~Since 1st of July 2013 recordings of heart rate data by ECG dry electrodes and ventilation parameters by a flow sensor connected to the ventilation bag have been stored for research purposes.~~ The arch-shaped ECG sensor was placed over the thorax or abdomen of the newborn. Two stainless-steel discs arranged on each side of the flexible arch acted as dry electrodes. The monitor used a hot-wire flow sensor connected to the ventilation bag similar to that in the Florian Respiratory Function Monitor (Acutronic Medical Systems, Hirzel, Switzerland). Volume was calculated by flow signal integration. Pressure was measured using an MPX5010 sensor (Freescale Semiconductor, Austin, TX, USA). The HR was displayed on the monitor installed in front of the resuscitation table visible to the provider, ventilation parameters were not displayed. ~~Details about the monitors and the frequent HBB training has been published elsewhere.~~[12] We analysed data for a five years period with collection of physiological data until 30th of June 2018 (Fig ~~1~~2, flow chart).

~~For the analysis of ventilation parameters, we excluded those ventilated with a valve providing PEEP (Positive End Expiratory Pressure) in a trial enrolling participants from September 2016 to June 2018, because this intervention is likely to change mean airway pressures and tidal volumes.~~

Main ~~outcome~~ and ~~exposure~~secondary outcomes

The main outcome was early neonatal mortality (ENM) defined as death before 24 hours of life among newborns delivered alive at Haydom Lutheran Hospital. As shown previously, live born babies with severe asphyxia may be misclassified as fresh stillbirths ~~(FSB).~~[13,14] Therefore, we also used the combined outcome of ENM and fresh stillbirths~~FSB~~ to analy~~s~~ze perinatal ~~deaths~~mortality.

Formatted: Font: (Default) +Body (Arial), 11 pt, English (United States)

Formatted: Don't adjust space between Latin and Asian text, Don't adjust space between Asian text and numbers

Formatted: Font: (Default) +Body (Arial), 11 pt, English (United States)

Formatted: Font: (Default) +Body (Arial), 11 pt, English (United States)

Formatted: Font: (Default) +Body (Arial), 11 pt, English (United States)

Formatted: English (United States)

Formatted: English (United States)

Formatted: English (United States)

Formatted: English (United States)

Formatted: English (United States)

Formatted: English (United States)

Formatted: English (United States)

Formatted: English (United States)

Formatted: English (United States)

Formatted: Line spacing: 1.5 lines

Formatted: English (United States)

Formatted: English (United States)

Formatted: English (United States)

The secondary outcomes were clinical performance defined by timing of events during resuscitation: Time from birth to start of BMV, the continuation of BMV by measuring duration of the first ventilation sequence before pause (>5 seconds interruption) and the fraction of time used for BMV once it had been initiated. To assess actual bag-mask ventilation performance, we studied ventilation frequency, tidal volumes and mask leak the first five minutes of bag-mask ventilation.

Main exposure and covariates

We analyszed by year (1st of July – 30th of June, starting 1st of July 2013) for the five-year period to assess effects of ongoing training in newborn resuscitation over time.

To assess for potential confounding, we obtained information from the prospective database of pregnancy complications, birth weight, prematurity, fetal heart rate abnormalities, the last recorded fetal heart rate and the need for BMV at birth and type of resuscitator used.

Vulnerability factors

The reduction in deliveries at the hospital during the period, was likely to introduce a selection bias. More complicated deliveries were likely conducted or transferred to the hospital with impact on mortality rates of those delivering in the hospital. To account for potential vulnerability factors, we adjusted mortality rates for pregnancy complications, birth weight, prematurity, foetal heart rate abnormalities, last recorded foetal heart rate and the need for BMV at birth.[13,15] For the analysis of ventilation parameters, we excluded those ventilated with a valve providing PEEP (Positive End Expiratory Pressure) in a trial enrolling participants from September 2016 to June 2018, because this intervention is likely to change mean airway pressures and tidal volumes.

Formatted: Font: Italic, English (United States)
Formatted: English (United States)
Formatted: Font: Italic, English (United States)
Formatted: English (United States)
Formatted: English (United States)
Formatted: English (United States)
Formatted: English (United States)

~~*Neonatal resuscitation performance*~~

~~We aimed to study whether frequent training on neonatal resuscitation through the HBB program affected clinical performance. We analysed timing of events by studying time to start of BMV, duration of the first ventilation sequence before pause (>5 seconds interruption) and fraction of time used for BMV once initiated. To assess ventilation performance, we studied ventilation frequency, tidal volumes and mask leak.~~

Statistical methods

Descriptive analyses were performed by calculation of medians with interquartile ranges (IQR) or means with standard deviations according to their distribution. Categorical variables were analyzed using percentages and Chi-square test.

We used binary ~~linear~~ regression with log-link function to estimate relative risks for ENM by time.[13,15] ~~Vulnerability f~~Risk factors which ~~showed~~ changesd over time and were associated with the outcome (p<0.10) were included in a multivariate regression model if they, changed the relative risk estimates by >10%. In the final model we therefore adjusted for birth weight, prematurity, fetal heart rate abnormalities, last recorded fetal heart rate and the need for BMV at birth. Cases with missing variables for any of the included adjustment variables (n=15) were excluded from the adjusted model.

We also performed sensitivity analyses excluding newborns who died without a history of resuscitation at birth because conditions other ~~conditions~~ than intra-partum hypoxia may cause ENM.

Data were extracted using Matlab 2019a (MathWorks, Natick, MA) and analyzed using Stata SE 15 (StataCorp. 2017. Stata Statistical Software: Release 15. College Station, TX: StataCorp LP.)

~~*Ethical consideration*~~

~~This study was approved by the National Institute for Medical Research, Tanzania~~

Formatted: English (United States)

Formatted: English (United States)

Formatted: Don't keep with next

Formatted: English (United States)

Formatted: English (United States)

Formatted: English (United States)

Formatted: English (United States)

Formatted: English (United States)

Formatted: English (United States)

(NIMR/HQ/R8a/Vol. IX/1434) and the Regional Committee for Medical and Health Research Ethics, Western Norway (2013/110/REK). All health care providers gave informed oral consent. Patients were informed, but consent was not obtained for this descriptive study as approved by the ethical committees.

Results

During the five-year period, 18,997 infants were born alive, with a reduction over time from 4,662 to 3,197 annual births (Table 1). ENM fluctuated around 8.8/1000 live births, and the combined outcome of perinatal mortality (fresh stillbirths and ENM) ~~FSB and ENM~~ around 22.6/1000 with no significant differences over time (~~Table 2~~Fig 3).

Vulnerability factors over time

During the observation period, the~~Concomitant with a 31% reduction in deliveries in the hospital during the observation period, a higher~~ proportion of deliveries ~~was~~ characterized by risk factors for adverse outcome increased. The proportion of pregnancies with antenatal problems including preeclampsia increased significantly from 0.9~~<~~1% to ~~>~~2.1% (p<0.001). The mean birth weight increased slightly, but an increasing proportion were <1500 g and preterm births increased from 2.5% to 3.5% (Table 1).

Abnormal f~~o~~etal heart rate during delivery increased gradually from 3% to 9%. The first detected heart rate among resuscitated babies fell from a median of 142 to 108 beats per minute (bpm), and the proportion with heart rate <100 bpm at birth increased from 8% to 22% (p<0.001, Table ~~3~~2).

Mortality adjusted for vulnerability

In unadjusted analyses, the ENM increased slightly during the five-year period (relative risk per year 1.11, 95%CI 1.00-1.24, Table ~~4~~3). Vulnerability factors all increased during this period, as shown in Table 1 and ~~3~~2. After adjustment for abnormal f~~o~~etal heart rate during delivery, birth weight, ~~/gestational age~~prematurity, initial heart rate and need for BMV at birth, there was ~~an annual non-significant 5%~~no significant reduction in relative risk for ENM (relative risk per year 0.95, 95%CI 0.84-1.07, Table ~~4~~3). Death the first 24 hours could be due to other reasons than intrapartum events, especially prematurity. Of those who died, 55/169 (33%) had not been ventilated, and thus would likely not be diagnosed as

Formatted: English (United States), Not Highlight

Formatted: English (United States)

asphyxiated. We performed a sensitivity ~~repeated the~~ analys~~e~~is without these 55: The unadjusted and adjusted analyses were essentially unchanged ~~(data not shown)~~(Table 3).

The ~~combined outcome of~~ perinatal mortality ~~(FSB and ENM)~~ showed no appreciable change during the five-year period in unadjusted analyses. However, after adjustment for vulnerability factors we found a significant~~n~~ annual decrease of 16% in perinatal mortality (relative risk per year 0.84, 95%CI 0.76-0.94, Table ~~4~~3).

In the fully adjusted model, year of birth, f~~o~~etal heart rate abnormalities, initial heart rate, birth weight, prematurity and need for BMV predicted perinatal death (Table ~~5~~4).

Changes in resuscitation performance

The proportion who received BMV varied from 6.1-7.7%, but increased to 8.8% the last year (Table 2~~3~~). Time from birth to start of BMV was lowest the last year (median 99 seconds) but did not change significantly over time (p=0.76, Table 5). The duration of the first BMV sequence increased over time from a median of 11 to 32 seconds (p<0.001, Table 5).~~, and active ventilation~~ Once initiated, BMV was continued for an increasing part of the first 60 seconds, from a median of 24 seconds the first compared to 48 seconds the last year (p<0.001, Table 5). The duration of ventilation from the first to the last inflation remained stable (Table 5~~3~~).

Median ventilation frequency decreased from >60 breaths/min the first year to 44-48 breaths/min the last three years of observation (p<0.001). The median weight-adjusted tidal volume, mean and peak airway pressures increased (p<0.001), whereas mask leak decreased slightly but significantly over time (p=0.001, Table ~~3~~5).

Formatted: English (United States)

Formatted: English (United States)

Formatted: English (United States)

Formatted: English (United States)

Discussion

During a five-year period following a successful implementation of frequent HBB training, we found no further significant decline in ENM. A worrying decrease in hospital deliveries was observed, likely due to introduction of user fees since 2014~~,~~ of 12 and 30 USD for vaginal and caesarean section deliveries, respectively. Consequently, hospitalized newborns included a higher proportion of infants with increased vulnerability for adverse outcomes~~, and newborns with a higher vulnerability for adverse outcome were born in the hospital~~. In analyses adjusted for vulnerability factors, perinatal deaths decreased significantly during the five years.

~~**Strengths and limitations**~~

Among several strengths in the study is the large size population-based study, performed in a rural setting of a low-income country with high burden of disease. The prospective design ensured high-quality data from the delivery and immediate post-natal period. Collection of physiological data during the first minutes of life provides information which is rarely accessible.

The selection of higher-risk deliveries during the period is evident, and accounted for as far as possible in the adjusted analysis. However, as in any observational study, residual confounding cannot be completely ruled out. A population-based birth registry with inclusion of all pregnancies including home deliveries would capture the complete picture and avoid selection bias. Specific causes of ENM were not possible to identify in our study, though in a sub-study from this cohort half of the deaths were related to intra-partum events.[16] We were, however, able to exclude deaths unlikely to be caused by perinatal hypoxia in a sensitivity analysis. An increased use of BMV ventilation over the 5-year period likely reflects the higher morbidity, and Apgar scores in this setting have previously been shown to have low

Formatted: Font: (Default) +Body (Arial), 11 pt, English (United States)

Formatted: Font: (Default) +Body (Arial), 11 pt, English (United States)

Formatted: English (United States)

Formatted: English (United States)

Formatted: English (United States)

Formatted: English (United States), Not Highlight

Formatted: English (United States)

prognostic value.[17] However, we cannot rule out that a sustained focus on HBB training may increase unnecessary resuscitation procedures.

Comparison with other studies

During the nation-wide HBB roll-out during 2010/2011 in Tanzania, ENM in eight large hospitals including Haydom decreased from 13.4 to 7.1/1000. Fresh stillbirthsFSB decreased from 19 to 14.4/1000, and a remarkable fall of the combined outcome of FSB and ENMperinatal mortality of 34% was reported.[5] ENM in Haydom before and after implementation of HBB in 2011 was 11.1 and 7.2/1000, respectively. Before and after initial implementation of the HBB program ENM at Haydom was 11.1/1000 and 7.2/1000 respectively. [11] The baseline perinatal mortality was 27/1000 and fell to 21.7/1000, and the decrease in ENM thus accounted for 74% of the reduced perinatal mortality.[18] In a follow-up study, this reduction was maintained until end of observation in January 2017.[6] In the present extended follow-up, our period of observation starts two years after regular HBB implementation and provides information on perinatal mortality, resuscitation performance and physiological data during a five-year period and extends further 1.5 years[6]. Our study shows that the initial reduction in perinatal mortality seen after HBB implementation has continued.

HBB has been introduced globally, and overall a reduction in perinatal mortality has been reported.[19] The variation between countries with highly successful implementation [5,20,21] to sites reporting no or modest effect on mortality [22,23] is of note. The relative contribution of intra-partum hypoxia to ENM across countries may explain some differences, because a lower effect would be expected when other causes of ENM dominate. Besides, implementation and uptake of HBB could influence the results, and repeated low-dose onsite training is likely to facilitate successful implementation.[11] Several studies have reported

Formatted: English (United States)
Formatted: English (United States)
Formatted: English (United States)
Formatted: English (United States)
Formatted: English (United States)
Formatted: English (United States)
Formatted: English (United States)
Formatted: English (United States)
Formatted: English (United States)
Formatted: English (United States)
Formatted: English (United States)
Formatted: English (United States)
Formatted: English (United States)
Formatted: English (United States)
Formatted: English (United States)
Formatted: English (United States)
Formatted: English (United States)
Formatted: English (United States)
Formatted: English (United States)
Formatted: English (United States)

improved resuscitation knowledge and practical skills after HBB training in a simulation setting.[4,24] Some loss of performance over time is observed,[25-27] but skills retention was improved by frequent re-training and use of self-evaluation checklists.[26] Importantly, changes in clinical management do not necessarily follow simulated performance.[28] The present study documents improved resuscitation skills that are sustained over time, and is likely to improve perinatal mortality. Heart rate feedback during resuscitation may also have improved skills over time, as suggested in a qualitative study from Haydom.[7]

In a study from Nepal, a reduction ~~of~~ in the percentage of deaths attributable ~~due~~ to intra-partum related complications from 51 to 33% was observed, but overall newborn mortality was unchanged.[29] This was also reported in the recent meta-analysis, pointing to the importance of a package of interventions needed to address the main causes of neonatal death.[19] In our study, one third of deaths occurred in newborns~~, mainly preterms,~~ that did not receive BMV after birth. Intra-partum events accounted for about half the deaths before 7 days age ~~is the leading cause of death~~ at Haydom during 2014-17, but preterm birth and sepsis were~~are~~ among other preventable causes of death.[16]

Our study is the first to report long-term changes in performance of resuscitation and BMV ~~sustained effect on mortality~~ after HBB implementation. Analyses of actual ventilation performance demonstrate that practical skills improved over time with significantly longer duration of the first ventilation sequence and reduction in ventilation frequency targeting the recommended range. The increase in more complex patients may also change the resuscitation practice over time. The reduction in mask leak over time was ~~also~~ desirable though admittedly small. Higher tidal volumes are associated with more rapid HR increase in observational studies from Haydom,[30,31] but should be further studied in controlled trials before we can conclude whether this is recommendable.

In the initial HBB roll-out at Haydom, the use of suction/stimulation increased whereas use of BMV decreased significantly.[11] We found a further increase in suction/stimulation but also of BMV, which could be expected with increased vulnerability over time. In a meta-analysis of the effect of HBB training on resuscitation practices from four studies, the overall use of suctioning, stimulation or BMV was unchanged. However, interventions during the first Golden minute increased 2.5-fold.[32] In our study ~~-~~the increase during the five years of time used to ventilate the first minute after BMV was started ~~the reduced time from birth to BMV initiation the last year of observation~~ is of note. The intensity and frequency of HBB training varied during the 5-year period, and due to shifts in the staff the quality of birth care and post-natal care is likely to be vulnerable. The substantial fluctuation over time in perinatal death rates, was however not reflected in the data on resuscitation performance.

Formatted: English (United States)

Formatted: English (United States)

Formatted: English (United States)

Formatted: English (United States)

Formatted: English (United States)

Formatted: English (United States)

Formatted: English (United States)

~~**Implications**~~

In the present study the quality of immediate newborn care at birth was improved over time, probably due to continuous focus on frequent HBB training. However, coverage and access are likely even more important for the total perinatal mortality than quality of care in the hospital. These factors are interrelated, as increased quality in a delivery unit is likely to encourage more pregnant women to seek care. However, financial constraints may counteract this effect. In Tanzania in 2011/12, 44% had less than 1.25 USD per day for their daily living, and poverty was even more prevalent in rural districts.[33] In this context, 12 to 30 USD in hospital fees for delivery is a considerable burden to the family economy. Free delivery care should be a high priority, as provided in governmental hospitals in Tanzania.

Formatted: English (United States)

Formatted: English (United States)

Formatted: English (United States)

In this study we have shown that neonatal resuscitation skills improved over time at a site with high focus on continued and frequent HBB training. The perinatal mortality adjusted for

vulnerability factors decreased, whereas ENM showed a non-significant decline. Further reduction in neonatal mortality in Tanzania will likely require a comprehensive approach to increase facility births and address the major causes of death.

1. Liu L, Oza S, Hogan D, et al. Global, regional, and national causes of under-5 mortality in 2000-15: an updated systematic analysis with implications for the Sustainable Development Goals. *Lancet.* 2016;388(10063):3027-3035.
2. Hug L, Alexander M, You D, Alkema L, Estimation UNI-aGfCM. National, regional, and global levels and trends in neonatal mortality between 1990 and 2017, with scenario-based projections to 2030: a systematic analysis. *Lancet Glob Health.* 2019;7(6):e710-e720.
3. Bhutta ZA, Das JK, Bahl R, et al. Can available interventions end preventable deaths in mothers, newborn babies, and stillbirths, and at what cost? *Lancet.* 2014;384(9940):347-370.
4. Singhal N, Lockyer J, Fidler H, et al. Helping Babies Breathe: global neonatal resuscitation program development and formative educational evaluation. *Resuscitation.* 2012;83(1):90-96.
5. Msemo G, Massawe A, Mmbando D, et al. Newborn mortality and fresh stillbirth rates in Tanzania after helping babies breathe training. *Pediatrics.* 2013;131(2):e353-360.
6. Mduma E, Kvaloy JT, Soreide E, et al. Frequent refresher training on newborn resuscitation and potential impact on perinatal outcome over time in a rural Tanzanian hospital: an observational study. *BMJ Open.* 2019;9(9):e030572.
7. Moshiro R, Ersdal HL, Mdoe P, Kidanto HL, Mbekenga C. Factors affecting effective ventilation during newborn resuscitation: a qualitative study among midwives in rural Tanzania. *Glob Health Action.* 2018;11(1):1423862.
8. Rebnord T, Ostbye T, Mmbaga BT, McHome B, Lie RT, Daltveit AK. Time trends in management of HIV-positive pregnant women in Northern Tanzania: A registry-based study. *PLoS One.* 2017;12(9):e0184362.
9. Bergsjo P, Mlay J, Lie RT, Lie-Nielsen E, Shao JF. A medical birth registry at Kilimanjaro Christian Medical Centre. *East Afr J Public Health.* 2007;4(1):1-4.
10. http://www.saferbirths.com/. Accessed 04.10.2019.
11. Mduma E, Ersdal H, Svensen E, Kidanto H, Auestad B, Perlman J. Frequent brief on-site simulation training and reduction in 24-h neonatal mortality--an educational intervention study. *Resuscitation.* 2015;93:1-7.
12. Linde JE, Schulz J, Perlman JM, et al. Normal Newborn Heart Rate in the First Five Minutes of Life Assessed by Dry-Electrode Electrocardiography. *Neonatology.* 2016;110(3):231-237.
13. Thallinger M, Ersdal HL, Francis F, et al. Born not breathing: A randomised trial comparing two self-inflating bag-masks during newborn resuscitation in Tanzania. *Resuscitation.* 2017;116:66-72.
14. Ersdal HL, Eilevstjonn J, Linde JE, et al. Fresh stillborn and severely asphyxiated neonates share a common hypoxic-ischemic pathway. *Int J Gynaecol Obstet.* 2017.
15. Thallinger M, Ersdal HL, Morley C, et al. Neonatal ventilation with a manikin model and two novel PEEP valves without an external gas source. *Arch Dis Child Fetal Neonatal Ed.* 2017;102(3):F208-F213.
16. Moshiro R, Perlman JM, Mdoe P, Kidanto H, Kvaloy JT, Ersdal HL. Potential causes of early death among admitted newborns in a rural Tanzanian hospital. *PLoS One.* 2019;14(10):e0222935.
17. Ersdal HL, Mduma E, Svensen E, Perlman J. Birth asphyxia: a major cause of early neonatal mortality in a Tanzanian rural hospital. *Pediatrics.* 2012;129(5):e1238-1243.
18. Mduma ER, Ersdal H, Kvaloy JT, et al. Using statistical process control methods to trace small changes in perinatal mortality after a training program in a low-resource setting. *Int J Qual Health Care.* 2018;30(4):271-275.
19. Versantvoort JMD, Kleinhout MY, Ockhuijsen HDL, Bloemenkamp K, de Vries WB, van den Hoogen A. Helping Babies Breathe and its effects on intrapartum-related stillbirths and neonatal mortality in low-resource settings: a systematic review. *Arch Dis Child.* 2019.
20. Kc A, Wrammert J, Clark RB, et al. Reducing Perinatal Mortality in Nepal Using Helping Babies Breathe. *Pediatrics.* 2016;137(6).

Field Code Changed

Formatted: Norwegian (Bokmål)

Field Code Changed

Formatted: Norwegian (Bokmål)

Formatted: Norwegian (Bokmål)

21. Arabi AME, Ibrahim SA, Manar AR, et al. Perinatal outcomes following Helping Babies Breathe training and regular peer-peer skills practice among village midwives in Sudan. *Arch Dis Child.* 2018;103(1):24-27.
22. Bellad RM, Bang A, Carlo WA, et al. A pre-post study of a multi-country scale up of resuscitation training of facility birth attendants: does Helping Babies Breathe training save lives? *BMC Pregnancy Childbirth.* 2016;16(1):222.
23. Goudar SS, Somannavar MS, Clark R, et al. Stillbirth and newborn mortality in India after helping babies breathe training. *Pediatrics.* 2013;131(2):e344-352.
24. Musafili A, Essen B, Baribwira C, Rukundo A, Persson LA. Evaluating Helping Babies Breathe: training for healthcare workers at hospitals in Rwanda. *Acta Paediatr.* 2013;102(1):e34-38.
25. Bang A, Patel A, Bellad R, et al. Helping Babies Breathe (HBB) training: What happens to knowledge and skills over time? *BMC Pregnancy Childbirth.* 2016;16(1):364.
26. Kc A, Wrammert J, Nelin V, et al. Evaluation of Helping Babies Breathe Quality Improvement Cycle (HBB-QIC) on retention of neonatal resuscitation skills six months after training in Nepal. *BMC Pediatr.* 2017;17(1):103.
27. Arlington L, Kairuki AK, Isangula KG, et al. Implementation of "Helping Babies Breathe": A 3-Year Experience in Tanzania. *Pediatrics.* 2017;139(5).
28. Ersdal HL, Vossius C, Bayo E, et al. A one-day "Helping Babies Breathe" course improves simulated performance but not clinical management of neonates. *Resuscitation.* 2013;84(10):1422-1427.
29. Wrammert J, Kc A, Ewald U, Malqvist M. Improved postnatal care is needed to maintain gains in neonatal survival after the implementation of the Helping Babies Breathe initiative. *Acta Paediatr.* 2017;106(8):1280-1285.
30. Moshiro R, Perlman JM, Kidanto H, Kvaloy JT, Mdoe P, Ersdal HL. Predictors of death including quality of positive pressure ventilation during newborn resuscitation and the relationship to outcome at seven days in a rural Tanzanian hospital. *PLoS One.* 2018;13(8):e0202641.
31. Linde JE, Schulz J, Perlman JM, et al. The relation between given volume and heart rate during newborn resuscitation. *Resuscitation.* 2017;117:80-86.
32. Budhathoki SS, Gurung R, Ewald U, Thapa J, Kc A. Does the Helping Babies Breathe Programme impact on neonatal resuscitation care practices? Results from systematic review and meta-analysis. *Acta Paediatr.* 2019;108(5):806-813.
33. *Tanzania Mainland poverty assessment.* 2015.

Formatted: Norwegian (Bokmål)

Formatted: English (United States)

Formatted: English (United States)

Table 1: Baseline characteristics of live births at Haydom 1.7.2013-30.6.2018 (n=18,997)

	Period of birth					
	1st year n=4,662	2nd year n=3,909	3rd year n=3,565	4th year n=3,664	5th year n=3,197	p-v[illegible]
**Maternal characteristics**						
**No antenatal care, % (n)**						
	0.9 (44)	1.2 (48)	1.0 (36)	0.7 (24)	1.0 (32)	
**Antenatal problem, % (n)**						<[illegible]
	0.9 (44)	0.7 (29)	1.6 (57)	1.8 (65)	2.1 (66)	
**Preeclampsia/eclampsia, % (n)**						
	0.5 (21)	0.4 (17)	0.7 (25)	0.7 (26)	0.9 (28)	
**Delivery**						
**Mode of delivery,% (n)**						<[illegible]
**Vaginal**	78 (3,650)	76 (2,961)	76 (2,715)	76 (2,770)	75 (2,409)	
**Caesarean**	20 (924)	24 (943)	23 (824)	23 (858)	23 (749)	
**Breech**	2 (77)	0 (0)	0.5 (18)	0.5 (19)	0.8 (26)	
**Vacuum/others**	0.2 (11)	0.1 (4)	0.2 (8)	0.5 (17)	0.3 (11)	
**Fetal heart rate at admission,% (n)**						<[illegible]
**Normal**	88 (4,122)	90 (3,510)	92 (3,274)	94 (3,444)	94 (3,006)	
**Abnormal**	1 (42)	1 (33)	1 (31)	1 (38)	1 (43)	
**Not detectable**	0.04 (2)	0 (0)	0.1 (2)	0.1 (5)	0 (0)	
**Not measured**	11 (496)	9 (365)	7 (257)	5 (177)	5 (145)	
**Fetal heart rate during labour,% (n)**						<[illegible]
**Normal**	84 (3,917)	83 (3,249)	83 (2,973)	86 (3,157)	86 (2,751)	
**Abnormal**	3 (143)	4 (168)	5 (168)	6 (230)	9 (298)	
**Not detectable**	0 (0)	0 (0)	0.2 (7)	0.1 (4)	0.03 (1)	
**Not measured**	13 (602)	13 (494)	12 (416)	7 (272)	5 (145)	
**Last heart rate before delivery (bpm), mean (SD)**						
	134 (10)	134 (10)	134 (12)	134 (14)	133 (14)	
**Child characteristics**						
**Birth weight, Mean (SD)**	3215 (528)	3253 (520)	3280 (520)	3377 (539)	3342 (546)	<[illegible]
**<1500**	0.5 (24)	0.5 (24)	0.4 (15)	0.6 (23)	0.9 (28)	
**1500-2500**	7 (325)	6 (218)	6 (199)	4 (158)	4 (136)	
**2500-3499**	62 (2,895)	61 (2,397)	58 (2,071)	50 (1,821)	53 (1,690)	
**3500-4499**	29 (1,370)	31 (1,228)	35 (1,242)	44 (1,594)	40 (1,284)	
**>4500**	1 (48)	1 (42)	1 (38)	2 (68)	2 (57)	
**Gestational age < 37 weeks, % (n)**						[illegible]
	2.5 (117)	2.6 (103)	2.7 (96)	3.3 (121)	3.5 (111)	
**Female sex ,% (n)**						[illegible]
	46 (2,141)	48 (1,882)	47 (1,672)	46 (1,692)	48 (1,546)	

Formatted: English (United States)
Formatted: English (United States)
Formatted: English (United States)
Formatted: English (United States)
Formatted: English (United States)
Formatted: English (United States)
Formatted: English (United States)
Formatted: English (United States)
Formatted: English (United States)
Formatted: English (United States)
Formatted: English (United States)
Formatted: English (United States)
Formatted: English (United States)
Formatted: English (United States)
Formatted: English (United States)
Formatted: English (United States)
Formatted: English (United States)
Formatted: English (United States)
Formatted: English (United States)
Formatted: English (United States)
Formatted: English (United States)
Formatted: English (United States)
Formatted: English (United States)
Formatted: English (United States)
Formatted: English (United States)
Formatted: English (United States)
Formatted: English (United States)
Formatted: English (United States)

	Period of birth					
	1st year<br>n=4,662	2nd year<br>n=3,909	3rd year<br>n=3,565	4th year<br>n=3,664	5th year<br>n=3,197	p-v[illegible]
**Maternal characteristics**						
**No antenatal care, n (%)**						
	44 (0.9)	48 (1.2)	36 (1.0)	24 (0.7)	32 (1.0)	
**Antenatal problem, n (%)**						<[illegible]
	44 (0.9)	29 (0.7)	57 (1.6)	65 (1.8)	66 (2.1)	
**Preeclampsia/eclampsia, n (%)**						
	21 (0.5)	17 (0.4)	25 (0.7)	26 (0.7)	28 (0.9)	
**Delivery**						
**Mode of delivery, n (%)**						<[illegible]
**Vaginal**	3,650 (78)	2,961 (76)	2,715 (76)	2,770 (76)	2,409 (75)	
**Caesarean**	924 (20)	943 (24)	824 (23)	858 (23)	749 (23)	
**Breech**	77 (2)	0 (0)	18 (0.5)	19 (0.5)	26 (0.8)	
**Vacuum/others**	11(0.2)	4 (0.1)	8 (0.2)	17 (0.5)	11 (0.3)	
**Fetal heart rate at admission, n (%)**						<[illegible]
**Normal**	4,122 (88)	3,510 (90)	3,274 (92)	3,444 (94)	3,006 (94)	
**Abnormal**	42 (1)	33 (1)	31 (1)	38 (1)	43 (1)	
**Not detectable**	2 (0.04)	0 (0)	2 (0.1)	5 (0.1)	0 (0)	
**Not measured**	496 (11)	365 (9)	257 (7)	177 (5)	145 (5)	
**Fetal heart rate during labour, n (%)**						<[illegible]
**Normal**	3,917 (84)	3,249 (83)	2,973 (83)	3,157 (86)	2,751 (86)	
**Abnormal**	143 (3)	168 (4)	168 (5)	230 (6)	298 (9)	
**Not detectable**	0 (0)	0 (0)	7 (0.2)	4 (0.1)	1 (0.03)	
**Not measured**	602 (13)	494 (13)	416 (12)	272 (7)	145 (5)	
**Last heart rate before delivery (bpm), mean (SD)**						
	134 (10)	134 (10)	134 (12)	134 (14)	133 (14)	
**Child characteristics**						
**Birth weight, Mean (SD)**	3215 (528)	3253 (520)	3280 (520)	3377 (539)	3342 (546)	<[illegible]
**<1500, n (%)**	24 (0.5)	24 (0.5)	15 (0.4)	23 (0.6)	28 (0.9)	
**1500-2500, n (%)**	325 (7)	218 (6)	199 (6)	158 (4)	136 (4)	
**2500-3499, n (%)**	2,895 (62)	2,397 (61)	2,071 (58)	1,821 (50)	1,690 (53)	
**3500-4499, n (%)**	1,370 (29)	1,228 (31)	1,242 (35)	1,594 (44)	1,284 (40)	
**>4500, n (%)**	48 (1)	42 (1)	38 (1)	68 (2)	57 (2)	
**Gestational age < 37 weeks, n (%)**						[illegible]
	117 (2.5)	103 (2.6)	96 (2.7)	121 (3.3)	111 (3.5)	
**Female sex, n (%)**						[illegible]
	2,141 (46)	1,882 (48)	1,672 (47)	1,692 (46)	1,546 (48)	

bpm: beats per minute

* p-values calculated with chi-square for categorical and binary regression for continuous variables

1st year: 1.7.2013-30.6.2014
2nd year: 1.7.2014-30.6.2015
3rd year: 1.7.2015-30.6.2016
4th year: 1.7.2016-30.6.2017
5th year: 1.7.2017-30.6.2018

	Period of birth					
	1st year	2nd year	3rd year	4th year	5th year	p-value*
**Macerated stillbirths, % (n/total births)**						
	1.3 (63/4,801)	1.4 (55/4,022)	1.3 (48/3,652)	2.0 (74/3,794)	2.0 (65/3,302)	0.021
**Fresh stillbirths % (n/total births excl MSB)**						
	1.6 (76/4,738)	1.5 (58/3,967)	1.1 (39/3,604)	1.5 (56/3,720)	1.2 (40/3,237)	0.27
**Early neonatal death, % (n/total of liveborn)**						
	0.6 (30/4,662)	1.0 (41/3,909)	0.6 (23/3,565)	1.0 (36/3,664)	1.2 (37/3,197)	0.10
**FSB + Early neonatal death, % (n/total of births excl MSB)**						
	2.2 (106/4,738)	2.5 (99/3,967)	1.7 (62/3,604)	2.5 (92/3,720)	2.4 (77/3,235)	0.15

Table 2: Outcome by year including stillbirths (n=19,587)

* p-values calculated with chi-square test

*p-values calculated with chi-square test.

Formatted: English (United States)
Formatted: English (United States)
Formatted: English (United States)
Formatted: English (United States)
Formatted: English (United States)
Formatted: English (United States)
Formatted: English (United States)
Formatted: English (United States)
Formatted: English (United States)
Formatted: English (United States)
Formatted: English (United States)
Formatted: English (United States)

Table ~~3~~2: Postnatal events and heart rate recordings ~~characteristics~~ of live births at Haydom 1.7.2013-30.6.2018 (n=18,997)

	Period of birth					
	1st year	2nd year	3rd year	4th year	5th year	[illegible] value*
	n=4,662	n=3,909	n=3,565	n=3,664	n=3,197	
**Apgar <5, n (%)**						
**1 min**	37 (0.8)	50 (1.3)	42 (1.2)	45 (1.2)	66 (2.1)	[illegible]
**5 min**	5 (0.1)	7 (0.2)	11 (0.3)	20 (0.6)	23 (0.7)	[illegible]
**Stimulation/suction*, n (%)**						[illegible]
**No stim./suction**	3,950 (85)	3,234 (83)	2,412 (68)	2,684 (73)	2,180 (68)	
**Stimulation only**	15 (0.3)	36 (0.9)	100 (2.8)	159 (4.3)	186 (6)	
**Stim. and suction**	697 (15)	641 (16)	1053 (30)	821 (22)	843 (26)	
**Bag/mask ventilation**	361 (7.7)	240 (6.1)	242 (6.8)	272 (7.4)	264 (8.8)	<0.001
**Heart rate**	n=389	n=298	n=274	n=262	n=232	
**Initial heart rate beats/min median (IQR)**	145 (97-179)	142 (94-171)	132 (84-174)	118 (70-162)	102 (67-148)	[illegible]
**Initial heart rate in categories, n (%)**	32 (8)	25 (8)	21 (8)	36 (14)	50 (22)	<0.001
**<60**	69 (18)	58 (19)	75 (27)	74 (28)	63 (27)	
**60-100**	139 (36)	98 (33)	85 (31)	80 (31)	75 (32)	
**100-160**	149 (38)	117 (39)	93 (34)	72 (27)	44 (19)	
**>160**						
**Heart rate the first 60 seconds after initial recording, median (IQR)**	143 (104-165)	139 (101-165)	139 (105-165)	130 (91-157)	129 (102-152)	<0.001

IQR: Inter-quartile range
* Chi2-test used for categorical analyses, linear regression for continuous analyses. P-values for trend.
† excluding participants in the PEEP-study, n=80 and n=123 in 4th and 5th year, respectively.

	Period of birth					
	1st year	2nd year	3rd year	4th year	5th year	value*
	n=4,662	n=3,909	n=3,565	n=3,664	n=3,197	
Apgar <5, % (n)						
1 min	0.8 (37)	1.3 (50)	1.2 (42)	1.2 (45)	2.1 (66)	
5 min	0.1 (5)	0.2 (7)	0.3 (11)	0.6 (20)	0.7 (23)	
Stimulation/suction*, % (n)						
No stim./suction	85 (3,950)	83 (3,234)	68 (2,412)	73 (2,684)	68 (2,180)	
Stimulation only	0.3 (15)	0.9 (36)	2.8 (100)	4.3 (159)	6 (186)	
Stim. and suction	15 (697)	16 (641)	30 (1053)	22 (821)	26 (843)	
Bag/mask ventilation	7.7 (361)	6.1 (240)	6.8 (242)	7.4 (272)	8.8 (264)	<0.001
Heart rate	n=389	n=298	n=274	n=262	n=232	
Proportion with initial						
HR in % (n)*	145 (97-179)	142 (94-171)	132 (84-174)	118 (70-162)	102 (67-148)	
<60						
60-100						
100-160	8 (32)	8 (25)	8 (21)	14 (36)	22 (50)	
>160	18 (69)	19 (58)	27 (75)	28 (74)	27 (63)	
HR during the first 60	36 (139)	33 (98)	31 (85)	31 (80)	32 (75)	
seconds after first	38 (149)	39 (117)	34 (93)	27 (72)	19 (44)	
recording, median						
(IQR)	143	139	139	130	129	<0.001
	(104-165)	(101-165)	(105-165)	(91-157)	(102-152)	

Formatted: English (United States)
Formatted: English (United States)
Formatted: English (United States)
Formatted: English (United States)
Formatted: English (United States)
Formatted: English (United States)
Formatted: English (United States)
Formatted: English (United States)
Formatted: English (United States)
Formatted: English (United States)
Formatted: Left
Formatted: English (United States)
Formatted Table

	Period of birth					
	1st year	2nd year	3rd year	4th year	5th year	[illegible] value*
	n=4,662	n=3,909	n=3,565	n=3,664	n=3,197	
**Bag/mask-ventilation, time events**	n=358	n=234	n=240	n=269	n=262	
**Time from birth to BMV, seconds median,(IQR)**	105 (74-149)	116 (81-164)	119 (88-158)	109 (74-162)	99 (74-153)	[illegible]
**Duration of first BMV sequence, median,(IQR)**	11 (4-20)	11 (5-22)	16 (7-34)	23 (9-52)	32 (13-71)	<0.001
**Duration of BMV first 60 seconds, median (IQR)**	53 (26-59)	55 (35-59)	57 (37-59)	59 (52-59)	59 (47-59)	<0.001
**Duration of BMV in total, median (IQR)**	132 (56-248)	127 (59-340)	127 (66-298)	142 (64-282)	130 (73-301)	0.94
**Bag/mask ventilation characteristics[‡]**	n=293	n=247	n=238	n=173	n=114	
**Frequency of ventilations (inflations per minute)**	66 (50-84)	52 (39-70)	44 (35-53)	48 (41-54)	47 (41-53)	<0.001
**Tidal volume in ml/kg, median (IQR)**	5.3 (3.3-8.4)	6.8 (4.3-10.0)	7.5(5.0-11.5)	6.6 (4.5-10.7)	6.2 (3.2-9.8)	<0.001
**Mask leak in %, median (IQR)**	45 (33-62)	46 (33-61)	42 (30-57)	41 (29-56)	44 (30-57)	0.001
**Mean airway pressure mbar, median (IQR)**	14 (12-17)	13 (11-16)	15 (12-18)	17 (14-21)	18 (16-21)	<0.001
**Maximum airway pressure mbar, median (IQR)**	34 (29-38)	33 (28-36)	36 (32-39)	38 (34-43)	40(37-45)	<0.001

Formatted: Font: 12 pt, English (United States)
Formatted: English (United States)
Formatted: English (United States)
Formatted: English (United States)
Formatted: English (United States)
Formatted: English (United States)
Formatted: English (United States)
Formatted: English (United States)
Formatted: English (United States)
Formatted: English (United States)
Formatted: English (United States)
Formatted: English (United States)

Table ~~4~~3: Relative risk for early neonatal death and perinatal death at Haydom 1.7.2013-30.6.2018

	Period of birth					Overall	p-value†
**Early neonatal deaths, n/total live births**	**1st year**	**2nd year**	**3rd year**	**4th year**	**5th year**	**linear**	
	30/4,662	41/3,909	23/3,565	56/3,720	37/3,197		
**Unadjusted**							
	1 (ref.)	1.63 (1.02-2.61)	1.00 (0.58-1.72)	1.53 (0.94-2.47)	1.63 (1.11-2.90)	1.11 (1.00-1.24)	
**Adjusted for antenatal problems**							
	1 (ref.)	1.64 (1.02-2.62)	0.99 (0.58-1.70)	1.50 (0.93-2.44)	1.76 (1.09-2.84)	1.11 (1.00-1.23)	
**Adjusted for abnormal fetal heart rate**							
	1 (ref.)	1.55 (0.97-2.48)	0.93 (0.54-1.59)	1.34 (0.83-2.18)	1.44 (0.88-2.33)	1.06 (0.95-1.18)	
**Adjusted for birth weight and prematurity**							
	1 (ref.)	1.58 (1.00-2.48)	1.00 (0.59-1.70)	1.53 (0.95-2.44)	1.72 (1.08-2.72)	1.11 (1.00-1.23)	
**Adjusted for initial heart rate**							
	**1 (ref.)**	1.72 (1.09-2.70)	0.96 (0.56-1.63)	1.34 (0.84-2.14)	1.41 (0.88-2.26)	1.04 (0.94-1.16)	
**Adjusted for need for BMV**						1.09	
	**1 (ref.)**	1.88 (1.19-2.98)	1.10 (0.65-1.88)	1.57 (0.98-2.52)	1.72 (1.07-2.74)	1.09 (0.99-1.21)	
**Final adjusted model***							
	1 (ref.)	1.88 (1.11-3.19)	0.92 (0.50-1.69)	1.25 (0.72-2.18)	0.93 (0.53-1.64)	0.95 (0.84-1.07)	
**Sensitivity analysis without those not resuscitated**							
**Perinatal deaths ~~FSB+ENM~~/total births**	106/4,738	99/3,967	62/3,604	92/3,720	77/3,235		
**Unadjusted**							
	1 (ref.)	1.12 (0.85-1.46)	0.77 (0.56-1.05)	1.11 (0.84-1.46)	1.06 (0.80-1.42)	1.01 (0.95-1.08)	
**Final adjusted model***							
	1 (ref.)	1.58 (1.01-2.47)	0.88 (0.54-1.43)	0.87 (0.55-1.39)	0.53 (0.32-0.86)	0.84 (0.76-0.94)	

* adjusted for antenatal problem, abnormal fetal heart rate during labour, birth weight, prematurity, initial heart rate and need for BMV after birth.

† p-value for trend

Table 45: Predictors (relative risk with 95% confidence intervals) for perinatal death at Haydom Lutheran Hospital during five years (n=19,264).

	Unadjusted	Adjusted*
**Period, per year increase 2013-18**		
	1.01 (0.95-1.08)	0.84 (0.76-0.94)
**Fetal heart rate:**		
**normal**	1 (ref.)	1 (ref.)
**abnormal**	5.7 (4.4-7.3)	3.0 (2.0-4.5)
**not detected**	74.0 (64.5-84.9)	62.9 (20.5-193)
**Birth weight**		
**<1.5 kg**	28.7 (21.0-39.2)	23.6 (10.5-53.2)
**1.5-2.5 kg**	7.7 (5.8-10.1)	2.3 (1.3-4.1)
**2.5-3.5 kg**	1.4 (1.1-1.8)	1.0 (0.7-1.6)
**3.5-4.5 kg**	1 (ref.)	1 (ref.)
**>4.5 kg**	3.1 (1.6-5.9)	1.5 (0.5-5.1)
**Prematurity**		
	12.9 (10.7-15.6)	5.7 (3.3-9.9)
**Low initial heart rate after birth**		
**<60 bpm**	165 (131-208)	19.6 (11.1-34.9)
**60-100 bpm**	33.0 (23.3-46.6)	1.8 (1.0-3.2)
**100-160 bpm**	6.3 (3.5-11.3)	0.3 (0.2-0.8)
**>160 bpm**	2.4 (0.99-6.0)	0.2 (0.06-0.48)
**No need for recording**	1 (ref.)	1 (ref.)
**Need for BMV after birth**		
	74.0 (56.1-97.8)	28.7 (16.0-51.2)

*adjustments for fetal heart rate, birth weight category, prematurity, low initial heart rate and need for BMV after birth. In perinatal mortality FSB results the initial heart rate and presumed need for BMV were set to the lowest category.

Formatted: English (United States)
Formatted: English (United States)
Formatted: English (United States)
Formatted: English (United States)
Formatted: English (United States)
Formatted: English (United States)
Formatted: English (United States)
Formatted: English (United States)
Formatted: English (United States)
Formatted: English (United States)
Formatted: English (United States)
Formatted: English (United States)
Formatted: English (United States)

Table 5: Bag-mask ventilation characteristics of live births at Haydom 1.7.2013-30.6.2018 (n=18,997)

	Period of birth					
	**1st year**	**2nd year**	**3rd year**	**4th year**	**5th year**	[illegible] value*
	n=4,662	n=3,909	n=3,565	n=3,664	n=3,197	
**Bag/mask-ventilation, time events**						
	n=358	n=234	n=240	n=269	n=262	
**Time from birth to BMV, seconds median,(IQR)**	105 (74-149)	116 (81-164)	119 (88-158)	109 (74-162)	99 (74-153)	[illegible]
**Duration of first BMV sequence, median,(IQR)**	11 (4-20)	11 (5-22)	16 (7-34)	23 (9-52)	32 (13-71)	<0.001
**Duration of BMV first 60 seconds, median (IQR)**	53 (26-59)	55 (35-59)	57 (37-59)	59 (52-59)	59 (47-59)	<0.001
**Duration of BMV in total, median (IQR)**	132 (56-248)	127 (59-340)	127 (66-298)	142 (64-282)	130 (73-301)	0.94
**Bag/mask ventilation characteristics†**						
	n=293	n=247	n=238	n=173	n=114	
**Frequency of ventilations (inflations per minute)**	66 (50-84)	52 (39-70)	44 (35-53)	48 (41-54)	47 (41-53)	<0.001
**Tidal volume in ml/kg, median (IQR)**	5.3 (3.3-8.4)	6.8 (4.3-10.0)	7.5(5.0-11.5)	6.6 (4.5-10.7)	6.2 (3.2-9.8)	<0.001
**Mask leak in %, median (IQR)**	45 (33-62)	46 (33-61)	42 (30-57)	41 (29-56)	44 (30-57)	0.001
**Mean airway pressure mbar, median (IQR)**	14 (12-17)	13 (11-16)	15 (12-18)	17 (14-21)	18 (16-21)	<0.001
**Maximum airway pressure mbar, median (IQR)**	34 (29-38)	33 (28-36)	36 (32-39)	38 (34-43)	40(37-45)	<0.001

IQR: Inter-quartile range.
BMV: Bag-mask ventilation.
* Chi2-test used for categorical analyses, linear regression for continuous analyses. P-values for trend.
† excluding participants in the PEEP-study, n=80 and n=123 in 4th and 5th year, respectively.
Two-step analysis: Mean of ventilation parameters for each newborn during first 5 minutes of active ventilation calculated, and in the second step this mean was used to calculate median/IQR.
R2

Formatted: English (United States)

Figure text:

Fig 1: Neonatal resuscitation monitor and its components used for data collection at Haydom Hospital during 1.7.13 to 30.6.18.

Fig 21: Flow chart of participants in a population-based study of births in Haydom hospital during 1.7.2013 to 30.6.2018.

Fig 3: Early neonatal mortality and stillbirths at Haydom Hospital by year during 1.7.13 to 30.6.18.

Formatted: English (United States)

Formatted: English (United States)

Formatted: English (United States)

PONE-D-19-30351
Increased perinatal survival and improved ventilation skills over a five-year period

We would like to thank the editors and reviewers for their work and valuable comments. The editors' remarks have been accommodated. In the resubmitted version, we have incorporated changes according to the comments below, and in the following respond point-by-point to the reviewers comments.

Reviewer #1: Thank you for the opportunity to review this paper entitled, "Increased perinatal survival and improved ventilation skills over a five-year period".
This prospective analysis evaluates whether the effect of the HBB program was sustained over time.
They showed that the decrease 24h newborn mortality was sustained even improved after adjusting for covariables. They also noted an improvement on ventilation performance. The is a wealth of data collected in this project and showing feasible ways of improving neonatal survival is an important goal.

My major concern is that the is significant overlap in the mortality data with a previous published paper with only 1.5 years of additional data. I think that focusing more on the ventilation portion versus mortality would add more to the literature given that the mortality data on the majority of the mortality data of this data-set has already been published depicting the fact that the effect of the program can be sustained with frequent training.

The current manuscript adds to the previous paper by ventilation data over time, which have not been published before. In the revised manuscript. We have aimed to further highlight the changes in resuscitation performance stating clearly that this is one of the aims (introduction).

Abstract: Predictors of survival can be added to the aims as this was a big focus. In the methods, some information regarding training schedule and data analysis can be added to the methods. I think the first 2 sentences in the results regarding the change in population can be condensed. I would remove the last sentence of the conclusion.

We have included more information in the aim and the methods, and condensed the results to stay within word limits. As the study has several aims, we intend to narrow down and not include predictors of survival in the aim (end of introduction). We have removed the last sentence of the conclusion.

Introduction: I think adding the already known data regarding frequent training and the effect of HBB from reference 6 would be helpful in the intro.

We have added one sentence and an additional reference (third paragraph, page 2):

"*Frequent low-dose training is one of the key elements to improve resuscitation skills.*[6,7]»

Methods:
1. Though the frequency of the skills training is referenced to another paper, I think this paper would benefit from a brief description of the "low dose high frequency skills training" that is a major intervention thought to be responsible for the sustained outcomes. I think the same can be said for the monitoring set up – it is frustrating for me as the reader to have to

reference several articles to determine the major determinants of the methods – though these can be referenced some details are required or the paper does not stand alone.
Thank you for this suggestion. In the revised manuscript we have included more information regarding the training and monitor set-up as suggested. A figure of the monitor has been added (Fig 2).

(page 4): *Briefly, weekly simulation training was performed on manikins, assisted by local-trainers who had undergone facilitator training for the HBB program. Visiting instructors from other places in Tanzania and Norway also participated regularly.*

(page 5): *Physiological data collected from 1st July 2013 until 30 Jun, 2018 included heart rate data recorded from ECG, and ventilation parameters obtained from sensors connected to the ventilation bag using a Newborn Resuscitation Monitor (Laerdal Global Health, Stavanger, Norway) (Fig 1). The arch-shaped ECG sensor was placed over the thorax or abdomen of the newborn. Two stainless-steel discs arranged on each side of the flexible arch acted as dry electrodes. The monitor used a hot-wire flow sensor connected to the ventilation bag similar to that in the Florian Respiratory Function Monitor (Acutronic Medical Systems, Hirzel, Switzerland). Volume was calculated by flow signal integration. Pressure was measured using an MPX5010 sensor (Freescale Semiconductor, Austin, TX, USA). The HR was displayed on the monitor installed in front of the resuscitation table visible to the provider, ventilation parameters were not displayed.*

2. I would like to know from reading this paper alone the basics of the training and the set-up of the ventilation data collection. In addition I feel that the description of neonatal resuscitation performance was sparse and needed more detail.

Please refer to above corrections for training and monitor setup. Resuscitation performance has been detailed more (page 6, second paragraph).
"*The secondary outcomes were clinical performance defined by timing of events during resuscitation: Time from birth to start of BMV, the continuation of BMV by measuring duration of the first ventilation sequence before pause (>5 seconds interruption) and the fraction of time used for BMV once it had been initiated. To assess actual bag-mask ventilation performance, we studied ventilation frequency, tidal volumes and mask leak the first five minutes of bag-mask ventilation.*"

3. I think that the adjustment for vulnerability factors should go under the statistical analysis section.

We have moved the description of adjustment variables including "vulnerability factors" to the statistics section as suggested.

4. I recommend that the statistical section be reviewed by the statistician involved in the project as this section feels incomplete. For example, the categorical variable comparisons in the table was noted to be chi squared but this is not mentioned in the methods. In addition, your mortality risk ratios I would assume are based off of multi variable logistic regression given that mortality is a binary variable. Please also include the statistical software used.

The method for analysis of categorical date has been added (Page 6, last paragraph).
The regression method is not a logistic regression model with logit-link (which provides odds ratios) but as the study is a cohort design we have chosen to use a binary regression model

with log-link (which provides relative risks). This information has been added to the text. Description of selection of adjustment variables has been moved and expanded in the revised manuscript, please refer to the manuscript for details.
We have also included the statistical software:
"Data were extracted using Matlab 2019a (MathWorks, Natick, MA) and analyzed using Stata SE 15 (StataCorp. 2017. Stata Statistical Software: Release 15. College Station, TX: StataCorp LP.)»

Results:
1. The first sentence of the second paragraph read awkwardly.

We have simplified the sentence to clarify: "*During the observation period, the proportion of deliveries characterized by risk factors for adverse outcome increased."*

2. In the second sentence please give exact numbers.

This has been corrected.

3. Did you do/can you add analysis to determine if there was a significant differences between specific time points versus just an overall difference as presented? This may be helpful.

We believe that adding more details would risk to overload the presentation. However, we kindly refer to the paper by Mduma et al (ref. 6) for more details in this respect.

4. I think graphic depiction of the mortality data versus the many tables would add to the paper.

Thank you for this suggestion. In the revised manuscript the mortality data from Table 2 are replaced by a line diagram (Figure 3).

5. It would be beneficial to break up table 3 as there is so much information. For the linear regression, was the outcome modeled per year? If so the r-square value would be useful in determining the strength of the association. As noted above, the methods in general need more clarification.

We agree that Table 3 (now Table 2 and 3) was large and that breaking up to display bag/mask-ventilation in a separate table provides better readability. For the new Table 2, only two of the analyses are linear, so we chose not to include the R square.

6. Was there a qualitative portion of the respiratory data – ie was BMV indicated when administered and appropriately stopped, were TV given appropriate for the size of the infant, etc

This information would certainly be of interest. However, other studies from the research group have analyzed these data (video analysis of appropriate BMV: Haug et al, Clinical Simulation in Nursing, for Tidal volumes: J.E. Linde et al. Resuscitation 117 (2017) 80–86).

Discussion:
1. Generally the for me it makes sense for strengths and limitations to come right before the conclusion.

This could be done but preferences differ among author, reviewers and editors. We feel the logical flow in the discussion is maintained without this change.

2. I don't think the subheadings are needed

We have removed the subheadings as suggested.

3. I do not think the 3rd paragraph under comparison with other studies adds much to the discussion, and could be removed.

We have removed this sentence as suggested.

4. Can you comment on the fact that continued exposure to higher acuity resuscitations may alone have some impact on these skills.

Thank you for this comment: In a qualitative study from Haydom hospital, the midwives have indicated that feedback from the monitor improves their skills. We have included a new sentence:
"Heart rate feedback during resuscitation may also have improved skills over time, as suggested in a qualitative study from Haydom.[7]*»*

5. Can you say that this is the first study that shows the long-term sustained effects if your reference 6 by the same groups shows this as well (acknowledged that yours is an additional 18mo follow-up).

We have modified this sentence, as the current study adds more data and time but the previous study also has long-term data. Instead, we include that the study is the first to monitor changes in ventilation performance with physiological data over time.
"…and provides information on perinatal mortality, resuscitation performance and physiological data during a five-year period"

6. I think it would be helpful in the discussion to add more on the simulation/training aspect with the ventilation outcome with respect to other studies.

We have added new text to several of the paragraphs to highlight the improved resuscitation performance over time, likely due to a high focus on this at the site.

Reviewer #2: Nicely done paper. I've made comments on the attached file. In brief I had a few questions:

- Can you describe who provided the "low-dose, high-frequency skills training"? Was this done by local personnel, or did it involve visiting instructors from Europe, the USA, etc.?

We have included more information regarding the low-dose, high-frequency skills training in the revised version: Local personnel were trained as HBB-trainers, and some were also facilitated by visiting instructors.
"Briefly, simulation training was performed on manikins, assisted by local-trainers who had undergone facilitator training for the HBB program. Visiting instructors from other places in

Tanzania and Norway also participated regularly. The trainings were intended to take place weekly, but with some variations over time.»

- Can you provide data on emergency vs scheduled/repeat C-Section rates over time? This would be helpful in understanding the changing risk profile of your OB population.

We have some information regarding planned vs emergency causes of C-Section over time. However, almost all C-Sections are emergency C-Sections, and the data regarding causes of emergency C-Sections do not provide sufficient quality to answer this question.

- Can you comment on why year 2 was so much worse than other years (Table 4)?

We have also seen this fluctuations over time, with year 2 as an outlier. The resuscitation performance (Table 2 and 3) was relatively similar in year 2. The HBB training varied during the 5-year period, and with shifts in the staff the quality of birth care and post-natal care is likely to have varied though not directly reflected in the data on resuscitation performance. We have added comments on this in the discussion, underlying factors not recorded in our data are likely needed to explain this change.
"The intensity and frequency of HBB training varied during the 5-year period, and due to shifts in the staff the quality of birth care and post-natal care is likely to be vulnerable. The substantial fluctuation over time in perinatal death rates, was however not reflected in the data on resuscitation performance."

- Do you know if the increased use of BVM was all due to appropriate and indicated use? For example, it appears that incidence of BVM was 4-fold the incidence of 1-min Apgar < 5, & 10-fold the incidence of 5-min Apgar < 5 (Table 3).

In this setting, research from our group has documented that the use of Apgar is highly inaccurate (Ersdal et al, Pediatrics 2012; 129: e1238-1243): 50% of those who died secondary to asphyxia had received a 5-minute Apgar score of ≥7. Thus, the Apgar scores are highly inaccurate and likely to explain this discrepancy. We have included a comment and reference to this finding.
"An increased use of BMV ventilation over the 5-year period reflects the higher morbidity, and Apgar scores in this setting have previously been shown to have low prognostic value."

- Can you comment on any potential downsides of HBB, for instance, the incidence of Pneumothoraces?

We unfortunately do not have access to portable chest x-rays at the hospital, so pneumothoraces are not possible to confirm accurately. Potentially, the focus on stimulation and suctioning from the start of the HBB program may have resulted in unnecessary suctioning that is now discouraged in resuscitation algorithms. We have added the following:
"However, we cannot rule out that a sustained focus on HBB training may increase unnecessary resuscitation procedures."

Reviewer #3: Stordal and colleagues present an observational study reporting on perinatal survival and ventilation skills over a five year period of ongoing training following an initial intervention associated with the Helping Babies Breathe program.
Strengths of this study include the prospective nature, the large and relatively complete data obtained, and the inclusion of physiological data to assist in explaining the mortality

outcomes. Key limitations of the study include confounding associated with a change in health funding that altered the complexity of neonatal admissions. This confounding required results to undergo statistical adjustment for increasing vulnerability of the infant case mix, to demonstrate an annual reduction in relative risk for perinatal death.
An annotated manuscript is uploaded to assist authors with their revisions. This manuscript makes some suggestions for improved text – but I would strongly recommend the authors to seek professional English editing: overall I think the text length could be reduced by at least 10 % with more efficient word use, and clearer sentence structure.

Thank you for this comment, we have revised the text according to the annotated manuscript and aimed to make the revision more to-the-point. We have also revised the language according to the comments.

MAJOR COMMENTS:
The authors should consult the STROBE guidelines for reporting an observational study.
It would be preferable to make deidentified data available for transparency. De-identified data should not compromise any data privacy restrictions.

Data are available upon specific request. However, we are not allowed to make these openly available due to regulations from the National Institute of Medical Research in Tanzania.

Specific statements regarding Financial Disclosure, Competing Interests and Ethics statements should be entered in line with suggested response types in the online submission form rather than just stating "no" or "as stated in the manuscript".

This has been corrected.

TITLE: the study design should be indicated in the title and abstract
In the title we have now included: An observational study

ABSTRACT
Background/aim: - the years of the initial program should be studied.
The years of the initial program have been included.

Methods should summarise statistical methodology including method used for adjustment of outcomes. rather than refering loosely to analysis of changes over time… (e.g., prospective observational cross-sectional cohort study).

In the revision we have added "…*We analyzed changes over time in outcomes, use of resuscitation interventions and performance of resuscitation using regression models to obtain adjusted relative risks.*

Results – please see suggestions for adjusted wording of the last few sentences.

In the revision we have changed the wording of the last few sentences to: "*During the five-year period, longer duration of bag-mask ventilation sequences without interruption was observed. Delivered tidal volumes were increased and mask leak was decreased during ventilation.*"

Conclusions – please see suggestions for adjusted wording. The final conclusion is not justified without data relating to population level neonatal mortality/morbidity over the same interval (i.e., was not being born in a hospital associated with increased neonatal mortality.

We have modified the wording of the conclusion, and removed the last sentence as we do not have population-based data of perinatal mortality for the region.
"*The reduction in 24-hour newborn mortality after introduction of Helping Babies Breathe was maintained, and a further decrease over the five-year period was evident when analyses were adjusted for vulnerability of the newborns.*"

INTRODUCTION
Overall – the rationale for the study is not well presented. If the HBB decreased mortality, the authors need to justify why a follow-up study was necessary and what it aimed to achieve.
P9, Sentence 1 and 2: It would be helpful to present the fall in global mortality in both 1-5 years and neonatal population to support the statement in the first sentence that reduction in neonatal deaths has been slower over the last 2 decades

The phrasing has been changed according to suggestions. We have added accurate numbers for comparison between neonatal and post-neonatal mortality rates, please refer to the revised manuscript.

P9 final paragraph. This paragraph is unclear. The abstract suggests 1 follow-up study 2013-2018 and previous paragraphs in the introduction suggest the HBB program commenced in 2010 with the initial study extending for 24 months This paragraph suggests a study 2011-2016, and another study with ongoing onsite-low-dose HF HBB. The crossover in dates is confusing to the reader and it is not clear if this is a hybrid of the initial study or a separate follow-up study. I suggest the authors revise the wording of this paragraph to make it explicity clear when the five year study was completed and the timing of the initial study. This paragraph should provide a hypothesis for the study.

We have changed the wording to clarify the periods of time included in our study:
"*After introduction of HBB in 2009-2012, efforts to sustain the initial improvements in perinatal mortality continued with onsite low-dose high-frequency HBB training. In the present study, we aimed to document changes in 24-hour neonatal mortality, fresh stillbirths and resuscitation performance during a five-year period from 2013 to 2018.*"

METHODS
Overall, the methodology section appeared to include elements of the previous study (2010-2011) and the current 2013-2016 study which was confusing to read. As this paper is about the 2013-2018 study, the methodology should exclusively relate to what was done in this study, with a citation used to reference back to the previous study.
Key elements of the study design and ethical approvals should be provided early in the methodology section.
The setting should simply state the hospital/location and relevant dates (including day not just month and year) for recruitment and data collection. Detail about fees for delivery and the effect on hospital delivery numbers should be part of a discussion.

We have moved the section of ethical approvals to the end of the first paragraph.

Further, we have divided with inclusion of separate headlines the text describing the initial HBB study and the Safer Births study with exact time period and interventions. The text has been revised according to suggestions from the reviewer, please refer to the "track changes" document. The reduction in births and user fees is moved to the discussion.

Participants should be described including eligibility and exclusion criteria

All births in the hospital including stillbirths are recorded in the dataset. This is stated in the revised manuscript: "*All births in the hospital from 1st of July 2013 until 30th of June 2018 were recorded in the study.*"

Outcomes should be described separately to statistical methodology/treatment, and any explanation/justification of the methodology should go into the discussion.
Statistical elements that are missing include management of missing data, methods to examine subgroups/interactions, how confounding variables were managed, the approach to regression modelling and treatment of collinear variables (e.g., birthweight and gestation).

The outcomes are described under a separate headline, now also including the secondary outcomes of resuscitation performance.
Exposure follows this section, as shown in the revised manuscript ("track changes"). We have added adjustment for type of bag used during the study period as explained.
In the statistics section we have added information regarding handling of missing data, adjustment for confounding and regression models. Subgroup or stratified analyses could be done, but would add further complexity to the presentation. Therefore we prefer to adjust for subgroup characteristics (for example term/preterm birth). The term "gestational age" appears once in the results section, but the correct term should be "prematurity": Gestational week was not used (this has not been recorded with sufficient precision), only as a dichotomous variable for preterm/term birth. With a Spearman rho of 0.2 between prematurity and birth weight, collinearity does not preclude the analyses.

RESULTS
The exclusion of premature infants from the analysis is not well justified – death in the first 24 hours from prematurity is most often related to respiratory distress and may stem from insufficient resuscitation.
We include all – also preterms - in the main analyses. The analysis excluding those that had not been ventilated at all is performed as a sensitivity analysis only, and this is clarified in the revised manuscript. Whether any of these should have been ventilated is possible, however other causes of death including sepsis or severe prematurity are certainly likely in this category. We have modified the wording to clarify, and added the results from this sensitivity analysis to Table 3.

Some results are presented without accompanying statistics
The numbers from statistical analyses have been added to the text where appropriate, in addition to the original version displaying this in Tables.

It is not clear why infants involved in the PEEP trial were excluded from the ventilation analysis but not from the mortality outcomes, given PEEP may alter effectiveness of resuscitation and hence the resulting mortality.

Thank you for this important comment. The type of bag used did not predict mortality (p=0.25) and therefore did not satisfy the criteria for inclusion in the adjusted model. This is now explained in the Methods/statistics section.

Ventilation frequency should be presented as inflations/min or breaths/min not as 60/min
Tidal volume was corrected for weight and this should be clear in the results.

We have changed this in the revised manuscript.

DISCUSSION
Paragraph 1: suggest that a full stop is provided after "user fees" and that the rest of the sentence becomes a new sentence as "Consequently, hospitalised newborns included a higher proportion of infants with increased vulnerability for adverse outcomes".

We have revised this paragraph according to suggestions.

Reference to the previous study would be better phrased as "before and after initial implementation of the HBB program was 11.1/1000 and 7.2/1000 respectively. The following sentence could be revised to indicate that reduction in early neonatal mortality accounted for the majority of the overall decrease in baseline perinatal mortality from 27/1000 to 21.7/1000 over the same period.
Thank you for this suggestion, we have rephrased and added the number showing that decrease in ENM accounted for most of the reduction in perinatal mortality:
"The baseline perinatal mortality was 27/1000 and fell to 21.7/1000, and the decrease in ENM thus accounted for 74% of the reduced perinatal mortality"

It is not clear why the authors say that the period of observation started 2 years after regular HBB implementation and extended for a further 1.5 years, when the actual study went for 5 years. The numbers don't add up?

We have clarified in the revised manuscript, stating time periods clearly in the Methods section and highlighting what is new in the present study.
"In the present follow-up, our period of observation starts two years after regular HBB implementation and provides information on perinatal mortality and resuscitation performance during a five-year period"

When other studies are quoted, the discussion would have more impact if the information was synthesised and presented as a comparison to the current data to indicate what the literature is telling us overall about the issue at hand. Reference to the study from Nepal does not indicate what the intervention was that led to a reduction of deaths from intra-partum complications but didn't change overall neonatal mortality, and hence the reader is left confused about the relevance of this information.

We have added to the discussion points that we believe are relevant to explain why HBB implementation have different impact across countries (page 12 and 13).

It was not clear that the increase in (weight corrected) tidal volumes was due to the HBB training or due to the change in patient mix. It is odd that the increase in tidal volume during resuscitation was not matched by an increase in heart rate. How do the authors explain this?

The data shown for HR in Table 2 are absolute numbers of the first recorded heart rate and during the first 60 seconds thereafter in order to characterize vulnerability of the newborns over time. Thus, the HR shown is not after one minute of BMV, which is now stated more clearly in the revised table 2.
"Mean heart rate during 0-60 seconds after initial recording, median (IQR)»

MINOR COMMENTS
Numbers < 10 should be written in full
This has been corrected.

Overall, there are too many unnecessary abbreviations in the manuscript that make it difficult to read and digest.

We have replaced FSB and MSB with the phrases "fresh stillbirth" and "macerated stillbirths" throughout.

Table 1 – data are usually presented as n (%) rather than % (n). As it is not clear what the numbers are for the child characteristics weight ranges it would be best to indicate in the table legend at the bottom of the table that data are presented as % (n) unless indicated otherwise. Any abbreviations (e.g., bpm) need to be explained in the table legend (which goes for other tables also – e.g., MSB in Table 2).

We have changed the tables according to this comment to indicate % (n). The legend includes any abbreviation, and MSB is not used now.

Table 5 needs to indicate what the numbers are – presume relative risk and 95 % CI?

Correct, Table 5 (now Table 4) is relative risk and 95% confidence interval as now included in the headline.
