# Supplementary material for: Increased perinatal survival and improved ventilation skills over a five-year period: An observational study
Source: PLoS One. 2020 Oct 12;15(10):e0240520. doi: 10.1371/journal.pone.0240520 (PMC7549771; doi:10.1371/journal.pone.0240520)
Supplement: Attachment — Submitted filename: Rebuttal letter v2 PONE.docx [file pone.0240520.s004.docx]

PONE-D-19-30351
Increased perinatal survival and improved ventilation skills over a five-year period

We would like to thank the editors and reviewers for their work and valuable comments. The editors’ remarks have been accommodated. In the resubmitted version, we have incorporated changes according to the comments below, and in the following respond point-by-point to the reviewer’s and editor’s comments.

Reviewer #1: Thank you for the opportunity to rereview this paper entitled, “Increased perinatal survival and improved ventilation skills over a five-year period”.
I have reviewed the responses and changes made, and am satisfied with the revised manuscript.

Thank you for this positive remark.

Reviewer #2: Thanks for responding to our prior editorial comments. I still have a few questions as indicated on the attached PDF.
I think you should clarify your description of the amount of BMV the subjects received. In methods, you indicate your analyzed this over the first 5 minutes, but you only present data on the first 60 seconds.

Thank you for allowing to clarify this point. The total duration of any BMV was analyzed for the full duration of resuscitation, but pressure, frequency and mask leak was restricted to the first five minutes. This has been described more clearly in Methods (Page 7):

*“The total duration of BMV was calculated as the time from the first to the last inflation.”*

Please clarify how "duration of the first BMV sequence increased" & "BMV was continued for an increasing part of the first 60 seconds", & yet the duration of BMV did not increase?

In our analyses we have shown ventilations the first 60 seconds after initiation of BMV separately, as this time frame is emphasized in the Helping Babies Breathe training. We show that the duration of the first sequence without interruptions increases and that a larger proportion of the first 60 seconds is used to ventilate. However, the overall duration of BMV did not increase. To further clarify this we have included the numbers for total duration of ventilation in the revised text, and also emphasized that overall duration of BMV did not increase (Page 10). We have also added in Table 5 a row indicating the number of BMV sequences and the median duration of these BMV sequences to illustrate the change in BMV time:

“However, the overall duration of ventilation from the first to the last inflation remained stable around a median of 130 seconds (range 127-142 seconds), explained by a higher number but shorter duration of BMV sequences in the first years (Table 5).*”*

In Table 5, you refer to a "PEEP-study" but you've eliminated any other mention of this study - you need to clarify that.

In order to clarify and give a complete picture, we have now included a reference to a randomized trial that just has been published on the use of a PEEP valve in bag-mask-ventilation. (ref #34), below Table 5:

*“excluding participants in a randomized study using positive end-expiratory pressure in newborn resuscitation, n=80 and n=123 in 4^th^ and 5^th^ year, respectively. This study is reported and described elsewhere.^34^”*

This study found differences in pressures, mask leak and tidal volumes with the use of PEEP, thus we exclude those from the mentioned analyses. However, the study did not find any differences in mortality or HR responses with the use of PEEP, and thus we do not exclude those ventilated with PEEP from the overall analyses in the present study.

There is a truly dramatic increase in the amount of "stimulation" used for resuscitation (Table 2); please comment on that. Was this in part due to the training?

Use of stimulation increased during the implementation of Helping Babies Breathe, which has been reported also in the initial study of HBB roll-out in Tanzania (ref #5) and at Haydom (ref. #11). It is likely that further training in HBB was a driver behind this increase in stimulation, but increased vulnerability is likely to contribute. We discuss this observation in the last paragraph on Page 13, but have added the following to the Results section of the revised manuscript: Page 10

*“Use of stimulation increased substantially (Table 2).”*
